# Reviewing the association between motor competence and physical activity from a behavioral genetic perspective

**DOI:** 10.3389/fpsyg.2025.1480631

**Published:** 2025-04-28

**Authors:** Yahua Zi, Eco J. C. de Geus

**Affiliations:** ^1^Department of Biological Psychology, Faculty of Behavioural and Movement Sciences, Vrije Universiteit, Amsterdam, Netherlands; ^2^School of Exercise and Health, Shanghai University of Sport, Shanghai, China; ^3^Amsterdam Public Health Research Institute, Amsterdam University Medical Center, Amsterdam, Netherlands

**Keywords:** motor development, Stodden model, twin studies, perceived motor competence, cardiorespiratory fitness, muscular fitness

## Abstract

A much-cited model by Stodden and colleagues has proposed motor competence to be a 17 promising target for intervention to increase childhood physical activity. Motor competence is thought to influence future physical activity through bidirectional causal effects that are partly direct, and partly mediated by perceived motor competence and physical fitness. Here, we argue that the model is incomplete by ignoring potential confounding effects of age-specific and age-invariant factors related to genetics and the shared family environment. We examined 106 systematic reviews and/or meta-analyses on the Stodden model for the mention of familial confounding. These reviews summarized data from 1,344 primary studies on children in the age range 0–18 on the associations in five bidirectional pathways: motor competence—physical activity, motor competence—perceived motor competence, perceived motor competence—physical activity, motor competence—physical fitness, and physical fitness—physical activity. We show that a behavioral genetic perspective has been completely lacking from this vast literature, despite repeated evidence for a substantial contribution of genetic and shared environmental factors to motor competence (*h*^2^ = ♂55%—♀58%; c^2^ = ♂31%—♀29%), physical fitness (*h*^2^ = ♂65%—♀67%; *c*^2^ = ♂3%—♀2%), and physical activity (*h*^2^ = ♂37%—♀29%; *c*^2^ = ♂33%—♀49%). Focusing on the alleged causal path from motor competence to physical activity, we find that the systematic reviews provide strong evidence for an association in cross-sectional studies, but weak evidence of prediction of physical activity by motor competence in longitudinal studies, and indeterminate effects of interventions on motor competence. Reviews on interventions on physical activity, in contrast, provide strong evidence for an effect on motor competence. We conclude that reverse causality with familial confounding are the main sources of the observed association between motor competence and physical activity in youth. There is an unabated need studies on the interplay between motor competence, perceived motor competence, physical fitness, and physical activity across early childhood and into adolescence, but such studies need to be done in genetically informative samples.

## 1 Introduction

### 1.1 The importance of physical activity in children and adolescents

The paramount importance of regular physical activity (PA) to enhance children’s health has been extensively documented (Elhakeem et al., 2018; Janssen and Leblanc, 2010; Jose et al., 2011; Kaplan et al., 1996; Leskinen et al., 2009; Wendel-Vos et al., 2004). The well-established effects of physical activity have led to the development of physical activity guidelines for youth, widely adopted across the globe (World Health Organization, 2020). Despite this, and the many active policies supporting an increase in physical activity in various settings, the majority of children and adolescents does not meet recommended physical activity levels (Guthold et al., 2020). Furthermore, as children move through childhood and adolescence towards adulthood, physical activity participation rates tend to further decline (Conger et al., 2022).

Of note, these general epidemiological trends describe what happens to the average child but fail to address the large individual differences in physical activity behaviors. These individual differences have been shown to be remarkably stable throughout the lifespan (Breau et al., 2022; Telama et al., 2005; van der Zee et al., 2019) such that children who start out to be more physically active in childhood tend to remain more active later in life. This ‘tracking’ of physical activity suggests that it would pay off to increase the number of active children to arrive at larger numbers of adolescents and adults meeting the recommended physical activity levels. Not surprisingly therefore, much effort has been spent on identifying modifiable determinants of childhood physical activity. One of the more promising traits investigated is motor competence (Øglund et al., 2015; Øglund et al., 2014). Globally, children (3–10 years) demonstrate “below average” to “average” motor competence levels (Bolger et al., 2021), suggesting that there is room for improvement of this trait by targeted intervention. However, such intervention is only meaningful to increase youth physical activity levels to the extent that motor competence has a causal effect on physical activity.

### 1.2 The role of motor competence in physical activity: the 2008 Stodden model

Motor competence can be defined as the full complement of a person’s motor abilities needed to execute all forms of goal-directed motor acts necessary to manage everyday tasks (Bolger et al., 2021; Henderson and Sugden, 1992). The potential role of motor competence for physical activity received a large boost with the development of the “Stodden model” by Stodden et al. (2008). The Stodden model identifies motor competence as a main determinant of youth and adolescent physical activity, a basic idea foreshadowed by the earlier work of Hands and Larkin (2002). To be physically active as they grow older, children need fundamental motor skills like running, jumping, catching, and throwing. Children that start out with low actual and perceived motor competence may not engage in sufficient physical activity to develop the motor competence and physical fitness needed to engage in the required level of physical activity during middle and late childhood. This will draw them into a negative spiral of disengagement in which the lower levels of physical activity in turn will amplify their motor skill deficits compared to their more active peers. “This will ultimately result in high levels of physical inactivity and will place these individuals at risk for being obese during later childhood, adolescence, and adulthood. “(Stodden et al., 2008, p. 297).

Three characteristics of the Stodden model turn it into a dynamic but complex model that make it difficult to predict the development of stable physical activity habits as well as moments that would be optimal for change by intervention. First, it adds two mediational pathways, acting through physical fitness and through perceived motor competence, to the direct pathways between motor competence and physical activity. Second, it suggests non-recursive, reciprocal effects in the direct and mediated pathways. Third, the model allows changes in the direction of effects in the pathways as a function of age. While being very complete by incorporating age-moderation, bidirectionality, and mediation, the Stodden model at the same time is undercomplete by solely focusing on the possibility that these pathways reflect *causal* effects.

### 1.3 The potential confounding by familial factors in pathways of the Stodden model

**Figure 1:**
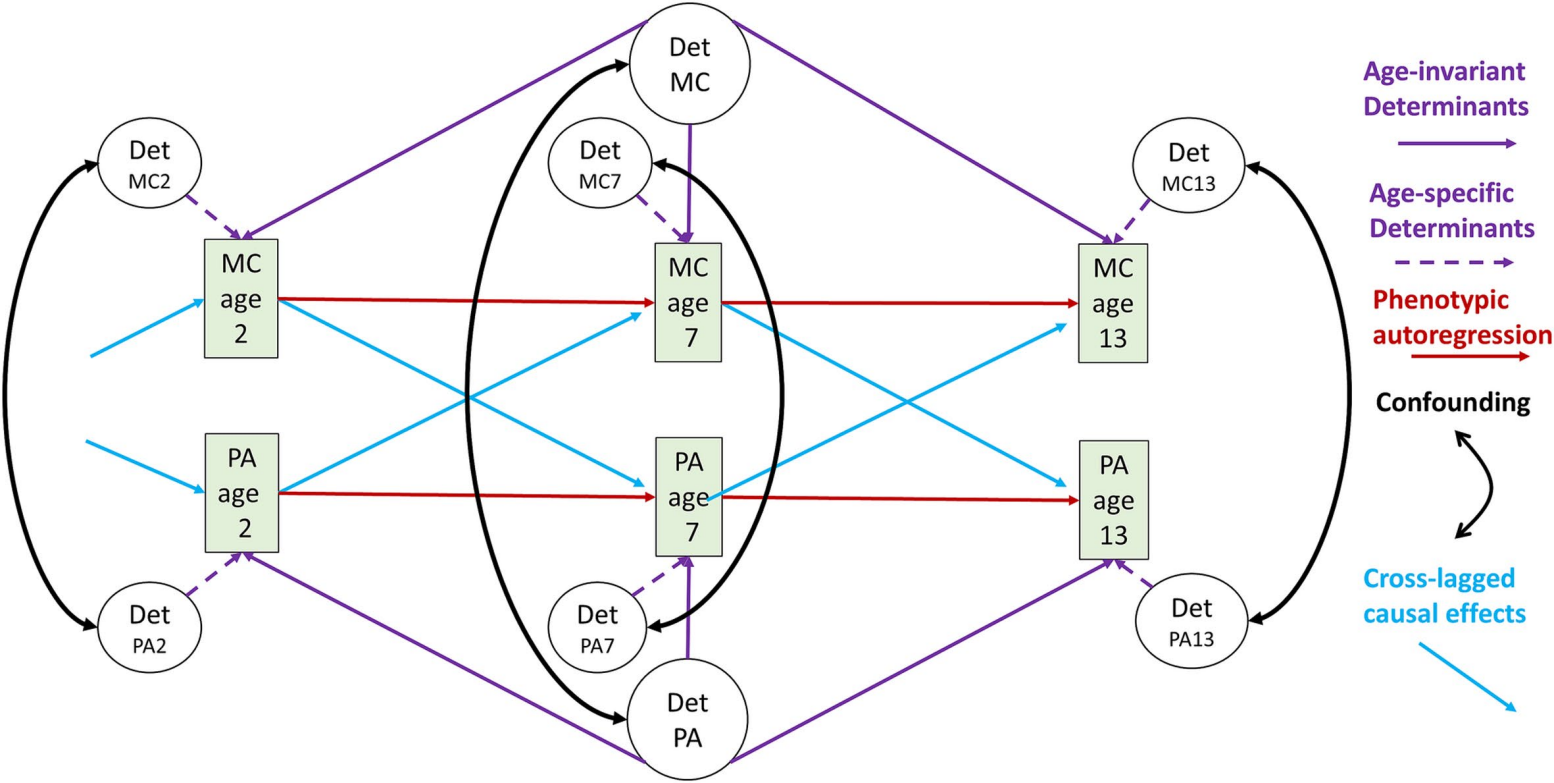
Sources of the association between motor competence and physical activity and its stability over time. Rectangles contain the observed values of the motor competence (MC) and physical activity (PA) traits at the three example ages (2, 7, 13). Ovals contain the set of latent determinants of these traits (DetMC, DetMC2, DetMC7, DetMC13 and DetPA, DetPA2, DetPA7, DetPA13) which may be genetic or environmental determinants. DetMC2, DetMC7, DetMC13 contain the age-specific latent genetic/environmental determinants of motor competence operating on MC at age 2, 7 and 13, respectively. DetMC contains the age-invariant latent genetic/environmental determinants that operate on the traits at all ages. Similar applies to DetPA, DetPA2, DetPA7, DetPA13. Dotted purple arrows from DetMC2, DetMC7, DetMC13 and DetPA2, DetPA7, DetPA13 reflect age-specific effects of these determinants, whereas the solid purple arrows from DetMC and DetPA reflect age-invariant effects of these determinants. Double-headed black arrows indicate correlation of the underlying determinants, which leads to confounding in the MC-PA associations. Red continuous lines indicate the autoregression of the motor competence and physical activity traits across time. Blue arrows indicate true causal effects of motor competence on physical activity, or in reverse, physical activity on motor competence. Blue arrows pointing into age 2 reflect causal effects from earlier ages.

In its essence, the Stodden model revolves around a set of five associations between motor competence and physical activity, between motor competence and perceived motor competence, between perceived motor competence and physical activity, between motor competence and physical fitness, and between physical fitness and physical activity (MC-PA, MC-PMC, PMC-PA, MC-Fitness, Fitness-PA). These associations can arise through fundamentally different mechanisms governing the development and the ensuing stability of the associations between the traits as well as the changes in these associations over time. Figure 1 depicts potential sources of the association between motor competence and physical activity at each of three different ages, and how these sources can impact on the stability of these associations across developmental time. To maintain intelligibility, the Figure greatly simplifies the continuous nature of development by using discrete ages 2, 7, and 13, rather than a more fine-grained model that uses steps of, e.g., 2 months. It’s aim is merely to provide an illustration of the complexity of interpreting (longitudinal) associations.

As a major innovation to the Stodden model, Figure 1 adds latent determinants that may act as confounders of the associations between motor competence and physical activity, or its putative mediators, perceived motor competence and physical fitness. Two sets of latent determinants have been repeatedly nominated by the field of behavior genetics to play a role in many developmental traits. The first set of determinants consists of the common or shared environment that contains all factors shared by family members living in the same household, including the physical home environment, family warmth and mutual support, parenting style and example setting, neighborhood characteristics, and socioeconomic status (including education level of the parents). The second set consists of the genetic variance shared by family members which may reflect additive trait effects of the two parental alleles in a gene, or non-additive trait effects due to allelic dominance or allelic interaction (epistasis).

Starting at the top of the model shown in Figure 1, we see that motor competence at age 2 (“MC age 2”) is considered to be influenced by latent determinants (“Det MC2”). These may involve genetic variants that influence sensorimotor brain functioning and neuromuscular control, or differences in motor skill challenges related to the family that children grow up in or other environmental factors such as climate or exposure to structured physical education in school or childcare settings. The influence of these genetic and environmental determinants of motor competence can show substantial stability over time (reflected in the purple arrows emanating from the latent “Det MC” factor that influences motor competence at *all* ages) because the genetic code does not noticeably change after conception, and influences related to parental rearing styles, and neighborhood or household characteristics can also be stable. However, the influence of some of the latent determinants may be confined to specific ages (reflected in the latent “Det MC2 … Det MC13” factors). For example, parental social support effects may be strong at ages 2 and 7 but become more diluted when children enter secondary school. While some genetic variants may be expressed at all ages, other variants may show age-specific (suppression of) gene-expression as part of maturation. At the bottom of the figure, we see a parallel situation for physical activity, again with both age-invariant and age-specific latent factors influencing physical activity behaviors at the three ages depicted.

At each age, an association between motor competence and physical activity may arise entirely through the correlation of the age-invariant and/or age-specific factors, without the need for a direct causal path between the two traits. For example, part of the many genetic variants that influence motor competence may overlap with those influencing physical activity, creating horizontal genetic pleiotropy when they influence these traits through *independent* routes (Minica et al., 2020; Minica et al., 2018; Solovieff et al., 2013; Verbanck et al., 2018). Likewise, environmental risk factors like household poverty and parental rearing styles may independently restrict motor competence development and reduce opportunities for regular physical activity. If the effects of genetic or environmental factors change in strength from childhood to adolescence, they may also cause a strengthening or weakening of the associations over time. Such a confounder-induced age-related change in the association would not be discriminable from an age-moderation effect on the putative causal pathway between motor competence and physical activity.

The confounding genetic or environmental effects may work directly on the two traits themselves, but also make use of an intermediate trait that itself exerts a causal effect on both traits. A first example would be that the same genetic variants that influence motor competence also influence physical activity through their effects on the dopaminergic brain systems that influence motor control as well as exercise reward pathways. A second example of such confounding would be that an obesogenic family environment would increase body mass index (BMI), with BMI having effects on both motor competence and physical activity. In short, cross-sectional associations between motor competence and physical activity at each age can reflect confounding by correlated determinants, which may be genetic or environmental in nature.

However, the effect of the latent underlying factors does not rule out the additional existence of causal effects of motor competence at an earlier age on current physical activity. These causal effects are reflected in the cross-lagged paths of Figure 1. For example, physical activity at age 7 may, in part, depend on the ability to perform basic motor actions at a sufficient level to engage in active play with parents, siblings, or peers at school from age 2 to 7. Conversely, the lagged causal effect may also work in the other direction. Daily engagement in physical activity from age 2 to 7, i.e., playing regular ball games in preschool, may actively contribute to building up motor competence, i.e., lead to increased kicking/throwing skills, at age 7. Such bidirectional causal mechanisms are suggested by the Stodden model as the main cause of the association between motor competence and physical activity in middle and late childhood.

Apart from the mechanisms inducing cross-sectional associations at each age, Figure 1 also depicts the mechanisms that lead to stability of the association of motor competence and physical activity over developmental time. A first mechanism causing stability of the associations between motor competence and physical activity is the autoregression of each of the traits separately. Substantial evidence shows that, even if absolute levels show large maturational changes, the individual differences in both motor competence and physical activity are stable across time (Barnett et al., 2010; Branta et al., 1984; Farooq et al., 2020; Malina, 1990; McKenzie et al., 2002; Pereira et al., 2022; Schmutz et al., 2020). This ‘tracking’ of motor competence and physical activity may arise from direct causal influences of the trait level at a starting age on the trait level at a later age. For example, once a neuromotor skill has been mastered (running, balancing on a beam) it will not be easily lost, and habit formation may solidify physical activity behaviors once these have been taken up in an initial period (Rebar et al., 2024).

Autoregression can be a first source of stability of the association of motor competence and physical activity over time. Once an association has come into existence, e.g., is bootstrapped at age 2 by correlated underlying determinants, it will be propagated across time sheerly by the stability in each of the two traits. A second mechanism that leads to stability of the association of motor competence and physical activity over time are the causal effects of motor competence on physical activity and the reverse causal effects of physical activity on motor competence. Finally, a third mechanism causing stability of the association is a correlation of the age-invariant genetic or environmental determinants of motor competence and physical activity (“Det PA” and “Det MC” in Figure 1). These will not just induce cross-sectional association but also longitudinal associations between motor competence and physical activity.

The Stodden model was created when the associations between the traits in the five pathways (MC-PA, MC-PMC, PMC-PA, MC-Fitness, Fitness-PA) were mostly observed in cross-sectional studies. Cross-sectional studies cannot discriminate between the mechanisms outlined in Figure 1 and outlined above. Nonetheless, if one of the hypothesized associations in the Stodden model is found to be absent, it would at once tell us that no causal effect is likely to exist. In that sense, cross-sectional studies are vital in first demonstrating the primary possibility of a causal association. Longitudinal studies are a step up from cross-sectional studies in that they establish the presence of cross-time (lagged) associations between the traits and can rule out reversed causation. If the assumed causal trait (e.g., motor competence at an early age) is seen to predict the assumed caused trait (e.g., physical activity in adolescence) in the future but in parallel, the association is not seen to hold in the opposite direction this would falsify reverse causation of motor competence by physical activity.

The strongest design to show true causality in the pathways of the Stodden model is the intervention design, where either motor competence or physical activity are manipulated, and it is tested whether the induced changes in one trait led to changes in the other trait. Well-conducted RCTs remain the highest level of evidence for a true causal effect. However, large individual differences can be seen in the response to intervention and it is not always clear what is driving these differences (Kennedy et al., 2021; Liu et al., 2024; Ma et al., 2021; Prochaska et al., 2008). Again familial factors are a potential source of the heterogeneity in responding to intervention. Attempts to increase motor competence and physical activity may fall on more fertile ground in some children compared to others, simply based on their genetic abilities and/or more supportive family environment. So, to further add to complexity, the underlying determinants of motor competence and physical activity in Figure 1 may partly act through their moderating effects of (parental or school-based) attempts to change these traits.

If the genetic and shared environmental determinants independently influence motor competence and physical activity behavior, the size of the causal effects hypothesized to underlie the observed association between motor competence and physical activity behavior would be incorrectly estimated from the size of the association when this confounding is not taken into account. Since the publication of the Stodden model, a very large amount of systematic reviews with or without meta-analyses have been published on one or more of the Stodden pathways. A reasonable expectation, therefore, is that this large volume of work has duly taken the potential of familial confounding into account. Cursory inspection of some highly cited reviews (Barnett et al., 2022; De Meester et al., 2020; Engel et al., 2018; Figueroa and An, 2017) suggested that this might not be the case, but more systematic inspection of the large volume of systematic reviews is needed. In addition, for familial confounding to be a potential issue, it is required that the traits in the Stodden model show substantial variance caused by shared environmental or genetic factors. This requires a review of studies on these traits in the behavioral genetics literature.

### 1.4 The aims of this narrative review

The first aim of this narrative review is to examine whether and how shared environmental or genetic confounding had been considered, and possibly ruled out, in the large body of literature on the five pathways in the Stodden model. To do so, we inspected all systematic reviews and meta-analysis of primary studies published after 2008 and searched for discussions on potential confounding by genetic and shared environmental factors.

As our second aim, we compare the strength of the evidence and effect sizes obtained for the effect of motor competence on physical activity in cross-sectional and longitudinal designs. This path of the Stodden model is important for intervention studies aiming to increase middle and late childhood physical activity. If familial confounding is present in this main Stodden pathway, we expect cross-sectional associations to be stronger than longitudinal associations. In addition, we expect that interventions on motor competence would not increase physical activity to the degree predicted from the cross-sectional effect sizes.

As a third aim, we explicitly test the potential for familial confounding in the five bidirectional pathways of the Stodden model. This requires that the variance in the traits in the Stodden model in childhood and adolescence are caused by genetic and shared environmental factors. This can be tested in a nuclear family design (e.g., parental, spousal and sibling correlations) or in a wider pedigrees (correlations between, e.g., self-aunt/uncle, self-niece/nephew, etc.), but the strongest design focuses on the comparison of MZ and DZ twin correlations (Knopik et al., 2017; Polderman et al., 2015). We, therefore, review the existing twin studies on the contribution of genetic and shared environmental factors to each of the traits in the Stodden model. Furthermore, we review direct tests of familial confounding that estimate the overlap in genetic and shared environmental factors influencing multiple traits, e.g., between motor competence and physical activity.

In short, our research questions are:

1. To what extent have past systematic reviews and meta-analyses on the pathways of the Stodden model considered unmeasured confounding, in by particular genetic and shared environmental factors?
2. In the main pathway between motor competence and physical activity, are the reported cross-sectional associations stronger than longitudinal associations that in turn are stronger than the effects seen in intervention studies?
3. Do twin studies show that, during childhood and adolescence, genetic and shared environmental factors contribute to individual differences in the traits used in the Stodden model?

## 2 Method

### 2.1 Search and selection of systematic reviews and meta-analyses on the Stodden model

To address research question 1, a literature search was performed for systematic reviews and/or meta-analyses of the relationships between motor competence, physical activity, perceived motor competence, and physical fitness. Definitions and assessment strategies for these traits can be found in the Supplementary Sections 1, 2. We searched the Pubmed, Web of Science, and EMBASE databases for reviews published after January 1, 2009 (i.e., after the publication of the Stodden model) and before January 15, 2025 (date of final search). The detailed search strategy is shown in Supplementary Methods Section 3’. Of note, for physical activity traits we only extracted results on total physical activity (TPA), moderate-to-vigorous physical activity (MVPA) or leisure time physical activities (LTPA, including exercise and sports) but discarded light physical activity and sedentary behavior.

The extracted titles and abstracts were initially screened by YZ to identify reports fulfilling the inclusion criteria. Articles were stored in the Endnote citation manager. Full-text reading of selected systematic reviews and meta-analyses was performed independently by YZ and EdG. Discrepancies in article selection were discussed and resolved. References were checked to identify additional systematic reviews and meta-analyses on the traits in the Stodden model.

#### 2.1.1 Inclusion and exclusion criteria

**Figure 2:**
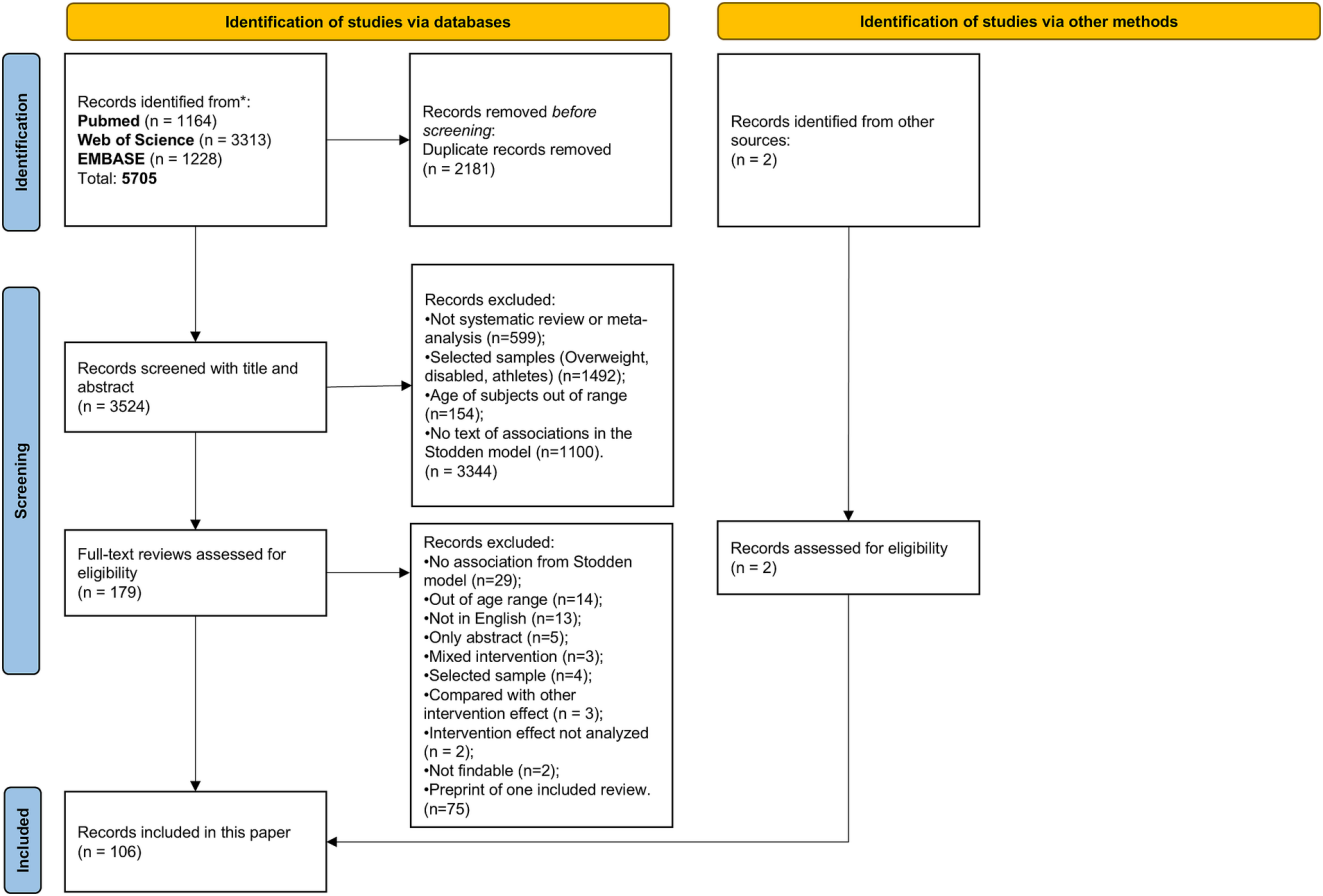
Flow diagram describing the selection of the systematic reviews and meta-analyses on the core pathways of the Stodden model.

We included peer-reviewed systematic reviews or meta-analyses of observational or interventional studies in humans that assessed the association between any two of the four traits. Only reviews published in English with a focus on participants younger than 18 years old were included. We excluded reviews where the traits from the Stodden model were not among the primary outcomes, or were no association statistics or intervention effects were reported. We excluded reviews on special populations such as youth athletes, children with medical problems or psychiatric conditions. Figure 2 provides a flow diagram describing the selection of the reviews included in the data extraction and analysis step.

#### 2.1.2 Data extraction

We extracted the authors of the systematic reviews, year of publication, presence of a meta-analysis, period covered by the search used, age range of the target population, number of primary studies included in the review, and the study design of the primary studies, i.e., (cluster) randomized controlled trial, non-randomized interventional studies, longitudinal, or cross-sectional studies (see Table 1). We then scrutinized the text of the discussion and conclusion sections of the reviews for mention of concerns about unmeasured confounding in general, and more specifically about genetic and shared environmental confounding. This process was repeated by both authors, and an automated text search for the keywords ‘risk of bias’, ‘confound*’, ‘familial’, ‘environment*’, ‘genetic*’ and ‘heritab*’ was used to verify our manual inspection. All information was extracted separately for all pathways (e.g., motor competence and physical activity, perceived motor competence and actual motor competence, motor competence and fitness, etc.) and ordered by age within each pathway.

**Table 1:** Systematic reviews and meta-analyses on the core pathways in the Stodden model (2008).

No.	Review	Type of review	Age range (years)	Trait 1	Trait 2	Primary studies on trait 1 and 2 (RCT/ INT/ LON/ CSS)	Confounding mentioned		
							General	Environ mental effects	Genetic effects
MC and PA (44 reviews included)									
1	Øglund et al. (2015)	Systematic review and meta-analysis	0–2	Motor development	PA	3 (0/0/2/1) *N* = 13,534	√	√	X
2	Santos et al. (2023)	Systematic review	0–3	MC	PA (aquatic activities)	6 (0/3/2/3) *N* = 215	√	X	X
3	Carson et al. (2017)	Systematic review	0–4	PA	Motor development	22 (6/6/1/10) *N* = 5,380	√	X	X
4	Timmons et al. (2012)	Systematic review	0–4	PA	MC	4 (3/1/0/0) *N* = 802	√	X	X
5	Bingham et al. (2016)	Systematic review	0–6	MC	TPA	11 (0/0/1/10) *N* = 12,338	√	X	X
6	Hesketh et al. (2017)	Systematic review	0–6	Motor skills	PA	10 (7/3/0/0) *N* = 3,204	X	X	X
7	Grady et al. (2025)	Systematic review and meta-analysis	0–6	PA (school and care-based PA)	FMS	16 (16/0/0/0) *N* = 4,905	√	X	X
8	Behringer et al. (2011)	Meta-analysis	0–18	PA (strength training)	Jump, run, throw	34 (0/34/0/0) *N* = 1,432	X	X	X
9	Chen et al. (2024)	Meta-analysis	2–6	PA	FMS	23 (22/1/0/0) *N* = 4,068	√	X	X
10	Barnett et al. (2022)	Systematic review	2–18	MC	PA	30 (2/0/26/3) *N* = 15,900	√	√	√
11	Figueroa and An (2017)	Systematic review	3–5	Motor skills	PA	11 (6/0//5) *N* = 2,157	√	X	X
12	Engel et al. (2018)	Systematic review and meta-analysis	3–5	FMS	TPA	11 (8/3/0/0) *N* = 3,023	√	X	X
13	Barnett et al. (2016)	Systematic review and meta-analysis	3–5	Locomotor skills	PA	13 (1/0/4/8) *N* = 6,556	√	√	X
14	Van Capelle et al. (2017)	Systematic review and meta-analysis	3–5	PA	MC	20 (11/9/0/0) *N* = 4,245	√	X	X
15	Veldman et al. (2021)	Systematic review	3–5	MVPA	Motor development	11 (4/6/1/1) *N* = 1,341	√	X	X
16	Xin et al. (2020)	Systematic review	3–6	FMS	PA	26 (0/0/2/24) *N* = 4,851	√	√	X
17	Xu et al. (2024)	Systematic review	3–6	FMS	MVPA	21 (0/0/4/18) *N* = 26,275	X	X	X
18	Jones et al. (2020)	Systematic review and meta-analysis	3–6	FMS	MVPA	19 (0/0/5/15) *N* = 3,690	X	X	X
19	Liu Y. et al. (2023)	Systematic review and meta-analysis	3–6	FMS	MVPA	11 (0/0/3/8) *N* = 2,514	√	X	X
20	Wang and Zhou (2024)	Meta-analysis	3–6	PA (MC-focused)	Gross motor skills	23 (23/0/0/0) *N* = 2070	√	X	X
21	Li et al. (2022)	Systematic review and meta-analysis	3–7	PA (extra PE)	FMS	23 (17/6/0/0) *N* = 2,258	√	X	X
22	Sinclair and Roscoe (2023)	Systematic review	3–11	PA (swimming)	FMS	10 (3/7/0/0) *N* = 611	√	X	X
23	Johnstone et al. (2018)	Systematic review and meta-analysis	3–12	PA (active play)	FMS	2 (2/0/0/0) *N* = 193	√	X	X
24	Liu et al. (2020)	Systematic review	3–12	PA (active video games)	FMS	9 (6/3/0/0) *N* = 478	√	X	X
25	Hassan et al. (2022)	Systematic review and network meta-analysis	3–12	PA (aerobic exercise)	Gross motor skills	13 (13/0/0/0) *N* = 1,109	√	X	X
26	Oppici et al. (2022)	Systematic review and meta-analysis	3–12	PA (exergame)	FMS	9 (6/3/0/0) *N* = 783	√	X	X
27	Sun and Chen (2024)	Systematic review and meta-analysis	3–12	PA (sports)	FMS	12 (12/0/0/0) *N* = 1701	√	X	X
28	Zhang et al. (2024)	Systematic review	8–17	PA	FMS	26 (11/15/0/0) *N* = 1,133	X	X	X
29	Lorås (2020)	Systematic review and meta-analysis	3–13	PA (extra PE)	MC	20 (10/10/0/0) *N* = 4,190	√	X	X
30	Holfelder and Schott (2014)	Systematic review	3–18	FMS	PA	22 (0/0/4/18) *N* = 10,107	√	X	X
31	Logan et al. (2015)	Systematic review	3–18	FMS	PA	13 (0/0/1/12) *N* = 10,534	X	X	X
32	Lubans et al. (2010)	Systematic review	3–18	FMS	PA	18 (0/0/4/14) *N* = 8,981	X	X	X
33	García-Hermoso et al. (2020)	Systematic review and meta-analysis	3–18	PA (extra PE)	FMS	15 (11/4/0/0) *N* = 7,177	√	X	X
34	Zeng et al. (2017)	Systematic review	4–6	PA	Motor skills	10 (10/0/0/0) *N* = 1,602	√	X	X
35	Comeras-Chueca et al. (2021)	Systematic review and meta-analysis	4–15	PA (active video game)	Motor competence	10 (8/2/0/0) *N* = 979	√	X	X
36	Graham et al. (2022)	Systematic review and meta-analysis	5–11	FMS	MVPA	19 (15/4/0/0) *N* = 10,412	√	X	X
37	Moon et al. (2024)	Systematic review and meta-analysis	5–12	PA (extra PE)	MC	27 (10/17/0/0) *N* = 13,281	√	X	X
38	Norris et al. (2016)	Systematic review	5–15	PA (active video game)	Motor skills	8 (4/4/0/0) *N* = 1,063	√	X	X
39	Dudley et al. (2011)	Systematic review	5–18	PA (extra PE and school sport)	MC	4 (3/1/0/0) *N* = 3,196	√	X	X
40	Collins et al. (2019a)	Meta-analysis	5–18	Strength training	Throw, sprint, squat, and jump	20 (0/20/0/0) *N* = 1,028	√	X	X
41	McDonough et al. (2020)	Systematic review	6–12	PA (active video games)	Motor skills	25 (25/0/0/0) *N* = 4,325	√	X	X
42	Rico-González (2023)	Systematic review	6–12	PA (extra PE)	FMS	4 (4/0/0/0) *N* = 1,235	√	√	X
43	Poitras et al. (2016)	Systematic review	7–15	Motor skills	TPA	9 (1/1/1/6) *N* = 5,013	√	X	X
44	Burton et al. (2023)	Systematic review and meta-analysis	11–17	MC	PA	30 (1/0/10/19) *N* = 17,702	√	X	X
MC and PMC (4 reviews included)									
*10*	Barnett et al. (2022)	Systematic review	2–18	MC	PMC	11 (0/2/3/6) *N* = 3,187	√	√	√
*32*	Lubans et al. (2010)	Systematic review	3–18	FMS	PMC	3 (3/0/0/0) *N* = 1,288	X	X	X
45	De Meester et al. (2020)	Systematic review and meta-analysis	3–24	MC	PMC	32 (1/0/3/29) *N* = 7,959	√	X	X
*44*	Burton et al. (2023)	Systematic review and meta-analysis	11–17	MC	PMC	58 (3/0/10/45) *N* = 22,256	√	X	X
PMC and PA (5 reviews included)									
46	Wang and Zhou (2023)	Systematic review	4–12	PA (MVPA)	PMC	3 (0/0/1/2) *N* = 1,464	X	X	X
47	Craggs et al. (2011)	Systematic review	4–18	PMC	PA	8 (0/0/8/0) *N* = 2,768	X	X	X
48	Babic et al. (2014)	Systematic review and meta-analysis	5–20	PMC	PA	46 (0/2/12/34) *N* = 32,438	√	X	X
49	Zamorano-Garcia et al. (2023)	Meta-analysis	7–18	PA	Perceived sport competence	10 (1/9/0/0) *N* = 3,626	√	√	X
50	Collins et al. (2019a)	Systematic review and meta-analysis	10–16	Resistance training	Perceived sport competence	7 (2/5/0/0) *N* = 460	√	√	X
MC and Physical fitness (10 reviews included)									
*10*	Barnett et al. (2022)	Systematic review and meta-analysis	2–18	MC	Physical fitness	16 (2/0/13/1) *N* = 6,039	√	√	√
51	Hui et al. (2024)	Systematic review and meta-analysis	3–10	MC	Physical fitness	23 (0/23/0/0) *N* = 2007	√	√	X
52	Liu C. et al. (2023)	Systematic review	3–16	FMS	CRF	16 (0/0/1/15) *N* = 14,336	X	X	X
*32*	Lubans et al. (2010)	Systematic review	3–18	FMS	Physical fitness	18 (0/0/4/14) *N* = 8,981	X	X	X
53	Cattuzzo et al. (2016)	Systematic review	3–18	MC	CRF	38 (0/0/7/31) *N* = 35,189	X	√	X
54	Utesch et al. (2019)	Meta-analysis	4–20	MC	CRF	19 (0/0/0/19) *N* = 15,984	X	X	X
55	Lang et al. (2018)	Systematic review	5–17	MC	CRF	4 (0/0/0/4) *N* = 2,670	√	√	X
56	Lin et al. (2022)	Systematic review	6–10	Neuromuscular training	Physical fitness	4 (0/4/0/0) *N* = 346	X	X	X
57	Jiang et al. (2024)	Systematic review and meta-analysis	7–14	MC	CRF	2 (0/0/0/2) *N* = 4,932	√	√	X
*44*	Burton et al. (2023)	Systematic review and meta-analysis	11–17	MC	Physical fitness	7 (1/0/1/5) *N* = 1,146	√	X	X
Physical fitness and PA (54 reviews included)									
58	Henriques-Neto et al. (2020)	Systematic review	0–18	Commuting PA	CRF and muscular strength	11 (1/1/1/8) *N* = 18,592	√	√	X
59	Smith et al. (2019)	Systematic review and meta-analysis	1–25	PA (extra PE)	Muscular fitness	17 (16/1/0/0) *N* = 1,653	√	X	X
60	Garcia-Hermoso et al. (2020)	Systematic review and meta-analysis	3–6	PA	CRF	9 (9/0/0/0) *N* = 4,006	√	X	X
61	Szeszulski et al. (2019)	Systematic review and meta-analysis	3–7	PA (school and care-based PA)	CRF	10 (8/2/0/0) *N* = 3,061	X	√	X
*24*	Liu et al. (2020)	Systematic review	3–12	PA (active video games)	Physical fitness	5 (5/0/0/0) *N* = 304	√	X	X
62	Pozuelo-Carrascosa et al. (2018)	Meta-analysis	3–12	PA (extra PE)	CRF	20 (20/0/0/0) *N* = 7,287	√	X	X
63	Stojanović et al. (2024)	Systematic review and meta-analysis	3–18	PA	CRF	3 (0/0/0/3) *N* = 605	X	X	X
64	Garcia-Hermoso et al. (2021)	Systematic review and meta-analysis	3–18	VPA	CRF	4 (0/0/4/0) *N* = 565	√	X	X
*33*	García-Hermoso et al. (2020)	Systematic review and meta-analysis	3–18	PA (extra PE)	CRF	20 (17/3/0/0) *N* = 4,485	√	X	X
65	Breslin et al. (2023)	Systematic review	4–12	PA (The Daily Mile)	Physical fitness	9 (1/8/0/0) *N* = 5,581	X	X	X
66	Anico et al. (2022)	Systematic review	4–12	School-based run/walk	CRF	7 (0/5/2/0) *N* = 5,024	√	√	X
67	Gutierrez-Garcia et al. (2018)	Systematic review	4–14	PA (Judo)	Physical fitness	4 (0/4/0/0) *N* = 403	X	X	X
*35*	Comeras-Chueca et al. (2021)	Systematic review and meta-analysis	4–15	Active video game	CRF	6 (3/3/0/0) *N* = 1,005	√	X	X
68	Villa-González et al. (2023)	Systematic review and meta-analysis	5–13	PA (extra PE)	Muscular fitness	17 (16/1/0/0) *N* = 1,653	√	√	X
69	Duncombe et al. (2022)	Systematic review and meta-analysis	5–17	HIIT	CRF	30 (24/6/0/0) *N* = 3,026	√	X	X
70	Larouche et al. (2014)	Systematic review	5–17	Commuting PA	CRF	10 (0/0/2/8) *N* = 26,948	√	X	X
71	Wu et al. (2023)	Systematic review and network meta-analysis	5–18	PA (extra PE)	Physical fitness	63 (48/15/0/0) *N* = 7,226	X	√	X
72	Zhou et al. (2024)	Systematic review	5–18	PA	Physical fitness	30 (24/7/0/0) *N* = 6,494	X	X	X
73	Bauer et al. (2022)	Systematic review and meta-analysis	5–18	HIIT	CRF	8 (0/8/0/0) *N* = 867	X	X	X
74	Eather et al. (2022)	Systematic review and meta-analysis	5–18	HIIT	CRF and muscular fitness	11 (3/8/0/0) *N* = 1,011	X	X	X
75	Lubans et al. (2011)	Systematic review	5–18	Commuting PA	CRF	5 (0/0/1/4) *N* = 13,604	√	√	X
76	Sun et al. (2013)	Systematic review	5–18	PA (extra PE)	CRF	11 (11/0/0/0) *N* = 2,694	√	√	X
77	Wu et al. (2021)	Meta-analysis	5–18	Resistance training	Muscle strength	42 (42/0/0/0) *N* = 1728	X	X	X
78	Moran et al. (2018)	Systematic review and meta-analysis	5–18	PA	Muscular fitness	21 (21/0/0/0) *N* = 2,267	X	X	X
79	Hanna et al. (2023)	Systematic review	6–11	PA (The Daily Mile)	Physical fitness	5 (0/5/0/0) *N* = 2,700	X	√	X
80	Errisuriz et al. (2018)	Systematic review	6–11	PA (extra PE)	CRF	8 (4/4/0/0) *N* = 12,977	X	X	X
*42*	Rico-González (2023)	Systematic review	6–12	PA (extra PE)	Physical fitness	8 (8/0/0/0) *N* = 5,710	√	√	X
81	Beets et al. (2009)	Meta-analysis	6–12	PA (after-school program)	Physical fitness	6 (6/1/0/0) *N* = 4,686	X	X	X
82	Burns et al. (2018)	Meta-analysis	6–12	PA	CRF	20 (13/10/0) *N* = 10,779	X	X	X
83	Braaksma et al. (2018)	Systematic review	6–12	PA	CRF	23 (23/0/0/0) *N* = 7,071	X	X	X
84	Reyes-Amigo et al. (2017)	Systematic review	6–12	HIIT	CRF	10 (6/4/0/0) *N* = 330	X	X	X
85	Gäbler et al. (2018)	Systematic review and meta-analysis	6–18	PA	Physical fitness	15 (0/15/0/0) *N* = 595	X	X	X
86	Neil-Sztramko et al. (2021)	Systematic review and meta-analysis	6–18	PA (extra PE)	Physical fitness	41 (41/0/0/0) *N* = NR	X	√	X
87	Li et al. (2024)	Multivariate and Network Meta-analysis	6–18	PA	Physical fitness	36 (0/0/NR/NR) *N* = 2,658	√	X	X
88	Gralla et al. (2019)	Systematic review	6–18	VPA	CRF	16 (0/0/0/16) *N* = 8,041	X	X	X
89	Cibinello et al. (2023)	Systematic review and meta-analysis	6–18	Pilates	Flexibility and muscle strength	10 (0/0/NR/NR) *N* = 804	√	√	X
90	Behringer et al. (2010)	Systematic review	6–18	PA	Muscular fitness	77 (1/1/12/63) *N* = 1728	X	X	X
*43*	Poitras et al. (2016)	Systematic review	7–16	PA	CRF	38 (6/3/1/28) *N* = 26,865	√	X	X
91	Lei and Jun (2022)	Systematic review	7–17	PA (Taekwondo Poomsae training)	Physical fitness	15 (0/15/0/0) *N* = 536	X	X	X
92	Pinho et al. (2024)	Meta-analysis	7–17	PA	Physical fitness	80 (0/0/NR/NR) *N* = 5,769	X	X	X
93	Clemente et al. (2022)	Systematic review	7–18	PA (soccer training)	Physical fitness	13 (0/13/0/0) *N* = 2,794	√	X	X
94	Woodforde et al. (2022)	Systematic review	7–18	PA (extra PE)	Physical fitness	4 (2/2/0/0) *N* = 444	X	X	X
95	Cox et al. (2020)	Meta-analysis	8–18	Resistance training	Muscle strength	11 (0/0/NR/NR) *N* = 253	√	√	X
96	Peralta et al. (2020)	Systematic review	8–19	PA (extra PE)	CRF	24 (0/15/2/7) *N* = 15,159	X	X	X
97	Zhao et al. (2023)	Meta-analysis	10–12	PA (jumping rope)	Physical fitness	15 (15/0/0/0) *N* = 1,048	√	√	X
98	Ferreira et al. (2024)	Systematic review	10–15	PA (swim exercise)	Physical fitness	5 (0/5/0/0) *N* = 459	√	X	X
99	Ramirez-Campillo et al. (2023)	Systematic review and meta-analysis	10–16	PA (Plyometric training)	Physical fitness	11 (0/0/??/??) *N*=NR	X	X	X
100	Garcia-Banos et al. (2020)	Systematic review and meta-analysis	10–18	PA (extra PE)	Muscular fitness	11 (3/8/0/0) *N* = 1,161	X	X	X
101	Minatto et al. (2016)	Systematic review and meta-analysis	10–19	PA (extra PE)	CRF	40 (23/17/0/0) *N* = 19,970	√	√	X
102	da Silva Bento et al. (2022)	Systematic review	10–19	HIIT	CRF	14 (14/0/0/0) *N* = 664	X	X	X
103	de Andrade Gonçalves et al. (2015)	Systematic review	11–19	PA	Physical fitness	6 (0/0/1/5) *N* = 7,599	X	√	X
104	Singh et al. (2022)	Systematic and meta-analysis	11–19	PA (jump rope training)	CRF	13 (2/11/0/0) *N* = 538	X	X	X
105	Costigan et al. (2015)	Systematic review and meta-analysis	13–18	HIIT	CRF	15 (9/6/0/0) *N* = 1,110	X	X	X
106	Behm et al. (2017)	Systematic review	13–18	PA (extra PE)	Muscular strength	8 (0/0/NR/NR) *N* = 3,297	X	X	X

CRF = Cardiorespiratory fitness; CSS = Cross-sectional Studies; FMS = Fundamental Movement Skills; HIIT = High-intensity interval training; INT = Non-randomized intervention studies; LON = Longitudinal studies; MC = Motor competence; MVPA = Moderate-to-vigorous physical activity; NR = Not reported; PA = Physical activity; PE = Physical Education; RCT = (Clustered) Randomized Controlled Trials studies; PMC = Perceived motor competence; RCT = (Clustered) Randomized Controlled Trial studies; TPA = total physical activity; VPA = Vigorous physical activity.

#### 2.1.3 Strength of evidence and effect sizes in the motor competence—physical activity pathway

To address research question 2, we extracted additional data on the overall strength of evidence and average effect sizes reported by the included reviews on the main bidirectional pathway of the Sodden model between motor competence and physical activity (see Table 2). The information was separately provided per study design, ordered by age groups (early childhood ~2–5 years of age; middle childhood ~6–12 years of age; and adolescence ~13–18 years of age), and further by the subdomains of the traits (e.g., for motor competence, subdomains like object control skills or balancing skills).

**Table 2:** Design characteristics and main findings from the reviews on the association between motor competence and physical activity.

Review	Type of review	Publication year (search period)	Age range (years)	Trait 1 (MC domain)	Trait 2 (PA domain)	# of studies on trait 1 and 2 (RCT/INT/LON/CSS)	Overall findings	Strength of evidence*	Effect size **
Cross-sectional–early childhood									
Santos et al. (2023)	Systematic review	2023 (until 2022.12.22)	0–3	MC	PA (aquatic activities)	6 (0/3/2/3) *N* = 215	All six studies (100%) found a significant association between swimming activities and motor development.	+	Not specified
Bingham et al. (2016)	Systematic review	2016 (until 2016.09)	0–6	MC	TPA	9 (0/0/1/8) *N* = 1,202	Nine out of 23 analyses (37%) identified motor competence as associated with total physical activity.	?	Not specified
Bingham et al. (2016)	Systematic review	2016 (until 2016.09)	0–6	MC	MVPA	10 (0/0/1/9) *N* = 1809	Eleven out of 26 analyses (42%) identified motor competence as associated with moderate-to-vigorous physical activity.	?	Not specified
Figueroa and An (2017)	Systematic review	2017 (until 2015.03.31)	3–5	MC (Motor skill competence)	PA	11 (6/0/0/5) *N* = 2,157	Eight out of 11 studies (72.7%) reported a significant association. The effect size was not specified but noted to differ by gender, physical activity intensity, motor skill type, and day of the week (weekdays versus weekends).	+	Not specified
Xin et al. (2020)	Systematic review	2020 (2000.01–2020.04)	3–6	MC (fundamental movement skills)	PA	26 (0/0/2/24) *N* = 4,851	Sixteen out of 26 studies (61.5%) reported a significant association between MC and any PA trait (r = 0.10–0.46).	+	Small to moderate
Xu et al. (2024)	Systematic review	2024 (until 2022.07)	3–6	MC (fundamental movement skills)	MVPA	19 (0/0/4/18) *N* = 26,275	Across 19 studies, fifteen out of 23 (82.6%) analyses found a significant association between FMS and MVPA (r = 0.25, 95%CI: 0.22 ∼ 0.27).	+	Small
Xu et al. (2024)	Systematic review	2024 (until 2022.07)	3–6	MC (fundamental movement skills)	MVPA	12 (0/0/2/11) *N* = 6,561	Across 12 studies, seven out of 14 (50%) analyses found a significant association between FMS and TPA (r = 0.23, 95%CI: 0.19 ∼ 0.27).	?	Small
Jones et al. (2020)	Systematic review and meta-analysis	2020 (until 2019.04)	3–6	MC (fundamental motor skills)	MVPA	12 (0/0/0/12) *N* = 2,578	Eight of 12 analyses (67%) reported a significant association. Meta-analysis showed a small effect (r = 0.20, 95%CI: 0.13–0.26). Heterogeneity: *τ* value of ±0.089 from a random effect model.	+	Small
Liu Y. et al. (2023)	Systematic review and meta-analysis	2023 (until 2023.08)	3–6	MC (fundamental motor skills)	MVPA	3 (0/0/1/2) *N* = 260	Three datasets examined the association between total MC and MVPA. Meta-analysis showed a large effect (*β* = 0.56, 95% CI: 0.38–0.75, *p* = 0.001). No heterogeneity (*I*^2^ = 0%, *p* = 0.99) from a random effect test.	?	Large
Xin et al. (2020)	Systematic review	2020 (2000.01–2020.04)	3–6	MC (fundamental movement skills)	MVPA	16 (0/0/1/16) *N* = 2,617	Eleven out of 16 studies (69%) found a significant association between MC and MVPA.	+	Small to moderate
Jones et al. (2020)	Systematic review and meta-analysis	2020 (until 2019.04)	3–6	MC (fundamental motor skills)	TPA	12 (0/0/0/12) *N* = 1903	Ten out of 12 analyses (83%) found a significant association. Meta-analysis showed a small effect (r = 0.20, 95%CI: 0.12–0.28). Heterogeneity: τ value of ±0.113 from a random effect model.	+	Small
Xin et al. (2020)	Systematic review	2020 (2000.01–2020.04)	3–6	MC (fundamental movement skills)	TPA	12 (0/0/0/12) *N* = 2,152	Nine out of 12 studies (75%) supported small to moderate associations between MC and TPA.	+	Small to moderate
Barnett et al. (2016)	Systematic review and meta-analysis	2016 (1995–2014)	3–5	Locomotor skills	PA/Sports	7 (1/0/0/6) *N* = 963	Five out of 11 analyses (45%) found a significant association between PA/sports and locomotor skills. Heterogeneity was not reported on this association.	?	Not specified
Xin et al. (2020)	Systematic review	2020 (2000.01–2020.04)	3–6	Locomotor skills	TPA	10 (0/0/0/10) *N* = 2,144	Six out of 10 studies (60%) found a significant association between locomotor skills and TPA.	+	Small to moderate
Xin et al. (2020)	Systematic review	2020 (2000.01–2020.04)	3–6	Locomotor skills	MVPA	16 (0/0/2/15) *N* = 3,024	Nine out of 16 studies (56%) found a significant association between locomotor skills and MVPA.	?	Small to moderate
Liu Y. et al. (2023)	Systematic review and meta-analysis	2023 (until 2023.08)	3–6	Locomotor skills	MVPA	5 (0/0/1/4) *N* = 981	Six datasets examined the association between locomotor skill and MVPA Meta-analysis showed no association (β = 0.06, 95% CI: −0.35- 0.47, *p* = 0.79). Heterogeneity: *I*^2^ = 90.26%, (*p* = 0.001) from a random effect test.	0	No association
Barnett et al. (2016)	Systematic review and meta-analysis	2016 (1995–2014)	3–5	Object control skills	PA/Sports	6 (1/0/0/5) *N* = 863	Five out of 11 analyses (45%) found a significant association between PA/sports and object control skills. Heterogeneity was not reported on this association.	?	Not specified
Xin et al. (2020)	Systematic review	2020 (2000.01–2020.04)	3–6	Object control skills	TPA	11 (0/0/0/11) *N* = 2,190	Nine out of 11 studies (82%) found a significant association between objective control skills and total physical activity.	+	Small to moderate
Xin et al. (2020)	Systematic review	2020 (2000.01–2020.04)	3–6	Object control skills	MVPA	17 (0/0/1/16) *N* = 3,024	Twelve out of 17 studies (71%) found a significant association between object control skills and MVPA.	+	Small to moderate
Liu Y. et al. (2023)	Systematic review and meta-analysis	2023 (until 2023.08)	3–6	Object control skills	MVPA	4 (0/0/1/3) *N* = 855	Four datasets examined the association between object control skill and MVPA. Meta-analysis showed a significant, small effect (*β* = 0.15, 95% CI: 0.02, 0.27, *p* = 0.02). No heterogeneity (*p* = 0.15) from a random effect test.	+	Small
Xin et al. (2020)	Systematic review	2020 (2000.01–2020.04)	3–6	Stability	TPA	4 (0/0/0/4) *N* = 1,424	Two out of four studies (50%) found a significant association between stability skills and TPA.	?	Small to moderate
Xin et al. (2020)	Systematic review	2020 (2000.01–2020.04)	3–6	Stability	MVPA	3 (0/0/1/2) *N* = 1,410	One out of three studies reported a significant association between stability skills and MVPA.	?	Small to moderate
Cross-sectional–middle/late childhood									
Holfelder and Schott (2014)	Systematic review	2014 (2000–2013.06)	3–18	MC (fundamental movement skills)	PA	12 (0/0/2/10) *N* = 6,071	Ten out of 12 studies (83.3%) found a significant association between MC and PA, with *r* ranging from 0.17 to 0.47.	+	Small to moderate
Barnett et al. (2016)	Systematic review and meta-analysis	2016 (1995–2014)	3–18	MC (skill composite)	PA/ sports	3 (1/0/1/1) *N* = 913	Three out of four analyses (75%) found a significant association between PA and motor skill composite score. Heterogeneity was not reported for the random effect test on this association.	+	Not specified
Logan et al. (2015)	Systematic review	2015 (until 2013.11)	3–18	MC (fundamental movement skills)	PA	13 (0/0/1/12) *N* = 10,534	All studies found at least one significant association between FMS and physical activity. Effect sizes differed across age: small to moderate in early childhood and adolescence and small to large in middle to late childhood.	+	Small to moderate Small to large
Lubans et al. (2010)	Systematic review	2010 (until 2009.06)	3–18	MC (fundamental movement skills)	PA	13 (0/0/2/11) *N* = 5,187	Twelve out of 13 studies (92.3%) found a significant association between MC and at least one domain of PA.	+	Not specified
Poitras et al. (2016)	Systematic review	2016 (until 2015.01)	7–15	MC (motor skill development)	TPA	6 (0/0/1/5) *N* = 5,179	Three out of five (60%) cross-sectional studies found a significant association.	+	Not specified
Burton et al. (2023)	Systematic review and meta-analysis	2023 (until 2022.08.05)	11–17	MC (motor competence)	PA	8 (1/0/0/7) *N* = 5,224	Eight out of 13 analyses (61%) found a significant association. Meta-analysis showed a small effect (r = 0.21, 95%CI: 0.12–0.30). Very high heterogeneity (*I*^2^ = 90.64) from a random effect test.	+	Small
Holfelder and Schott (2014)	Systematic review	2014 (2000–2013.06)	3–18	Locomotor skills	PA	5 (0/0/1/4) *N* = 744	All five studies (100%) found a significant, small to moderate association between object control and PA, with *r* ranging from 0.14 to 0.46.	+	Small to moderate
Burton et al. (2023)	Systematic review and meta-analysis	2023 (until 2022.08.05)	11–17	Locomotor skills	PA	5 (0/0/2/3) *N* = 1,443	Five out of six analyses (80%) found a significant association. Meta-analysis showed a small effect (r = 0.21, 95% CI: 0.12–0.30). High heterogeneity (*I*^2^ = 62.94) from a random effect test.	+	Small
Holfelder and Schott (2014)	Systematic review	2014 (2000–2013.06)	3–18	Object control Skills	PA	6 (0/0/2/4) *N* = 1824	All six studies (100%) found a significant, association between object control and PA.	+	Small to moderate
Burton et al. (2023)	Systematic review and meta-analysis	2023 (until 2022.08.05)	11–17	Object control skills	PA	6 (0/0/2/4) *N* = 5,081	Eight out of 12 analyses (67%) found a significant association. Meta-analysis showed a small effect (r = 0.26, 95% CI: 0.18–0.33). Moderate heterogeneity (*I*^2^ = 38.58) from a random effect test.	+	Moderate
Burton et al. (2023)	Systematic review and meta-analysis	2023 (until 2022.08.05)	11–17	Stability/ balance	PA	5 (0/0/1/4) *N* = 6,369	Eight out of 11 analyses (72.7%) found a significant association. Meta-analysis showed a small effect (r = 0.20,95%CI: 0.13–0.27). Very high heterogeneity (*I*^2^ = 86.22) from a random effect test.	+	Small

Review	Type of review	Publication year (search period)	Age range (years)	Exposure	Outcome	# of studies using LON	Overall findings	Strength of evidence*	Effect size **
Longitudinal–early childhood—MC - > PA									
Øglund et al. (2015)	Systematic review and meta-analysis	2015 (until 2014.09)	0–2	MC (motor development)	PA	3 *N* = 4,951	Two of three studies (66.7%) found that motor development before age 2 predicted physical activity and sport participation in youth with a small effect size. Heterogeneity was not reported for the random effect test on this association.	+	Small
Jones et al. (2020)	Systematic review and meta-analysis	2020 (until 2019.04)	3–6	MC (fundamental motor skills)	PA	5 *N* = 1,112	Three out of five longitudinal studies (60%) showed that MC predicts PA with a small effect size (0.21 < r < 0.28). Heterogeneity was not reported for the random effect test on this association.	+	Small
Xin et al. (2020)	Systematic review	2020 (2000.01–2020.04)	3–6	MC (fundamental movement skills)	PA	2 *N* = 357	Two longitudinal studies found that MC did not predict PA.	0	No prediction
Longitudinal–middle/late childhood–MC - > PA									
Graham et al. (2022)	Systematic review and meta-analysis	2021 (until 2017.05)	5–11	MC (fundamental movement skills)	MVPA	19 *N* = 10,412	Only six out of 19 studies showing significant prediction (31.5%). Fourteen of the 19 studies were pooled into a meta-analysis. FMS had a large effect on daily MVPA (13.3 min/day, 95% CI 8.0–18.6; R^2^ = 0.89). Heterogeneity: τ value of ±7.6 (95%CI: −13 to 21) from a random effect model.	?	Large
Holfelder and Schott (2014)	Systematic review	2014 (2000–2013.06)	3–18	MC (fundamental movement skills)	PA	7 *N* = 1936	Motor skill competence at baseline significantly explained 5–18% of variance in PA at follow-up in 5 out of 7 studies (71.4%).	+	Small
Barnett et al. (2022)	Systematic review	2022 (until 2019.11.08)	2–18	MC (skill composite)	PA	7 *N* = 4,167	Across seven studies, 71% of the analyses reported a significant prediction of PA by total MC (PA ranging from LPA to VPA, with most studies using MVPA).	+	Small
Barnett et al. (2022)	Systematic review	2022 (until 2019.11.08)	2–18	Locomotor, Coordination, Stability skills	PA	15 *N* = 6,331	Across 15 studies, 42% of the analyses showed locomotor and coordination/ stability skills to predict PA (PA ranging from LPA to VPA, with most studies using MVPA).	?	Small to moderate
Longitudinal–early childhood–PA- > MC									
Jones et al. (2020)	Systematic review and meta-analysis	2020 (until 2019.04)	3–6	PA	MC (fundamental motor skills)	2 *N* = 344	Only two studies explored the longitudinal prediction of MC by PA, showing PA to predict balance and locomotor, but not (or a negative effect) for agility and object control. Heterogeneity was not reported on this association.	?	Small
Xin et al. (2020)	Systematic review	2020 (2000.01–2020.04)	3–6	PA	MC (fundamental movement skills)	2 *N* = 357	Two longitudinal studies found that PA was a significant predictor for MC (β = 0.07–0.26).	?	Small
Longitudinal–middle/late childhood–PA- > MC									
Barnett et al. (2022)	Systematic review	2022 (until 2019.11.08)	2–18	PA	MC (Motor competence)	11 *N* = 5,528 (2 trials were included)	Eleven longitudinal studies investigated the pathway from PA to any form of MC, with no evidence to support an association, with only 8% analyses significant. Both interventions did not show a significant effect.	0	No effect
Barnett et al. (2022)	Systematic review	2022 (until 2019.11.08)	2–18	PA	Locomotor, Coordination, Stability skills	11 *N* = 4,586	Across 11 studies, 24% of the analyses supported a pathway from locomotor/ coordination/ stability skills to PA (ranging from LPA to VPA, with most studies using MVPA).	0	No effect
Barnett et al. (2022)	Systematic review	2022 (until 2019.11.08)	2–18	PA	Object control skills	5 *N* = 2,200	Across five studies, 38% analyses supported a pathway from object control skills to PA (ranging from LPA to VPA, with most studies using MVPA).	?	Small

Review	Type of review	Publication year (search period)	Age range (years)	Intervention	Outcome	# of RCT/INT studies	Overall findings	Strength of evidence*	Effect size **
Intervention–early childhood–MC - > PA									
Hesketh et al. (2017)	Systematic review	2017 (until 2015.10)	0–6	MC (motor skills)	PA	10 (7/3) *N* = 3,204	Out of 10 RCT/INT studies, five (50%) reported motor skills training had a positive effect on time spent on PA.	?	Not specified
Engel et al. (2018)	Systematic review and meta-analysis	2018 (until 2017.7.20)	3–5	MC (fundamental motor skills)	TPA	7 (6/1) *N* = 1,623	Three out of seven (42.9%) studies found a significant effect of MC intervention on the total amount of PA. Meta-analyses showed a small improvement in total PA (SMD = 0.32; 95% CI: 0.09–0.54; *p* = 0.006). Very high heterogeneity (*I*^2^ = 76%, Chi^2^ *p* = 0.0004) from a random effect test.	?	Small
Engel et al. (2018)	Systematic review and meta-analysis	2018 (until 2017.7.20)	3–5	MC (fundamental motor skills)	MVPA	7 (7/0) *N* = 1,531	One out of seven (14.3%) studies found a significant effect of MC intervention on MVPA. Meta-analyses showed a small improvement in MVPA (SMD = 0.21; 95% CI: 0.01–0.40; *p* = 0.03). High heterogeneity (*I*^2^ = 63%, Chi^2^ *p* = 0.01) from a random effect test.	0	Small
Intervention–middle/late childhood–MC - > PA									
Engel et al. (2018)	Systematic review and meta-analysis	2018 (until 2017.7.20)	5–12	MC (fundamental motor skills)	TPA	3 (2/1) *N* = 414	None of three studies (0%) found a significant effect of MC intervention on the total amount of PA, while meta-analysis showed a small significant improvement in total PA (SMD = 0.23; 95% CI: 0.03–0.42; *p* = 0.02). No heterogeneity (*I*^2^ = 0%, Chi^2^ *p* = 1) from a random effect test.	0	Small
Engel et al. (2018)	Systematic review and meta-analysis	2018 (until 2017.7.20)	5–12	MC (fundamental motor skills)	MVPA	3 (0/3) *N* = 348	One out of three (33.3%) studies found a significant effect of MC intervention on MVPA. Meta-analysis showed a small significant improvement in MVPA (SMD = 0.29 95% CI: 0.08–0.51; *p* = 0.007). High heterogeneity (*I*^2^ = 63%, Chi^2^ *p* = 0.01) from a random effect test.	?	Small
Intervention–early childhood–PA - > MC									
Timmons et al. (2012)	Systematic review	2012 (until 2011.03)	0–4	PA	MC	4 (3/1) *N* = 802	All four intervention studies found that PA significantly improved MC.	+	Not specified
Carson et al. (2017)	Systematic review	2017 (until 2016.4.14)	0–4	PA	MC (motor development)	12 (6/6) *N* = 5,245	Ten out of 12 intervention studies (83.3%) found that PA improved MC.	+	Not specified
Grady et al. (2025)	Systematic review and meta-analysis	2025 (2014.09–2022.10)	0–6	PA (early childhood education and care-based PA)	MC (fundamental movement skills)	16 (16/0) *N* = 4,905	Early childhood education and care-based PA was found to significantly improve FMS (SMD = 0.544, 95%CI: 0.1–0.98, *p* = 0.015). Very high heterogeneity (*I*^2^ = 95.8%) from a random effect test.	+	Moderate
Chen et al. (2024)	Meta-analysis	2024 (until 2023.11.01)	2–6	PA (MC-focused exercise training)	MC (fundamental motor skills)	23 (22/1) *N* = 4,068	All 23 intervention studies (22 RCT) found that motor skills-focused exercise training significantly improved FMS compared to the control group, with structured intervention the most effective (Hedge’s *g* = 1.29, *p* < 0.001). No heterogeneity (*I*^2^ < 25%) from a random effect test.	+	Large
Wang and Zhou (2024)	Meta-analysis	2024 (until 2024.03)	3–6	PA (MC-focused exercise training)	MC (gross motor skills)	23 (23/0/0/0) *N* = 2070	Twenty out of 23 studies were included in the meta-analysis. Of these, 17 (85%) showed significant improvement by motor skills-focused exercise training on gross motor skills as compared to active control (Cohen’s d = 1.53). Very high heterogeneity (*I*^2^ = 93%, *p* < 0.01) from a random effect test.	+	Large
Veldman et al. (2021)	Systematic review	2021 (until 2019.11.21)	3–5	MVPA	MC (motor development)	11 (4/6) *N* = 1,281	All ten intervention studies (100%) found a positive effect of MVPA on motor development (either total score, a specific component, or an individual skill).	+	Not specified
Van Capelle et al. (2017)	Systematic review and meta-analysis	2017 (until 2016.03.30)	3–5	PA	MC (fundamental motor skills)	21 (6/15) *N* = 4,245	Restricting to teacher-led interventions, the meta-analysis across 13 analyses indicated that PA intervention led to a trivial but significant improvement in MC (SMD = 0.13, 95%CI: 0.03–0.22, *p* = 0.008) in only 4 analyses (30.7%). Very high heterogeneity (*I*^2^ = 84%, *p* < 0.00001) from a random effect test.	?	Small
Zeng et al. (2017)	Systematic review	2017 (2000.01–2017.07)	4–6	PA	MC (motor skills)	10 (10/0) *N* = 1,602	Eight out of 10 RCTs (80%) reported that increasing PA led to significant improvements in motor performance.	+	Not specified
Van Capelle et al. (2017)	Systematic review and meta-analysis	2017 (until 2016.03.30)	3–5	PA	Object control skills	7 (5/2) *N* = 558	Restricting to teacher led interventions the meta-analysis across eight analyses indicated a small but significant improvement in object control skills (SMD = 0.47, 95%CI: 0.15–0.80, *p* = 0.004) in 6 analyses (75%). Very high heterogeneity (*I*^2^ = 89%, p < 0.00001) from a random effect test.	+	Small
Van Capelle et al. (2017)	Systematic review and meta-analysis	2017 (until 2016.03.30)	3–5	PA	Locomotor skills	5 (4/1) *N* = 602	Restricting to teacher led interventions the meta-analysis across seven analyses indicated a small significant improvement in locomotor skills (SMD = 0.44, 95%CI: 0.16–0.73, *p* = 0.002). High heterogeneity (*I*^2^ = 57%, *p* = 0.06) from a random effect test.	+	Small
Li et al. (2022)	Systematic review and meta-analysis	2021	3–7	PA (physical education)	MC (fundamental movement skills)	23 (17/6) *N* = 2,258	Meta-analysis showed a significant improvement of extra physical education on any form of MC (SMD range:1.38–1.56, *I^2^* = 59.2–93.8%). Very high heterogeneity (*I*^2^ = 89.7%, *p* = 0.0000) from a random effect test.	+	Large
Johnstone et al. (2018)	Systematic review and meta-analysis	2018 (until 2016.12)	3–12	PA (active play)	MC (fundamental movement skills)	2 (2/0) *N* = 193	Both studies showed a significant effect of active play interventions on children’s FMS quotient score and one-leg balance. Meta-analysis could not be conducted with two studies.	?	Not specified
Liu et al. (2020)	Systematic review	2020 (until 2020.10)	3–12	PA (active video games)	MC (fundamental movement skills)	5 (3/2) *N* = 340	Across five studies, two (40%) reported that active video games significantly improved FMS compared to a control manipulation.	?	Not specified
Zhang et al. (2024)	Systematic review	2024 (2000–2023)	8–17	PA	MC (fundamental motor skills)	26 (11/15) *N* = 1,133	Across 26 studies, 16 out of 17 (94.1%), ten out of ten (100%), and two out of two studies (100%) found significant effect of PA on locomotor, balance, and object control skills, respectively.	+	Not specified
Oppici et al. (2022)	Systematic review and meta-analysis	2022 (2007–2022)	3–12	PA (exergaming)	MC (fundamental movement skills)	9 (6/3) *N* = 783	Across nine studies, seven out of 14 analyses (50%) found that exergaming had significant improvements in MC as compared to the control (r = 0.24, 95%CI: 0.11–0.36). Heterogeneity from a random effect test was not reported.	?	Small
Sun and Chen (2024)	Systematic review and meta-analysis	2024 (2001–2022)	3–12	PA (sports game)	MC (fundamental motor skills)	12 (12/0) *N* = 1701	All 12 studies (100%) found that sports game interventions had a significant effect on MC (SMD = 0.30, *p* < 0.0001). No details on the meta-analytic test approach were reported.	+	Small
Intervention–Middle/Late Childhood–PA - > MC									
Moon et al. (2024)	Systematic review and meta-analysis	2024 (until 2021.11)	5–12	PA (physical education)	MC	27 (10/17) *N* = 13,281	Twenty-six studies were included in meta-analyses with 22 (84.6%) showing statistically significant effect on MC (Hedges’ g = 0.71; 95% CI = 0.60–0.81; *p* < 0.001). Very high heterogeneity (*I*^2^ = 78.4%) from a random effect test.	+	Large
Rico-González (2023)	Systematic review	2023 (until 2022.02)	4–12	PA (school-based physical education)	FMS	4 (4/0)	Across four studies, three (75%) showed significant effects of PA (school-based physical education) on FMS.	+	Not specified
Norris et al. (2016)	Systematic review	2016 (until 2015.05)	5–15	PA (active video game)	MC (motor skills)	3 (2/1) *N* = 805	Across three studies, two (66.7%) showed significant effects of PA (active video game) on MC as compared to control.	+	Not specified
García-Hermoso et al. (2020)	Systematic review and meta-analysis	2020 (until 2019.10)	3–18	PA (physical education)	FMS	7 (5/2) *N* = 3,870	All seven studies (100%) showed significant effects of PE-based PA interventions on FMS (Hedges g = 0.38; 95% CI, 0.27–0.49). High heterogeneity (*I*^2^ = 73.4%, *p* = 0.02) from a random effect test.	+	Small
Dudley et al. (2011)	Systematic review	2011 (1990.01–2010.06)	5–18	PE and school sport	MC	4 (3/1) *N* = 3,196	Across four studies, all analyses supported an effect of school sports on MC.	+	Not specified
Sinclair and Roscoe (2023)	Systematic review	2023 (until 2023.02)	3–11	PA (swimming)	MC (fundamental movement skills)	10 (3/7/0/0) *N* = 611	All ten studies found that swimming significantly improved at least one domain of MC.	+	Not specified
Hassan et al. (2022)	Systematic review and network meta-analysis	2022 (Until 2022.05)	3–12	Aerobic exercise	MC (gross motor skills)	13 (13/0) *N* = 1,109	Network meta-analysis showed that aerobic exercise training was an effective treatment for the total gross motor skills (ES: 7.49, 95% Cl: 0.1 to 15.7). Bayesian random-effects modeling was used and no heterogeneity was found.	+	Large
Hassan et al. (2022)	Systematic review and network meta-analysis	2022 (Until 2022.05)	3–12	PA (exergaming)	MC (gross motor skills)	13 (13/0) *N* = 1,109	Network meta-analysis showed that exergaming was not an effective treatment for the total gross motor skills (ES: −0.17, 95% Cl: −12.8 to 12.4). Bayesian random-effects modeling was used and no heterogeneity was found.	0	No effect
Hassan et al. (2022)	Systematic review and network meta-analysis	2022 (Until 2022.05)	3–12	Aerobic exercise training	Locomotor skills	11 (11/0) *N* = 1,021	Network meta-analysis showed that aerobic exercise training was not an effective treatment for locomotor skills (ES: 4.12, 95% Cl: −1.4 to 9.4). Bayesian random-effects modeling was used and no heterogeneity was found.	0	No effect
Hassan et al. (2022)	Systematic review and network meta-analysis	2022 (Until 2022.05)	3–12	PA (exergaming)	Locomotor skills	11 (11/0) *N* = 1,021	Network meta-analysis showed that exergaming was an effective treatment for locomotor skills (ES: 12.50, 95% Crl: 0.28 to 24.50). Bayesian random-effects modeling was used and no heterogeneity was found.	+	Large
Hassan et al. (2022)	Systematic review and network meta-analysis	2022 (Until 2022.05)	3–12	Aerobic exercise training	Object control skills	16 (16/0) *N* = 1,515	Network meta-analysis showed that aerobic exercise training was an effective treatment for object control skills (SMD: 6.90, 95% Cl: 1.39 to 13.50). Bayesian random-effects modeling was used and no heterogeneity was found.	+	Large
Hassan et al. (2022)	Systematic review and network meta-analysis	2022 (Until 2022.05)	3–12	PA (exergaming)	Object control skills	16 (16/0) *N* = 1,515	Network meta-analysis showed that exergaming was not an effective treatment for object control skills (SMD: −0.4, 95% Cl: −10.2 to 8.9). Bayesian random-effects modeling was used and no heterogeneity was found.	0	No effect
McDonough et al. (2020)	Systematic review	2020 (2000–2020)	6–12	PA (18), exergaming (7)	MC (motor skill development)	25 (25/0) *N* = 4,325	Out of 25 RCTs, 20 (80%) that used various PA interventions led to significant improvements children’s motor skill development.	+	Not specified
Lorås (2020)	Systematic review and meta-analysis	2020 (2002–2020)	3–13	PA (physical education)	MC	20 (10/10/0/0) *N* = 4,190	Sixteen out of 23 analyses (69.6%) found that physical education intervention had significant effect on overall motor competence (*g* = −0.69, 95%CI: −0.91 to-0.46, *p* < 0.001). Very high heterogeneity (*I*^2^ = 92.74%) from a random effect test.	+	Moderate
Carson et al. (2017)	Systematic review	2016 (until 2015.01)	7–15	PA	MC (motor skill development)	2 (1/1) *N* = 203	Neither of the two intervention studies found effects on motor skill development.	?	No effect
Behringer et al. (2011)	Meta-analysis	2011 (until 2009.08)	0–18	Strength training	Jump, run, throw	34 (0/34) *N* = 1,432	Meta-analysis indicated that strength training led to a significant improvement in combination of jumping, running, and throwing ES = 0.52 (95%CI: 0.33–0.71). No heterogeneity (*I*^2^ = 0%) from a fixed effects model.	+	Moderate to large
Comeras-Chueca et al. (2021)	Systematic review and meta-analysis	2021 (until 2021.03)	4–15	Active video game	Motor competence	10 (8/2) *N* = 979	Seven out of 10 (70%) studies found that active video game had a significant improvement on motor competence. Heterogeneity wasn’t reported for the random effect test on the PA-MC association.	+	Not specified
Collins et al. (2019a)	Meta-analysis	2019 (until 2017.06)	5–18	Strength training	Throw, sprint, squat-, standing-long-, and vertical jump	22 (0/22) *N* = 943	Significant intervention effects were identified in 33 analyses on sprint (Hedges’ *g* = 0.292,95% CI: 0.017 to 0.567, *p* = 0.038), squat jump (Hedges’ *g* = 0.730, 95% CI: 0.374 to 1.085, *p* = < 0.001), standing long jump (Hedges’ *g* = 0.298, 95% CI 0.096 to 0.499, *p* = 0.004), throw (Hedges’ *g* = 0.405, 95% CI 0.094 to 0.717, *p* = 0.011) and vertical jump (Hedges’ *g* = 0.407, 95% CI 0.251 to 0.564, *p* = < 0.001) ability. High heterogeneity for squat jump (*I*^2^ = 59%), and noor moderate heterogeneity for other outcomes (*I*^2^ = 0–35%).	+	Moderate to large

* Strength of evidence was categorized into three types: “0,” no association (0–33% of studies supporting a significant association, or no significant meta-analytic effects across four or more studies). “?,” indeterminate/inconsistent association (34–59% of studies and less than four studies supporting a significant association, or a non-significant meta-analytic effect or a significant meta-analytic effect across less than four studies). “− “or “+” strong association (≥ 60% of studies and four or more studies supporting a significant association, or a significant meta-analytic effect across four or more studies). ** Effect size was categorized into three types: Small, Correlation: 0.10 < *r* < 0.30; Hedges’*g*, or Cohen’s d/SMD values of 0.2 to 0.5; standardized *β* 0.10–0.19. Moderate, Correlation: 0.30 < *r* < 0.50; Hedges’*g*, or Cohen’s *d*/SMD values of 0.5 to 0.8; standardized *β* 0.20–0.29. Large, Correlation: *r* > 0.50; Hedges’*g*, or Cohen’s *d* or SMD values > 0.8; standardized *β* > = 0.30. Heterogeneity classification based on *I*^2^: No: 0–25%; moderate 26–20%; high 51–75%; very high 76–100%. CI = Confidence interval; Crl = Credible intervals; CSS = Cross-sectional studies; ES = effect size; FMS = Fundamental Movement Skills; INT = Non-randomized intervention studies; LPA = Light physical activity; LON = Longitudinal studies; MC = Motor competence; MVPA = Moderate-to-vigorous physical activity; PA = Physical activity; RCT = (Clustered) Randomized Controlled Trial studies; SMD = Standardized mean differences; TPA = Total physical activity; VPA = Vigorous Physical activity.

The rating for the level of evidence in support of a pathway was based on an adaptation of the methodology developed by Sallis et al. (2000) and later revised by Barnett et al. (2022). Based on the percentage of findings in the primary studies supporting the association according to the systematic review, a pathway was classified as a non-significant (coded as “0”) when only 0 to 33% of studies reported a significant association, or when no significant meta-analytic effects across four or more studies were found. A pathway was classified as an inconsistent or indeterminate (coded as “?”) association when between 34 and 59% of the primary studies reported a significant association or when less than four primary studies in total reported a significant association. Also, a significant meta-analytic effect across less than four primary studies was considered indeterminate. A pathway was classified as strong (coded as “+” or “-”, depending on the direction of the association) when ≥60% of four or more primary studies supporting a significant association, or a significant meta-analytic effect across four or more primary studies was found. The ≥60% criterion to consider evidence “strong” may appear strict but takes into account that there is considerable concern about publication bias towards significant results in sports science in general (Pesce, 2012) and in the specific domain of motor development/physical activity studies (Barnett et al., 2022).

For the effect sizes for the associations/effects reported we used the meta-analytic estimates when a meta-analysis was present. For systematic reviews without meta-analysis, the average value reported in the review was used, or when no average was reported, we computed the median across reported results for the primary studies. The effect sizes were categorized into three types. Following Cohen (1988) and Peterson and Brown (2005) the effect was considered small if the pooled correlation was between 0.10 < *r* < 0.30, the meta-analytic estimate (Hedges *g*, Cohen’s *d*, or standardized mean difference (SMD)) was between 0.2 to 0.5, or the standardized regression *β* was between 0.05 and 0.25. The effect size was considered moderate if the pooled correlation was between 0.30 < *r* < 0.50, the meta-analytic estimate (Hedges *g*, Cohen’s *d*, or SMD) was between 0.5 to 0.8, or the standardized β was between 0.25–0.45. The effect was large if the pooled correlation was >0.50, the meta-analytic estimate (Hedges’ *g*, Cohen’s d or SMD) was >0.8, or the standardized β was ≥0.45.

### 2.2 Twin studies on the traits in the Stodden model

To address research question 3 on the potential for familial confounding, we retrieved all twin studies on the four traits of the Stodden model. The twin design compares the intra-pair resemblance between two types of sibling relationships; genetically identical twins or monozygotic (MZ) twins, a result of division of a single fertilized egg during an early stage in embryonic development, and non-identical twins or dizygotic (DZ) twins, resulting from two separate fertilized eggs (de Geus, 2023). Consequently, MZ twins are genetically identical and the difference between the twins is due to person-specific environmental factors, i.e., experiences that one of the twins has and the co-twin does not. Dizygotic twins shared on average 50% of their genetic make-up. In contrast to familial aggregation studies, that cannot separate genetic and familial environmental sources of covariance, twin studies can decompose all phenotypic variance of the trait of interest into sources of additive (‘A’) and non-additive (‘D’) genetic influences shared environmental influences (influences shared with other family members, e.g., upbringing; referred to as ‘C’) and person-specific environmental influences (influences unique to the individual; referred to as ‘E’).

#### 2.2.1 Search and selection of behavioral genetics studies

We built on the multiple recent reviews conducted by our group (de Geus, 2023; van der Zee and de Geus, 2019; Zi et al., 2023a; Zi et al., 2023b), but additionally added publications from 2023 and 2024. We searched PubMed and Web-of-Science from January 1980 to December 2024 using the keywords (‘Physical Activity’ OR Exercise OR Sports OR Lifestyle OR Fitness OR Endurance OR Strength OR VO2*) AND (Gene* OR Twin OR Family OR Familial OR Heritability) AND “Humans” [MeSH terms]. Reference sections of selected papers were used to identify additional papers missed by these search terms. We included all studies addressing univariate or multivariate genetic and environmental contributions to one or more of the four traits of the Stodden model using a twin design. Studies with less than 50 complete twin pairs were excluded. We also removed studies reporting on twins with a mean age higher than 18 and. In some studies, the same twin sample was re-used for slightly different research questions. For example, two studies (Huppertz et al., 2017; van der Aa et al., 2010) used overlapping samples with three other studies (Aaltonen et al., 2020; Aaltonen et al., 2013; Huppertz et al., 2016). If the exact same PA trait was used, we only extracted data from the study using the largest sample size. For the studies conducted by Maia and colleagues we used data presented in their 2013 summary (Maia et al., 2013). Finally, we discarded results on light physical activity or sedentary behavior and limited inclusion to total PA, moderate-to-vigorous PA, and leisure time PA including structured sports and exercise participation.

#### 2.2.2 Data extraction

We extracted the authors, year of publication, country, mean age and range of the target population, the Stodden trait(s) examined, the number of MZ and DZ twin pairs, the measurement strategy used for the trait examined, and the estimates of genetic and shared environmental contributions to the traits. Non-additivity (D) was generally not found (or modeled) by the twin studies, so we extracted only the A and C parameters. We preferentially used the A and C parameters estimates from full ACE models; when only reduced AE models were reported we followed the authors in their assumption that C was zero, but note that a small effect of C in these studies may have been undetected due to low power. When sex differences in the A or C parameters were tested, the differential male and female results are reported. When they were not tested or explicitly found to be absent, the same estimates are reported for males and females. When multiple models with different covariates were tested, we selected those that only corrected for age and sex.

## 3 Results

### 3.1 Reporting of confounding by genetic and shared environmental factors

Our search detected 106 systematic reviews, of which 67 added a meta-analysis, on a total of 1,344 unique primary studies that examined one or more of the associations implied by the Stodden model. Table 1 lists all systematic reviews and meta-analyses and Supplementary Table 1 reports on the primary studies, their study design, sample size, and in which systematic reviews and meta-analyses they had been included.

There were 44 systematic reviews addressing the bidirectional MC-PA pathway, 4 systematic reviews on the bidirectional MC-PMC pathway, 5 systematic reviews on the bidirectional PMC-PA pathway and 10 systematic reviews on the path from MC to Fitness. By far the largest amounts of systematic reviews (*n* = 54) were done on the path from physical activity to muscular or cardiorespiratory fitness (PA-Fitness) which is a major area of interest in pediatric exercise science. No reviews included primary studies on the path from fitness to MC or fitness to PA, as manipulation of fitness without also manipulating PA is not feasible in children. Many of the systematic reviews on a specific pathway used overlapping sets of the primary studies, and the more recent reviews generally used the largest number of primary studies.

The patterns in the numbers of the primary studies largely followed that of the systematic reviews (see Supplementary Table 1). There were 426 primary studies reporting on the MC-PA pathway, 102 primary studies on the MC-PMC pathway, 74 primary studies reporting on the PMC-PA pathway, and 100 studies reporting on the MC-fitness pathway. Again, the vast majority of primary studies (*n* = 937) reported on the PA-fitness pathway. As expected, the PA-fitness and MC-fitness studies exclusively tested the effect of MC/PA on fitness traits (not the reverse path). The total number of children and adolescents that have participate in the primary studies testing the Stodden design is over 1.2 million participants.

The very large amount of primary studies described by the systematic reviews should allow us to uncover whether and how past studies on the Stodden model have taken confounding by genetic and shared environmental factors into account. We rely on the authors of the systematic reviews and meta-analyses to have explicitly reported on this. Table 1 reports on our inspection of the discussion sections of the systematic reviews and meta-analyses on text related to potential familial confounding. Our inspection revealed that almost all reviews have taken the potential of unmeasured confounding into account as part of the quality rating the primary studies as prescribed by various guidelines for systematic reviews and meta-analyses, e.g., STROBE, PRISMA, and GRADE (Balshem et al., 2011; Page et al., 2021; von Elm et al., 2007). The quality of the primary studies was often judged to be low to moderate, and it was rarely rated good (see Supplementary Methods Table 4.1). These low scores might in principle have been caused by the majority of primary studies not taking into account potential confounder effects on the associations obtained in the study. We, therefore, considered studies reporting risk of bias as having mentioned ‘general confounding’ (marked by √ in the column of Table 1).

Despite this guideline-enforced attention to the risk of bias, as expressed in study quality scores, explicit mention and discussion of (sources of) familial confounding was very rare. For example, in the main MC-PA pathway, aspects of the family environment were explicitly mentioned as a potential source of confounding in only 5 out of the 44 systematic reviews on this pathway. Most cited shared environmental confounders were parental socioeconomic status and parental social support which may influence both motor competence and physical activity (Barnett et al., 2016; Øglund et al., 2015; Xin et al., 2020). Strikingly, clear mention or discussion of genetic confounding was absent. Only Barnett and colleagues, in their authoritative review of 2022, wrote: “The broad scope of this review meant that we could not assess how other relevant variables (e.g., diet, *genetics*, cultural settings, growth and maturation, cognition, motivation) related to the core variables in the model.” This indirect allusion is the only reference to genetics in the context of confounding in 106 systematic reviews and meta-analyses.

### 3.2 Strength of evidence and effect sizes in the association between motor competence and physical activity by study design

Forty-four systematic reviews (*n* = 22) and meta-analyses (*n* = 22) specifically investigated the direct or mediated paths between motor competence and physical activity in both directions (see Table 2 for details). Of the reviews, 25% reported on a cross-sectional design, 12% on a longitudinal, 32% on an intervention design and 31% were (cluster) RCTs. Many reviews reported on multiple analyses of the MC-PA association from the same primary studies. Separate results were given for the total score for motor competence, and/or scores for all or one of the specific domains of locomotor, object, and stability skills. Separate results were also given for different types of physical activity, including either TPA or MVPA and cardiorespiratory as well as strength training activities.

#### 3.2.1 Cross-sectional studies

Thirteen systematic reviews (*n* = 8) or meta-analyses (*n* = 5) investigated the association between motor competence and physical activity mostly based on primary studies using a cross-sectional design (Barnett et al., 2016; Bingham et al., 2016; Burton et al., 2023; Figueroa and An, 2017; Holfelder and Schott, 2014; Jones et al., 2020; Liu et al., 2023; Logan et al., 2015; Lubans et al., 2010; Poitras et al., 2016; Santos et al., 2023; Xin et al., 2020; Xu et al., 2024). In early childhood, strong evidence for an association between motor competence and TPA or MVPA was found in 12 out of the 22 analyses but indeterminate or no evidence was detected in 10 analyses (Bingham et al., 2016; Figueroa and An, 2017; Jones et al., 2020; Liu et al., 2023; Logan et al., 2015; Santos et al., 2023; Xin et al., 2020; Xu et al., 2024) (see Table 2). The association was much more robust in middle to late childhood, as well as in samples with larger age ranges that included adolescence, with the evidence unanimously strong in all 11 analyses (Barnett et al., 2016; Burton et al., 2023; Holfelder and Schott, 2014; Logan et al., 2015; Lubans et al., 2010; Poitras et al., 2016). Averaged (and meta-analytic) effect sizes were between small and moderate, corresponding to a correlation coefficient of ~0.25. The analyses using the separate fundamental motor skills yielded a very comparable pattern.

#### 3.2.2 Longitudinal studies

Six systematic reviews (*n* = 2) or meta-analyses (*n* = 4) evaluated longitudinal studies in which motor competence was used as the predictor of future physical activity (Barnett et al., 2022; Graham et al., 2022; Holfelder and Schott, 2014; Jones et al., 2020; Øglund et al., 2015; Xin et al., 2020) or in which physical activity was used as the predictor of future motor competence (Barnett et al., 2022; Jones et al., 2020; Xin et al., 2020). Strong evidence for predictive effect of motor competence on physical activity was found in only 3 out of the 7 analyses and indeterminate evidence in 4 analyses (see Table 2). The predictive effects of motor competence seemed to hinge mostly on locomotor and stability skills, with no prediction of future physical activity by object skills (Barnett et al., 2022). No different pattern was seen in early versus middle/late childhood, and the effect sizes were typically small.

When a reverse relationship was examined, indeterminate (3 analyses) or no evidence (2 analyses) was found for a predictive effect of physical activity on future motor competence (Barnett et al., 2022; Jones et al., 2020; Xin et al., 2020).

### 3.3 Intervention studies

Twenty-nine systematic reviews (*n* = 10) or meta-analyses (*n* = 19) (Behringer et al., 2011; Carson et al., 2017; Chen et al., 2024; Collins et al., 2019b; Comeras-Chueca et al., 2021; Dudley et al., 2011; Engel et al., 2018; García-Hermoso et al., 2020; Grady et al., 2025; Hassan et al., 2022; Hesketh et al., 2017; Johnstone et al., 2018; Li et al., 2022; Liu et al., 2020; Lorås, 2020; McDonough et al., 2020; Moon et al., 2024; Norris et al., 2016; Oppici et al., 2022; Poitras et al., 2016; Rico-González, 2023; Sinclair and Roscoe, 2023; Sun and Chen, 2024; Timmons et al., 2012; Van Capelle et al., 2017; Veldman et al., 2021; Wang and Zhou, 2024; Zeng et al., 2017; Zhang et al., 2024) directly addressed the causality in the association between motor competence and physical activity by including intervention studies only. Of these, only two reviews focused on the crucial path of the Stodden model where an increase in motor development would lead to an increase in physical activity levels (Engel et al., 2018; Hesketh et al., 2017). Indeterminate evidence at best was found that intervention on motor competence increases physical activity levels, either in early or in middle childhood samples.

All other 27 reviews included primary studies that tested the reverse effects of physical activity interventions on motor competence, the majority using RCTs. Fifteen of these reviews focused on early childhood. Thirteen of these (12 out of 16 analyses) reported strong evidence for a causal effect of physical activity on motor competence, whether expressed in a total score or in separate scores for locomotor and object control skills. Three reviews (4 out of 16 analyses) reported indeterminate evidence (Johnstone et al., 2018; Liu et al., 2020; Oppici et al., 2022). When reported, the average effect sizes of the PA interventions in early childhood varied from small to large, with larger effect sizes seen when PA was mixed with a deliberate motor skills training component (Chen et al., 2024; Wang and Zhou, 2024). Twelve reviews focused on middle-childhood reporting on RCTs using physical activity intervention to improve motor competence in. Strong evidence (13 out of the 17 analyses) was found that increasing physical activity improved a trait in the MC domain, in all but one review. No, or indeterminate, evidence was found in only 4 of the analyses (see Table 2).

### 3.4 Potential confounding by familial factors

The systematic search for twin studies on the four Stodden traits uncovered only five studies for motor competence (Goetghebuer et al., 2003; Peter et al., 1999; Smith et al., 2017; Zi et al., 2024; Zi et al., 2023b) and none for perceived motor competence. In contrast, very many twin studies were found that reported on cardiorespiratory or muscular fitness and physical activity traits (see Table 3).

**Table 3:** Twin studies on the genetic and shared environmental contribution to the variance in motor competence, physical fitness and physical activity.

Reference	Age Mean	Country	Trait	Instrument	Measurement details	N MZ pairs	N DZ pairs	Heritability males	Heritability females	Shared env. males	Shared env. females
Motor competence											
Peter et al. (1999)	0.5	Israel	Motor milestones	Turn over	The age of first-time being able to fully turn over	30	68	34%	34%	50%	50%
Peter et al. (1999)	0.65	Israel	Motor milestones	Sit up	The age of first-time being able to sit up for a few seconds without support	30	68	31%	31%	56%	56%
Peter et al. (1999)	0.73	Israel	Motor milestones	Stand up	The age of first-time being able to pull up to a standing position without support	30	68	0%	0%	33%	33%
Peter et al. (1999)	1.1	Israel	Motor milestones	Walks (5 s)	The age of first-time being able to walk five steps without support	30	68	22%	22%	67%	67%
Goetghebuer et al. (2003)	0.5	UK	Motor milestones	Roll over	The age of first-time being able to fully turn over	22	62	0%	0%	--	--
Goetghebuer et al. (2003)	0.6	UK	Motor milestones	Crawl	The age of first-time being able to move forwards or backwards either on stomach or on hands and knees	22	62	93%	93%	--	--
Goetghebuer et al. (2003)	0.6	UK	Motor milestones	Sit without support	The age of first-time being able to sit up and maintain the head without rear support	22	62	0%	0%	--	--
Goetghebuer et al. (2003)	0.7	UK	Motor milestones	Stand with support	The age of first-time being able to maintain a standing position by holding on to one’s hand	22	62	72%	72%	--	--
Goetghebuer et al. (2003)	0.8	UK	Motor milestones	Walk with support	The age of first-time being able to walk a few steps by holding on to one’s hand	22	62	90%	90%	--	--
Smith et al. (2017)	0.6	UK	Motor milestones	Sit	The age of first-time being able to sit up without support	1,247	2,705	48%	48%	42%	42%
Smith et al. (2017)	0.8	UK	Motor milestones	Crawl	The age of first-time being able to crawl on hands and knees	1,174	2,502	54%	54%	33%	33%
Smith et al. (2017)	1.1	UK	Motor milestones	Walk	The age of first-time being able to walk a few steps without any support	868	1976	84%	84%	0%	0%
Zi et al. (2023b)	0.5–1.3	Netherlands	Motor milestones (compound score of 5 milestones)	Roll over Sit without support Crawl Stand without support Walk without support	The age of first-time being able to roll over from back to belly sit without support crawl on hands and knees stand without support walk without support	8,043	15,163	52%	53%	38%	39%
Zi et al. (2024)	5	Netherlands	Gross motor skills (compound score of 7 skills)	Hop One-leg stand Throw a ball Kick a ball Catch a ball One foot stair climbing No hands stair climbing	Hop more than 1 time on the same leg Stand on one leg longer than 10s Throw a ball in a fixed direction Kick a ball in a fixed direction Catch a ball Walk down the staircase without putting both feet on a step at the same time Walk down the staircase without using the handrail	6,075	11,114	57%	65%	23%	16%
Total sample						17,473	34,042				
Sample size weighted means								55%	58%	31%	29%
Physical fitness											
Okuda et al. (2005)	6,5	Japan	muscular endurance	sit-ups	dynamic strength and endurance of the abdominal and hip flexor muscles	90	68	0%	0%	51%	51%
Silventoinen et al. (2021)	7	Portugal	muscular strength (compound score of 5 tests)	sit-and-reach standing long jump handgrip sit ups bent arm hang	Sitting reach distance of fingers maintained for 2 s Max distance jumped from standing Maximal isometric strength of arm Dynamic strength and endurance of the abdominal and hip flexor muscles Static upper body strength and endurance	87	129	67%	67%	0%	0%
Silventoinen et al. (2021)	7	Portugal	Coordination and cardiorespiratory (compound score of 4 tests)	Flamingo balance Plate tapping Shuttle run Run/walk 12 min	Dynamic balance Upper body reaction time Multi-stage endurance test Max distance covered in 12 min	87	129	76%	76%	0%	0%
Maes et al. (1996)	10	Belgium	cardiorespiratory	VO_2_max	Maximal exercise test on treadmill	43	61	69%	87%	0%	0%
Maes et al. (1996)	10	Belgium	explosive power	vertical jump	Max jump height from standing position	43	61	65%	65%	0%	0%
Maes et al. (1996)	10	Belgium	flexibility	sit-and-reach	sitting reach distance of fingers maintained for 2 s	43	61	72%	51%	0%	43%
Maes et al. (1996)	10	Belgium	balance	Flamingo balance test	dynamic balance	43	61	41%	41%	0%	0%
Beunen et al. (2003)	11	Belgium	explosive power	vertical jump	Max jump height from standing position	91	105	47%	79%	0%	0%
Beunen et al. (2003)	12	Belgium	explosive power	vertical jump	Max jump height from standing position	91	105	59%	92%	0%	0%
Okuda et al. (2005)	12,5	Japan	explosive power	long jump	Max distance jumped from standing position	90	68	66%	66%	0%	0%
Okuda et al. (2005)	12,5	Japan	flexibility	sit-and-reach	sitting reach distance of fingers maintained for 2 s	90	68	55%	55%	0%	0%
Isen et al. (2014)	12	USA	muscular strength	Handgrip	Maximal isometric strength of static arm	788	466	88%	79%	0%	0%
Okuda et al. (2005)	12,5	Japan	muscular strength	Handgrip	Maximal isometric strength of static arm	90	68	77%	77%	0%	0%
Beunen et al. (2003)	13	Belgium	explosive power	vertical jump	Max jump height from standing position	91	105	85%	77%	0%	0%
Beunen et al. (2003)	14	Belgium	explosive power	vertical jump	Max jump height from standing position	91	105	74%	74%	0%	0%
Peeters et al. (2005)	14	Belgium	explosive power	vertical jump	Max jump height from standing position	42	63	61%	77%	0%	0%
Beunen et al. (2003)	15	Belgium	explosive power	vertical jump	Max jump height from standing position	91	105	63%	91%	0%	0%
Chatterjee and Das (1995)	15	India	explosive power	vertical jump	Max jump height from standing position	30	20	71%	71%	--	--
Silventoinen et al. (2021)	15	Portugal	muscular strength (compound score of 5 tests)	sit-and-reach standing long jump handgrip sit ups bent arm hang	Sitting reach distance of fingers maintained for 2 s Max distance jumped from standing position Maximal isometric strength of static arm Dynamic strength and endurance of the abdominal and hip flexor muscles Static upper body strength and endurance	87	129	73%	73%	0%	0%
Silventoinen et al. (2021)	15	Portugal	Motor ability and cardiorespiratory (compound score of 4 tests)	Flamingo balance Plate tapping Shuttle run Run/walk 12 min	Dynamic balance Upper body reaction time Aerobic capacity multi-stages running test The distance covered in 12 min	87	129	83%	83%	0%	0%
Williams and Gross (1980)	15	New Zealand	Balance	Stabilometer balance	dynamic balance	22	41	27%	27%	49%	49%
Chatterjee and Das (1995)	16	India	Flexibility	sit-and-reach	sitting reach distance of fingers maintained for 2 s	30	20	18%	18%	-	-
Beunen et al. (2003)	16	Belgium	Explosive power	vertical jump	Max jump height from standing position	91	105	0%	82%	65%	0%
Vandenberg (1962)	16	USA	Balance	Beam Balancing	dynamic balance	41	32	48%	48%	--	--
Schutte et al. (2016b)	17	Netherlands	Muscular strength	Handgrip	Maximal isometric strength of static arm	116	111	60%	60%	0%	0%
Schutte et al. (2016a)	17	Netherlands	Cardiorespiratory	VO_2_max	Maximal exercise test on cycle ergometer	115	105	60%	60%	0%	0%
Schutte et al. (2016b)	17	Netherlands	Explosive power	vertical jump	Max jump height from standing position	116	111	49%	49%	0%	0%
Schutte et al. (2016b)	17	Netherlands	Flexibility	sit-and-reach	sitting reach distance of fingers maintained for 2 s	116	111	78%	78%	0%	0%
Schutte et al. (2016b)	17	Netherlands	balance	The Balance Error Scoring System	static balance	116	111	39%	39%	0%	0%
Beunen et al. (2003)	18	Belgium	explosive power	vertical jump	Max jump height from standing position	91	105	63%	78%	0%	0%
Sundet et al. (1994)	18	Norway	cardiorespiratory	VO_2_max	Maximal exercise test on cycle ergometer	436	622	62%	62%	0%	0%
Silventoinen et al. (2008)	18,5	Sweden	muscular strength	Handgrip	Maximal isometric strength of static arm	1,582	1864	66%	66%	3%	3%
Total sample						5,067	5,444				
Sample size weighted means								65%	67%	3%	2%
Physical activity											
Saudino and Zapfe (2008)	2,1	USA	TPA	Actigraph (minimitter) accelerometer	Composite actigraph scores (rate per minute) across two days and four limbs	144	168	32%	32%	54%	54%
Saudino and Zapfe (2008)	2,1	USA	TPA	Toddler Behavior Assessment Questionnaire	Parental frequency rating for PA in 10 specific situations in the past month	144	168	82%	82%	1%	1%
Franks et al. (2005)	7,1	USA	TPA	Doubly labeled water method	Energy expenditure in PA (PAEE) in kcal/day	62	38	41%	41%	35%	35%
Franks et al. (2005)	7,1	USA	TPA	Doubly labeled water method	Physical activity level (PAL) as total EE/ RMR and measured in kcal/day.	62	38	0%	0%	65%	65%
Franks et al. (2005)	7,1	USA	TPA	Doubly labeled water method	Total Energy Expenditure (TEE) in kcal/day	62	38	28%	28%	46%	46%
Huppertz et al. (2012)	7,5	Netherlands	VEB	Multiple Survey items (parental report)	METh/wk. across all sports/exercise activities >3 MET	648	1,320	24%	22%	71%	67%
Zi et al. (2024)	7,5	Netherlands	VEB	Multiple Survey items (parental report)	METh/wk. across all sports/exercise activities >3 MET	1,293	2,339	23%	3%	68%	81%
Huppertz et al. (2016)	7,5	Netherlands	VEB	Multiple Survey items (parental report)	Categories (3) based on METh/wk. low <5; middle >5 and <20; high >20	1,262	2,384	14%	12%	80%	80%
Wood et al. (2008)	8,5	UK	TPA	Actigraph (minimitter) accelerometer	Sum of counts across a 2.5 h lab setting with unstructured breaks	150	113	35%	35%	40%	40%
Zi et al. (2024)	9,8	Netherlands	VEB	Multiple Survey items (parental report)	METh/wk. across all sports/exercise activities >3 MET	1,342	2,393	19%	10%	66%	73%
Huppertz et al. (2016)	9,8	Netherlands	VEB	Multiple Survey items (parental report)	Categories (3) based on METh/wk. low <5; middle >5 and <20; high >20	1,384	2,582	26%	26%	69%	65%
Huppertz et al. (2012)	10,1	Netherlands	VEB	Multiple Survey items (parental report)	METh/wk. across all sports/exercise activities >3 MET	620	1,141	66%	16%	25%	72%
Fisher et al. (2010)	11,0	UK	TPA	Actigraph 7,164 accelerometer	Average activity counts per minute, across 7 consecutive days in counts/min	57	60	14%	14%	63%	63%
Fisher et al. (2010)	11,0	UK	MVPA	Actigraph 7,164 accelerometer	Time spent in MVPA with count >2000 over 7 consecutive days in minutes/d	57	60	28%	28%	39%	39%
Aaltonen et al. (2020)	11,5	Finland	LTPA	Single Survey item (self-report)	Frequency (5) exercise/sports in leisure per week (no …-. every day)	815	1,564	30%	17%	35%	53%
White et al. (2014)	12,0	USA	TPA	3-day physical activity recall (3D-PAR)	METminutes/day across all activities on the three days	72	76	0%	0%	66%	33%
Huppertz et al. (2016)	12,3	Netherlands	VEB	Multiple Survey items (parental report)	Categories (3) based on METh/wk. low <5; middle >5 and <20; high >20	2,615	4,589	31%	27%	62%	65%
Zi et al. (2024)	12,3	Netherlands	VEB	Multiple Survey items (parental report)	METh/wk. across all sports/exercise activities >3 MET	2,583	4,460	31%	29%	54%	57%
Huppertz et al. (2012)	12,3	Netherlands	VEB	Multiple Survey items (parental report)	METh/wk. across all sports/exercise activities >3 MET	1,540	2,746	38%	36%	50%	50%
Maia et al. (2013)	13,0	Portugal	TPA	TRITRAC R3D accelerometer	Sum of counts of the accelerometer across wear time in 5 days	77	85	44%	44%	45%	45%
Stubbe et al. (2005)	13,5	Netherlands	VEB	Multiple Survey items (self-report)	YES/NO regular exercise participation at >4 METs and = > 60 min/wk.	276	370	0%	0%	84%	84%
Pérusse et al. (1989)	14,0	Canada	TPA	B3DPAR three-day PA record (self-report)	Sum of energy expenditure (EE) in all 15-min periods queried (96) across 3 days.	55	56	29%	29%	0%	71%
Pérusse et al. (1989)	14,0	Canada	MVPA	B3DPAR three-day PA record (self-report)	Sum of mean EE in 15-min periods with EE > 4,9 METS across 3 days.	55	56	0%	0%	12%	12%
Aaltonen et al. (2020)	14,0	Finland	LTPA	Single Survey item (self-report)	Frequency (5) exercise/sports in leisure per week (no …-. every day)	742	1,426	45%	32%	15%	28%
Maia et al. (2013)	14,5	Portugal	MVPA	TRITRAC R3D accelerometer	Sum of counts during PA of very vigorous intensity (VVPA)	48	59	72%	42%	0%	0%
Huppertz et al. (2016)	14,6	Netherlands	VEB	Multiple Survey items (self-report)	Categories (3) based on MET h/wk., low <5; middle >5 and <20; high >20	1,451	2,333	43%	40%	36%	43%
Zi et al. (2024)	14,6	Netherlands	VEB	Multiple Survey items (self-report)	METh/wk. across all sports/exercise activities >3 MET	1,527	2,463	51%	18%	19%	48%
Beunen and Thomis (1999)	15,0	Belgium	VEB	Single Survey item (self-report)	Time spent on sports each week within the past year, in number of hours/wk	43	61	83%	44%	0%	54%
Simonen et al. (2004)	15,0	Finland	LTPA	Multiple Interview items (self-report)	Recalled weekly hours spent in any LTPA during adolescence (age 12–18)	147	153	18%	--	37%	--
Maia et al. (2013)	15,0	Portugal	TPA	TRITRAC R3D accelerometer	Sum of counts of the accelerometer across wear time in 5 days	32	19	34%	34%	0%	0%
Haberstick et al. (2014)	15,1	USA	LTPA	Multiple Survey items	Time spent on leisure time physical activities in hours/d	1,374	1,471	7%	54%	43%	0%
Stubbe et al. (2005)	15,5	Netherlands	VEB	Multiple Survey items (self-report)	YES/NO regular exercise participation at >4 METs and = > 60 min/wk.	321	442	0%	0%	78%	78%
Aarnio et al. (1997)	16,0	Finland	LTPA	Multiple Survey items (self-report)	Five categories ranging from very active to inactive in leisure time	378	370	54%	46%	18%	18%
Aaltonen et al. (2013)	16,2	Finland	VEB	Single Survey item (self-report)	Categories (3): Inactive <1x wk.; moderate 1-3x wk.; very active, > 4x wk	769	1743	52%	52%	19%	24%
de Moor et al. (2011)	16,4	Netherlands	VEB	Multiple Survey items (self-report)	YES/NO regular exercise participation at >4 METs and = > 60 min/wk.	656	1,628	42%	36%	44%	52%
de Geus et al. (2003)	16,7	Netherlands	VEB	Multiple Survey items (self-report)	METh/wk. across all sports/exercise activities >4 MET	69	88	79%	79%	0%	0%
Huppertz et al. (2016)	16,9	Netherlands	VEB	Multiple Survey items (self-report)	Categories (3) based on METh/wk. low <5; middle >5 and <20; high >20	959	1,305	56%	49%	27%	31%
Schutte et al. (2019)	16,9	Netherlands	VEB	Multiple Survey items (self-report)	METh/wk. across all exercise activities >4 MET	85	76	67%	67%	0%	0%
Boomsma et al. (1989)	17,0	Netherlands	VEB	Single Survey item (self-report)	YES/NO Sports participation	44	46	77%	35%	0%	0%
Maia et al. (2002)	17,0	Portugal	LTPA	Baecke Questionnaire (self-report)	Composite score on non-exercise related LTPA	203	208	63%	32%	0%	38%
Maia et al. (2002)	17,0	Portugal	VEB	Baecke Questionnaire (self-report)	Composite score based on the two most frequently played sports	203	208	68%	40%	20%	28%
Aaltonen et al. (2013)	17,1	Finland	VEB	Single Survey item (self-report)	Categories (3): Inactive <1x wk.; moderate 1-3x wk.; very active, > 4x wk	724	1,614	44%	50%	24%	26%
Schutte et al. (2019)	17,1	Netherlands	VEB	Multiple Interview items (self-report)	METh/wk. across all exercise activities >4 MET	105	112	81%	81%	0%	0%
Kaartinen et al. (2021)	17,1	Finland	VEB	Multiple Survey items (self-report)	Total number of sports/exercise activities regularly engaged in.	831	1705	58%	40%	0%	26%
Kaartinen et al. (2021)	17,1	Finland	VEB	Multiple Survey items (self-report)	Number of solitary sports/exercise activities regularly engaged in.	831	1705	42%	51%	17%	18%
Kaartinen et al. (2021)	17,1	Finland	VEB	Multiple Survey items (self-report)	Number of team sports/exercise activities regularly engaged in.	831	1705	63%	0%	9%	60%
Stubbe et al. (2005)	17,5	Netherlands	VEB	Multiple Survey items (self-report)	YES/NO regular exercise participation at >4 METs and = > 60 min/wk.	248	395	36%	36%	47%	47%
Aaltonen et al. (2020)	17,6	Finland	LTPA	Single Survey item (self-report)	Frequency exercise/sports in leisure per week (no …-. every day)	678	1,266	54%	43%	16%	16%
Koopmans et al. (1994)	18,0	Netherlands	VEB	Single Survey item (self-report)	YES/NO Sports participation:	578	1,000	48%	48%	38%	38%
Total sample						29,252	50,445				
Sample size weighted means								37%	29%	33%	49%

DZ = Dizygotic twin; EE = Energy expenditure; env. = Environmental; LTPA = Leisure-time physical activity; MET = Metabolic equivalent of task; METh/wk = Methours per week; MVPA = Moderate-to-vigorous physical activity; MZ = Monozygotic twin; PA = Physical activity; PAEE = Energy expenditure in PA; PAL = Physical activity level; RER = Respiratory exchange ratio; RMR = Resting metabolic rate; TEE = Total energy expenditure; TPA = Total physical activity; VEB = Voluntary exercise behavior; VVPA = Very vigorous physical activity.

#### 3.4.1 Genetic and shared environmental effects on motor competence

The top part of Table 3 lists the twin studies on motor competence. High heritability (~55%) and substantial effects of the shared family environment on explained variance (~35%) were found for early motor development in boys and girls as reflected in the timing of early motor milestones development (Goetghebuer et al., 2003; Peter et al., 1999; Smith et al., 2017; Zi et al., 2023b). Shared environmental factors still played a major role in gross motor competence at age 5, but the relative contribution to the total variance was only half to one-third (23% vs. 48% for boys, 16% vs. 48% for girls) of that for motor milestone achievement at age 2 (Zi et al., 2024). At age 5, the individual differences in the mother-reported mastery of seven gross motor skills were even more heritable than age-2 motor milestones attainment in both boys (57% vs. 43%) and girls (65% vs. 44%).

#### 3.4.2 Genetic and shared environmental effects on physical activity

The bottom part of Table 3 lists the twin studies that tested the heritability of physical activity traits. A variety of methods were used, and physical activity was measured either as a total weekly activity score or a score reflecting moderate-to-vigorous activity, often restricted to leisure time activities in particular structured sports and exercise activities. The latter have the advantage of being more reliably assessed by self-report (van der Zee et al., 2020). While the different instruments and traits used induced some heterogeneity in the estimates for heritability and shared environmental influences, this heterogeneity strongly attenuates when only the larger samples are considered. These mostly converge on the sample-size weighted averages across all detected twin studies. On average, 29% of the variance in physical activity in girls was caused by genetic factors and 49% was caused by shared environmental factors. In boys, on average 37% of the variance in physical activity in boys was caused by genetic factors and 33% was caused by shared environmental factors. Heritability estimates from device-based measures of physical activity were very comparable to those from survey-based measures.

#### 3.4.3 Genetic and shared environmental effects on physical fitness

The middle part of Table 3 lists the twin studies that tested the genetic contribution to individual differences in physical fitness in childhood and adolescence, a main mediator in the Stodden model. Three studies reported on the heritability of V.O_2max_, an index of cardiorespiratory fitness. Heritability estimates varied between 60 and 69% (Maes et al., 1996; Schutte et al., 2019; Schutte et al., 2016b; Sundet et al., 1994). The other twin studies focused on explosive power, muscular strength, muscular endurance, flexibility, and balance. For example, muscular strength measured by the handgrip test was assessed in four large studies with an average heritability of 70% (Isen et al., 2014; Okuda et al., 2005; Schutte et al., 2016b; Silventoinen et al., 2008). A number of studies also measured balancing skills often using tests that strongly overlap with tests used in the motor competence domain. Heritability estimates for balance ability ranged from 27 to 48%.(Maes et al., 1996; Schutte et al., 2019; Schutte et al., 2016b; Vandenberg, 1962; Williams and Gross, 1980).

Averaging across the various fitness measures, the sample-size weighted averages for heritability was 65% for boys and 67% for girls. Of note is that the shared environmental factors were not seen to meaningfully contribute to the variance in physical fitness in youth. Any confounding of pathways in the Stodden model relying on fitness, therefore, would be restricted to genetic confounding.

## 4 Discussion

The potential role of motor competence for physical activity received a large boost with the development of the “Stodden model” by Stodden and coworkers in 2008. The Stodden model has inspired a large volume of work, with 106 systematic reviews and/or meta-analyses detected by our literature search reporting on 1,344 primary papers. Many of the systematic reviews and meta-analyses end by a plea for interventions on motor competence in order to ensure future mental and physical health. This enigmatic call-to-intervention reflects the laudable desire to provide societally useful knowledge. This desire does not, however, absolve us form the obligation to provide a strong evidence base that intervention on motor competence is directly causal to increases in youth physical activity levels. Such causality is implied by the original Stodden model.

From a behavioral genetic perspective, we suggested that shared environmental and genetic confounders should be added to the model (see Figure 1). We then examined the systematic reviews and meta-analyses on the Stodden model to see if, and to what extent, this potential familial confounding, and in particular genetic confounding had already been taken into account. We find that confounding by shared environmental factors, which include household/neighborhood/parental rearing style characteristics, did receive cursory attention. However, essentially none of the past systematic reviews or meta-analyses of the cross-sectional or longitudinal observational studies on the Stodden model had considered, let alone ruled out, genetic confounding. We identify the lack of attention to familial confounding, specifically genetic confounding, as a huge knowledge gap in the fields of motor development and youth public health.

In contrast to cross-sectional or longitudinal observational studies, studies using experimental interventions on traits of the Stodden model rule out familial confounding by design. Particularly, in the form of RCTs, they remain the preferred method of assessing causal effects. However, not all paths of the Stodden model are amenable to intervention testing. Changing fitness other than by manipulating physical activity levels is near impossible, as it would require giving, e.g., blood doping to young children. This rules out an experimental test of an effect of fitness on physical activity, an effect hypothesized by one of the mediating pathways of the Stodden model. Likewise, manipulating perceived motor competence without changing actual motor competence is hard, if not unfeasible, which complicates experimental testing of the other mediating pathway in the Stodden model.

Manipulating motor competence by specific motor skill training without overly increasing physical activity levels is feasible in principle, although we note that most interventions on motor competence also increase the amount of physical activity as part of the motor skills training. Nonetheless, these intervention on fundamental motor skills are closest to an experimental test of a causal effect of motor competence on future physical activity in the core pathway of the Stodden model. Strikingly, the only two reviews focused such interventions found indeterminate evidence at best that intervention on motor competence increases physical activity levels, either in early or in middle childhood samples (Engel et al., 2018; Hesketh et al., 2017). This suggests that the hypothesized causal path from motor competence to physical activity does not contribute to the observed association between these two traits.

The most feasible intervention to test a number of the hypothesized paths in the Stodden model are those involving a direct increase in physical activity itself. Indeed, the majority of interventions studies on the Stodden model have focused on the effects of extra physical education lessons, and post-school sports and exercise activities. The systematic reviews on intervention studies provide strong evidence for an effect of physical activity on increased motor competence and fitness, even unanimously so in middle and late childhood. This is in keeping with established knowledge in pediatric exercise and suggest that the cross-sectional association between motor competence and physical activity reflects reverse causality. We note, however, that causal effects of physical activity on motor competence do not rule out the potential for additional familial confounding. In addition, the systematic reviews, often observed a large heterogeneity in the intervention effects on motor competence, both across studies but also within primary study samples. We hypothesize that these individual differences in the response to intervention may largely be attributable to genetic or shared environmental confounding. We, therefore, also searched whether the systematic reviews and meta-analyses on intervention studies on the Stodden model had considered familial factors as potential moderators of variation in outcomes across primary studies applying an intervention. Again, we found no single mention of this possibility in the 106 systematic reviews and meta-analyses on interventions on physical activity.

### 4.1 Potential for familial confounding in the motor development–physical activity pathway

To examine whether the potential for familial confounding was real, we tested the core requirements for such confounding in the motor development - physical activity pathway: (1) the individual differences in motor competence are heritable and/or caused by shared environmental factors, (2) the individual differences in physical activity are heritable and/or caused by shared environmental factors, and (3) there is significant overlap in either the genetic or the shared environmental factors influencing both motor competence and physical activity. We used twin studies on the traits in the Stodden model as our main vehicle.

In our past work, we reviewed family and twin studies on physical activity and fitness traits in childhood and adolescence (de Geus, 2023) but Table 3 provides an update incorporating a number of recent large twin studies on motor competence (Zi et al., 2023a; Zi et al., 2024; Zi et al., 2023b). Across different types of physical activity, across different countries, and across assessment methods (device-based or self-report), we show heritability of physical activity to be around 29% for girls and 37% for boys. Shared environment contributes 49 and 33% to the variance in motor competence in girls and boys, respectively. These findings signal that the first two of the requirements for potential familial confounding are met. The remaining condition for confounding is a substantial overlap in either the genetic or the shared environmental factors influencing *both* motor competence and physical activity. This question has only been directly addressed in a single paper so far (Zi et al., 2024). Using longitudinal data across a 12-year time span in a large population-based sample of MZ and DZ twins, they investigated the prediction of future exercise behavior by early motor development. Early motor development explained 4.3% of the variance in future exercise behavior in boys but only 1.9% in girls. In boys, there was evidence for a significant overlap in the genetic factors influencing early motor development and future exercise behavior, while the regressions between the shared and unique environmental factors were not significant. In girls, neither genetic nor shared and unique environmental regressions were significant, possibly reflecting low power to detect such effects at this very low amount (1.9%) of explained variance.

### 4.2 Mediational pathways in the Stodden model

Three systematic reviews on the Stodden model explicitly examined the evidence for mediation of the association between motor competence and physical activity by perceived motor competence or physical fitness (Barnett et al., 2022; Jones et al., 2020; Robinson et al., 2015). Most comprehensive testing was done by Barnett et al. (2022). They conclude that there is indeterminate evidence for mediation by perceived motor competence across the age range 3 to 18 years. In contrast, mediation by physical fitness of the path between physical activity and (future) motor competence, and the reverse path were supported by strong evidence. However, just as is true for the direct associations, associations mediated by fitness could be due to genetic and shared environmental factors independently acting on the traits in the mediating paths, i.e., genetic or environmental confounding of the MC-fitness and fitness-PA pathways.

Past systematic reviews and meta-analyses had shown that, throughout childhood and adolescence, individual differences in cardiorespiratory and muscular fitness are dominated by genetic factors with only a minimal role for shared environmental factors (Miyamoto-Mikami et al., 2018; Schutte et al., 2016b; Zi et al., 2023a). The update on this literature shown in Table 3 confirms this with, on average, 65–67% of the variance in physical fitness traits in both girls and boys being explained by genetic factors, with negligible shared environmental effects. Given the heritability of motor competence and physical activity, this additional heritability of fitness compromises testing of the mediation of the association between motor competence and physical activity by physical fitness. By correcting the direct path for fitness, we correct for the genetic factors influencing fitness. If these genetic factors partly overlap with either those of motor competence or those of physical activity, this correction would attenuate the association between motor competence and physical activity, mimicking ‘mediation by fitness.’

Direct support for a genetic overlap between fitness traits and physical activity comes from bivariate modeling in adolescent and young adult twin studies that assessed cardiorespiratory and muscular fitness phenotypes and daily regular exercise levels, both cross-sectionally and longitudinally (Schutte et al., 2019). This confirmed that physical fitness and physical activity behaviors are genetically overlapping, with genetic correlations between endurance capacity (VO_2_max) and regular exercise and sports activities in leisure time as high as 0.43. In short, the substantial contributions of genetic factors to motor competence, physical fitness, and physical activity provide a clear potential for genetic confounding in the ‘mediating’ paths using physical fitness.

### 4.3 How to detect and correct for familial confounding in the Stodden model?

We conclude that to more completely test the pathways in the model by Stodden et al. (2008), studies are needed with designs that can detect and account for potential genetic and shared environmental confounding. In the past we were dependent on the use of behavioral genetics studies in (extended) twin families to achieve this. However, despite the elegance of the twin design, not all researchers will have readily access to twin family data. Fortunately, nowadays we can also rely on DNA sampling and genome-wide genotyping in the participants of any cohort, the added costs of which have gone down to ~50 Euro per participant. From these genome-wide genotype data we can create genetic instruments for Mendelian Randomization and extract polygenetic indexes (PGI’s) using the publicly available current (and upcoming) summary statistics of genome-wide association studies (GWAS) on motor competence, physical fitness, and physical activity.

These two methods can be used to test causal hypotheses while controlling for confounding. Mendelian Randomization (MR) is the experiment of nature that comes most close to an RCT in which participants are allocated to different exposure levels independently of confounding. Like an RCT, MR also rules out reverse causality, but uses nature’s randomization to lifetime exposure to DNA variants that influence the exposure (Davey-Smith and Hemani, 2014; Pingault et al., 2018; Speed et al., 2019). These are determined at birth and an advantage of MR over RCTs is that it captures effects of prolonged exposure to, e.g., a propensity to develop motor skills, rather than the mere weeks or months of motor skill training within an RCT. If a genetic instrument is also available for the outcome, e.g., a set of single nucleotide polymorphisms (SNPs) significantly associated with physical activity, bidirectional MR can explicitly test a possible reciprocal causal relationship between exposure (motor competence) and outcome (physical activity).

When our basic concern is to adjust for genetic confounding of an association, e.g., between motor competence and physical activity, the computation of a PGI is another useful strategy (Choi et al., 2020). If a DNA sample with genome-wide SNP genotyping is available for the study participants, the PGI for motor competence and physical activity can be computed using the summary statistics of GWAS on these traits. The PGI is the sum of all the (tiny) genetic effects on the trait across all hundreds or thousands of SNPs that the GWAS detected as meaningfully contributing to the trait (Kujala et al., 2020). If there is genetic confounding, such that the genetic risk for low motor competence is causing (future) low levels of physical activity, then stratifying observed cross-sectional or longitudinal associations for deciles of the PGI for motor competence should lead to attenuation/disappearance of the motor competence—physical activity relationship within each decile of the PGI. If the relationship does not attenuate in the genetically stratified analysis, the association is shown to not depend on genetic confounding. This means that even individuals with a high genetic vulnerability for low motor competence would see their physical activity levels increased by interventions on their motor competence. Indeed, computing the polygenetic propensity for the traits of the Stodden model can also be used to strongly enrich intervention trials. As explained above, the heterogeneity in responsivity to motor competence or physical activity interventions may be partly due to genetic factors. Intervention trials in youth targeting motor competence or physical activity could start to account for this genetic moderation of intervention effects, simply by adding sampling of DNA in the trial participants to the research protocol and using their PGIs as moderator variables.

To apply the MR and PGI approaches to evaluate the Stodden model, we need publicly available summary statistics of large international consortia performing meta-analyses of genome-wide association studies on hundreds of thousands of children. Such summary statistics based on child samples are currently only available early motor coordination (Mountford et al., 2021) but the sample sizes of this GWAS was relatively modest. Larger GWAS efforts on motor milestones in much large samples are currently underway (Gui et al., 2024). However, for physical activity (Doherty et al., 2018; Klimentidis et al., 2018; Wang et al., 2022) and muscular (Willems et al., 2017) and cardiorespiratory fitness (Klevjer et al., 2022), we only have GWAS results based on adult samples, and the authors have not seen clear initiatives for GWAS on these traits in youth. This is where future work is direly needed because we cannot simply assume that the same genetic variants operate throughout the lifespan.

In summary, our synthesis of reviews leads us to fully support the viewpoint expressed earlier by Barnett et al. (2022) and Burton et al. (2023): To truly test the model authored by Stodden et al. (2008) the field is in need of robust longitudinal studies across early childhood and into adolescence. We agree with their recommendations that such longitudinal assessment should aim to include multiple traits from the model, use a combined motor competence assessment (i.e., process and product), and account for biological maturation. However, we offer an update to these recommendations by a strong plea for doing such studies in genetically informative samples that can quantify and account for potential confounding in all pathways of the Stodden model.

## Data Availability

The original contributions presented in the study are included in the article/Supplementary material, further inquiries can be directed to the corresponding author/s.
