# Supplementary material for: Reviewing the association between motor competence and physical activity from a behavioral genetic perspective
Source: Front Psychol. 2025 Apr 28;16:1480631. doi: 10.3389/fpsyg.2025.1480631 (PMC12066450; doi:10.3389/fpsyg.2025.1480631)
Supplement: Supplementary file 2 [file Supplementary_file_2.docx]

Supplementary Material

Reviewing the association between motor competence and physical activity from a behavioral genetic perspective

Yahua Zi^1,3^, Eco JC de Geus^1,2,*^

^1^Department of Biological Psychology, Faculty of Behavioural and Movement Sciences, Vrije Universiteit, Amsterdam, the Netherlands

^2^Amsterdam Public Health Research Institute, Amsterdam University Medical Center, Amsterdam, The Netherlands

^3^School of Exercise and Health, Shanghai University of Sport, Shanghai, China

Contents

[1 Definition of Study Traits 2](#_Toc190432918)

[1.1 Motor Competence 2](#_Toc190432919)

[1.2 Perceived Motor Competence 3](#_Toc190432920)

[1.3 Health Related Fitness 3](#_Toc190432921)

[1.4 Physical Activity 3](#_Toc190432922)

[2 Assessments of Study Traits 4](#_Toc190432923)

[2.1 The Assessments of Motor Competence 4](#_Toc190432924)

[2.2 The Assessments of Perceived Motor Competence 8](#_Toc190432925)

[2.3 The assessments of physical fitness 10](#_Toc190432926)

[2.4 The assessment of physical activity 13](#_Toc190432927)

[3 Search strategy 14](#_Toc190432928)

[4 The assessment of primary study quality reported by the reviews 17](#_Toc190432929)

# Definition of Study Traits

## Motor Competence

Motor competence can be defined as the complete set of motor abilities a person needs to perform all types of goal-directed motor activities required for managing everyday tasks (Clark & Metcalfe, 2002; Henderson & Sugden, 1992; Payne & Isaacs, 2017). Unfortunately, terminology in the field of motor development, and consequently in its assessment, has not been overly consistent (Barnett et al., 2022; Logan, Ross, Chee, Stodden, & Robinson, 2018). Terms used to express motor competence include “fundamental movement skills,”, “motor development,”, “motor proficiency”, “motor coordination”, and “motor abilities”, to name but a few. In our paper, we focus on systematic reviews that included one or more fundamental motor skills (FMS). Fundamental movement skills consist of the domains of basic stability skills (e.g., static and dynamic balance), object control skills, and locomotor skills (Cliff, Okely, Smith, & McKeen, 2009; Gallahue, Ozmun, & Goodway, 2012; Haywood & Getchell, 2014; Jaakkola & Washington, 2013). Locomotor skills involve transporting the body in any direction from one point to another, as during crawling, walking, running, hopping, leaping, jumping, galloping, and skipping. Object control skills involve the control of implements or objects with either the hand or foot, such as kicking or bouncing a ball, striking it with a bat or racquet, or throwing, catching, and stopping a ball. Stability skills provide the capability to sense a shift in the position of body segments relative to each other altering center of mass perturbations and the capability to adjust rapidly and accurately to these alterations such as balancing a narrow beam, demonstrating a stable landing, standing on a single limb or twisting (Bolger et al., 2021; De Meester et al., 2020). The term motor competence is used as a global term that encompasses a score on all or any subset of the fundamental movement skills.

## Perceived Motor Competence

Perceived motor competence refers to the child´s perception of his/her actual motor skills competence level (Estevan & Barnett, 2018). It is one of the constructs that underly the physical self-concept, which in turn is one of the four generally accepted sub-domains of one’s global self-concept, in addition to academic, social, and emotional self-concept (Shavelson, Hubner, & Stanton, 1976). Multiple questionnaires have been developed that adapt to the children’s age to assess perceived motor competence and physical self-perception, sometimes specifically geared to perceived sports competence, or perceived athletic competence.

## Health Related Fitness

Health related fitness is arguably the most complex trait in the Stodden model, encompassing various components that reflect physical health but also exercise and sports ability traits. In general, a distinction can be made between performance-based traits such as endurance, speed, agility, strength and explosive power and health-related traits such as muscular insulin sensitivity, good immune system functioning, high vital capacity, normotensive blood pressure levels, low levels of body fat mass, and visceral obesity. These traits are correlated and yet distinct, but the concept of health-related fitness tends to lump them together. However, in practice many of the primary studies reviewed by the available systematic reviews and meta-analyses on the Stodden model tended to focus mostly on cardiorespiratory fitness and muscular fitness, and less often also on explosive power, balance, and flexibility. These traits are more appropriately summarized by the concept of ‘physical fitness’ and accordingly, we selected reviews that reported one or more of these physical fitness traits. Of note, many of the systematic reviews on the Stodden model also considered the effects motor competence or physical activity on BMI, a trait that should be considered to be part of the broader concept of health-related fitness, not physical fitness. In our synthesis of the systematic reviews and meta-analysis of the five pathways we focus purely on physical fitness and have not reproduced any results related to BMI.

## Physical Activity

Physical activity encompasses all movements produced by skeletal muscles that require energy expenditure (Caspersen, Powell, & Christenson, 1985). Physical activity can be categorized into three levels according to the energy costs of the activity (Ainsworth, Cahalin, Buman, & Ross, 2015; Ainsworth et al., 2012; Ridley, Ainsworth, & Olds, 2008). This cost is often expressed in the metabolic equivalent of task (MET), where one MET is the amount of oxygen consumed while sitting at rest (3.5 ml) per kilogram (kg) body weight per minute (Jetté, Sidney, & Blümchen, 1990). Light-intensity physical activity (LPA) may include activities that are between > 1.5 and < 4.0 METs, such as walking or stretching. Moderate-intensity physical activity (MPA) encompass activities that are between ≥ 4.0 and < 7.0 METs, such as active outdoor play, cycling or swimming. Vigorous-intensity physical activity (VPA) involve more intense efforts, consuming ≥ 7 METs, such as running, playing sports, or engaging in high-intensity interval training (HIIT). Total physical activity (TPA) is the sum of these activities, i.e., LPA + MPA + VPA. The most commonly used outcome is a combination of moderate and vigorous activity (MVPA), as this is the intensity of activity usually recommended in physical activity guidelines. For our review, we allowed a broad range of physical activity definitions (total PA, MVPA, VPA, active commuting, school-based PA), but excluded LPA and sedentary activity.

# Assessments of Study Traits

## The Assessments of Motor Competence

In the primary studies used by the systematic reviews and meta-analyses the assessment of fundamental motor skills has been done by a multifold of different evaluation tools. Supplementary Table 2.1 lists the twelve most used tools in these reviews. Most of these tools were designed to identify, classify, and diagnose motor problems, and scores typically reflect performance compared to a normative child in the same age group, although absolute criteria have also been used (Griffiths, Toovey, Morgan, & Spittle, 2018). A major distinction is made between qualitative assessment by process-based measures (e.g., how the body is positioned, which limbs are moved, and how they move) or quantitative assessment by product-based measures (e.g., such as number of repetitions, whether the ball hits a target, or the distance a ball is thrown). The validity of process- and product-oriented assessments may differ across the type of skill (standing long jump, hop, or throw) and age groups. In process-oriented assessments, maturational differences are not well accounted for and the comparison of a child with an expert performer often results in ceiling effects and floor effects. In product-oriented assessments, no statements about the quality of the skills underlying the movement product can be made. Children can, therefore, be ranked differently when assessed by process- and product-oriented measures, as demonstrated by the broad range of correlations (0.26 <r <0.88) found between process and product- FMS performances (Logan, Barnett, Goodway, & Stodden, 2017).

There is no real gold standard assessment of FMS for children and the available tests vary in their ease of use and interpretability in clinical and research settings. The general consensus is that the use of both process- and product-oriented assessments provides the most comprehensive measurement of FMS competence (Logan et al., 2017; Robinson et al., 2015; Rudd et al., 2016).

**Supplementary Table 2.1** The most used evaluation tools of motor competence among children and adolescents

| **Tool** | **Authors (year)** | **Applicable age** | **Domains & Orientation** | **N Sub-scales** | **Components & N items** | **Time** | **Application area** | **Comments ^a^** |
| --- | --- | --- | --- | --- | --- | --- | --- | --- |
| Bayley Scales of Infant and Toddler Development Screening Test (BSID 4) | (Bayley & Aylward, 2019) | 16 days to 42 months | Gross + fine  Process | 5 | The full scale assesses cognitive (81 items), language (79 items), social-emotional (35 items), adaptive behavior (120) domains. It also has **46 fine motor** and **58 gross motor** **items**).  Item scores range from 0 to 2 (2 is for mastery, 1 for emerging, 0 for not present). The items are summed to scale scores. | 30-70 min, depending on the age of child | Primarily for clinical setting. | Good to excellent reliability, fair to excellent validity.  The developmental process is rapid, so a single assessment score only represents a snapshot of current level. |
| Peabody Development Motor Scales (PDMS-2, PDMS-3) | (Folio & Fewell, 2000; 2023) | 0-5 years | Gross + fine  Product | 6 | Items pertain to body control, body transport, object control, hand manipulation, eye-hand coordination, physical fitness and are summarized in three composite score**: Gross motor, Fine motor, Total motor Index** | 60-90 min | For research and clinical setting | Good reliability, excellent validity. |
| Denver Developmental Screening Test (DDST, DDST-2) | (1992; Frankenburg & Dodds, 1967) | 0-6 years | Gross + fine  Process | 6 | 125 items in the domains of cognitive, language**, fine motor, gross motor**, social, self-mastery skills. Each item is scored as pass, fail, or refused. Children who fail the items that can be passed by 75%-90% of children ‘need caution’. Children who fail the items that can be passed by 90% of children are referred to as ‘delayed’. | Not reported | Primarily for clinical setting. | DDST-2 yielded a high sensitivity rate, identifying 83% of delayed children. But poor specificity as the test also identified more than half of the developmental normal children as delayed. |
| Test of Gross Motor Development (TGMD, TGMD-2, TGMD-3) | (Ulrich, 1985, 2000, 2013) | 3-10.9 years | Gross  Process | 2 | **Locomotion (7 items):** running, galloping, hopping, leaping, horizontal jumping, and sliding. **Object control skills (6 items):** two-handed striking of a stationary ball, catching, kicking, dribbling, overhand throwing, and underhand rolling. Items are rated on 3 to 5 performance criteria, with each criterion is coded as 0 (not performed) or 1 (performed). The locomotion score is maximally 26, the object control skills score is maximally 19. | 15-30 min | For research and clinical settings. | Fair reliability, good validity.  Fine motor and balance skills are not included.  The (summed) total score can be converted to a percentile to enable comparisons of subtests and to other tests. |
| Test zur Erfassung motorischer Basiskompetenzen (MOBAK) | (Herrmann, 2018) | 4-11 years  (4 age groups: 4-5; 6-7; 8-9; 10-11) | Gross  Product | 2 | **Self-movement (4 items):** balancing, Rolling, jumping, and running. **Object movement (4 items):** throwing, catching, bouncing, and dribbling.  The difficulty and complexity of the items increases by age. Performance in each test item is rated as 1 (successful) or 0 (fail). The overall score sums across the 4 items of self-movement and the 4 items of object movement. | ~35 min | For school setting. | Good reliability, excellent validity.  MOBAK is easy to use, requiring easily accessible equipment and uncomplicated evaluation. |
| Körperkoordinationstest für Kinder (KTK) | (Kiphard & Schilling, 1974, 2007; 2017) | 5-14 years | Gross  Product | 1 | **Gross motor coordination (4 items):** The overall score from 4 items is in percentiles. It might indicate motor deficits as well as strength in motor development. | ~20 min | For research and clinical settings. | Good reliability, good validity.  KTK focuses only on dynamic balance skills. Object control and locomotion are not tested. |
| Zurich Neuromotor Assessment (ZNA, ZNA-2) | (Kakebeeke et al., 2018; Largo et al., 2001) | 3-18 years (2 age groups: 3-5 years; 6-18 years) | Gross + fine  Product | 4 | **Pure motor skills (5 items):** repetitive hand, foot, and finger movements, alternating hand and foot movements, and sequential finger movements. **Fine motor adaptive (3 items):** pegboard, bolts, and beads. **Dynamic balance (3 items):** jumping sidewards, chair-rise, and standing long jump. **Static balance (2 items):** one-leg stand with eyes open and with eyes closed. All items are measured in seconds, only the long jump item is measured in centimeters. Age- and gender-based standard scores (z-scores) can be calculated for the components and the total score. | 20-30 min | Primarily for clinical setting. | Good to excellent reliability, good validity. |
| Bruininks-Oseretsky Test of Motor Proficiency (BOTMP, BOT-2) | (Bruininks & Bruininks, 1978; 2005) | 4-21 years | Gross + fine  Product | 8 | The 53 items consist of **six or seven items per component: fine motor precision, fine motor integration, manual dexterity, bilateral coordination, balance, running speed & agility, strength, upper limb coordination**. A subscale score is computed for each of the domains; The composite total motor score sums across the items form all domains. A standardized motor composite standard score (mean: 50, SD: 10) of 40 or less is classified as motor impairment. | BOT-2: 40-60 min  BOT-2 SF: 15-20 min | For research and clinical settings. | BOTMP has good reliability, good validity. The BOT-2 shortform (SF) has only 14 items (one or two items per domain). The validity of the SF Is poor at age 4-6 years, where low mean values might represent a high percentage of zero scores. |
| Movement Assessment Battery (MABC, MABC-2, MABC-3) | (2023; Henderson & Sugden, 1992; 2007) | 3-25 years  (3 age groups:  3-6 years; 7-11 years; 12-25 years) | Gross +fine  Product | 3 | **Manual dexterity skills (4 items)**: drawing circles, posting coins, thread beads, threading lace. **Aiming & catching (3 items)**: catching beanbag, throwing beanbag onto mat, bouncing, and catching with two hands.  **Balance & locomotion (3 items):** One-leg balance, walking heels raised, jumping on mats.  The level of difficulty increases with age. Each item is evaluated on a scale from 0 to 6, the cumulative score from each domain contributes to the overall score. | 20-40 min | For research and clinical settings. | Fair reliability, good validity.  Short completion time and easy administration enables assessing more participants meantime. But the large age ranges result in a loss of sensitivity. |
| McCarron Assessment of Neuromuscular Development (MAND) | (McCarron, 1997) | 3-25 years | Gross +fine  Product | 2 | **Gross motor (5 items)**: finger-nose-finger, grip strength, standing jump, line walking, one leg balance. **Fine motor (5 items)**: beads in box, bead on rod, nut and bolt, finger tap, rod slide.  Item scores are scaled by age and gender, and summed to the total motor competence score, which is standardized to a mean 100 and standard deviation 15. Total scale scores of 85 or less are considered at risk of mild motor impairment. | 25 min | Primarily for clinical setting. | Good reliability, fair validity.  A potential issue is the relevance of MAND and its norms and for populations from countries other than US. |
| Children’s Activity and Movement in Preschool Study (CHAMPS) Motor Skills Protocol (CMSP) | (Williams et al., 2009) | 3-5 years | Gross  Process | 2 | **Locomotor (6 items)**: run, broad jump, slide, gallop, leap, hop. **Object control skills (6 items):** overarm throw, underhand roll, kick, catch, stationary strike, stationary dribble.  The movement is rated as “1” (present) or “0” (not present) for most skills and summed separately to get a score for **locomotor, object control, and total test performance**. | ~ 45 min | For school setting | High reliability, excellent validity. |
| Physical Education Metrics (PE Metrics, 3rd) | (2018; SHAPE America, Couturier, Chepko, & Holt, 2014; Zhu et al., 2011) | Kinder-garten to grade 12 | Gross  Process | 3 | **30** ready-to-use assessments for kindergarten through grade 12 (**65** elementary, **43** middle school, and **22** high school)  **Locomotor:** hopping, sliding, galloping, skipping, running, jumping rope, leaping, etc.  **Object control skills**: throwing underhand/ overhand, catching, dribbling, kicking along the road, kicking in the air, etc.  **Balance**: balance. | Not reported | For school setting | The reliability and validity are not reported. |

**Note: ^a^ retrieved from (Griffiths et al., 2018).**

## The Assessments of Perceived Motor Competence

Multiple questionnaires have been developed that adapt to the children’s age to assess perceived competence (see Supplementary Table 2.2). To accommodate the lower social-cognitive skills of young children who may not be able to construct reality by relating action to sensory information as well older children, Harter and Pike (1984) suggested that tools assessing children’s self-competence should be pictorial, provide an appropriate number of choices per item (e.g., two choices other than four), and include scales adapted to the age range of children. For example, the Pictorial Scale of Perceived Movement Skill Competence developed by Barnett et al. (2015) is based on the Test Gross Motor Development-2, which is specifically designed for children from 4 years old (Barnett, Ridgers, Zask, & Salmon, 2015).

The need for age-appropriate scales is not limited to early childhood, as children in middle childhood may still not be as proficient in interpreting social cues as adolescents. For example, the perceived athletic performance subscale of the Self-Perception Profile for Children developed by Harter is for children aged 8 years and older (Harter, 1985, 1988), whereas the perceived sports competence subscale of the Physical Self-Description Questionnaire developed by Marsh (1996) or the adapted versions of Fox and Corbin’s Physical Self-Perception Profile is specifically targeting adolescents (Fox & Corbin, 1989).

**Supplementary Table 2.2** The most used evaluation tools of perceived motor competence among children and adolescents

| **Tool** | **Authors (year)** | **Applicable age** | **Subscale for PMC** | **N Sub-scales** | **Components & N items** | **Comments** |
| --- | --- | --- | --- | --- | --- | --- |
| The Pictorial Scale of Perceived Movement Skill Competence (PMSC) | (Barnett et al., 2015; Harter & Pike, 1984) | 3-10.9 | Gross | 2 | The PMSC assesses perceived locomotor skills (6 items, run, gallop, hop, leap, horizontal jump, and slide) and object control skills (6 items, striking a stationary ball, stationary dribble, kick, catch, overhand throw, and underhand roll), developed based on the TGMD. Each skill item is represented by two pictures (a good or a poor performance of a skill). Whilst the child is looking at the selected picture, they are then asked to discriminate their level of competence for the “good” picture as “really good at…” (score of four) or “pretty good at…” (score of three), and for the “poor” picture as “not that good at…” (score of two) or “sort of good at…” (score of one). Item scores (1-4) are summed into object control and locomotor subscales (range 6 to 24), and total motor skill (score range 12-48). | Excellent reliability, good validity. |
| The Physical Self-Description Questionnaire (PSDQ) | (Marsh, 1990, 1996) | 8-12 years | Global physical | 9 (short form) | The PSDQ has 70 items in 11 domains: strength, body fat, activity, endurance/fitness, sports competence, coordination, health, appearance, flexibility, global physical, and global esteem. The PSDQ short form (PSDQ-S) has 40 items with the same 11 subscales. | Good reliability, excellent validity. |
| The Self-Perception Profile for Children (SPPC) | (Harter, 1985, 2012b) | 8-15 years | Athletic performance | 6 | The SPPC has 36 items in 6 domains: scholastic competence, social competence, athletic competence, physical appearance, behavioral conduct, global self-worth. Each item contains one positive and one negative description of a specific skill, further options of each description are “really true for me” or “sort of true for me.” Subscale scores are the mean of the 5 item scores. | Good reliability, excellent validity. |
| The Self-Perception Profile for Adolescents (SPPA) | (Harter, 1988, 2012a) | 13-18 years | Athletic performance | 9 | The SPPA consists of 45 (9x5) items in 9 domains: physical appearance, romantic appeal, close friendship, social competence, behavioral conduct, scholastic competence, job competence, athletic competence, global self-worth. Each item has 4 options, with scores ranging from 1 to 4. | Excellent reliability, good validity. |
| The Children and Youth Physical Self-Perception Profile (CY-PSPP) | (Fox & Corbin, 1989; Whitehead, 1995) | 9-14 years | Sports competence | 5 | The CY-PSPP consists of 36 items in 6 domains: body attractiveness, sport competence, physical strength, physical condition, as well as a general physical self-worth and global self-esteem. Each item has two statements with a 4-point scale. First, the child must choose which statement describes the condition and then mark (with X) whether the statement is kind of true or really true. The score of each item ranges from 1 to 4, ranging for each subscale. | Fair reliability, good validity. |
| Tennessee Self-Concept Scale – second edition (TSCS-2) | (Fitts & Warren, 1996) | Child form: 7-14 years  Adult form: >13 years | Physical self-concept | 6 | The TSCS-2 consists of 82 items in 6 domains: physical, moral, personal, family, social, and academic/work self-concept. The items are rated on a 5-point Likert scale, ranging from 1 (always false) to 5 (always true). The TSCS-2 yields two summary scores: total self-concept and conflict. | Fair to good reliability, excellent validity. |
| Perceived Game-specific Soccer Competence Scale (PGSSCS) | (Forsman et al., 2016) | 12-15 years | All scales | 3 | The PGSSCS exclusively rates soccer competence and has 23 items with subscales for three domains: Offensive skills, 1 vs 1 skills, Defensive skills. Each item has a 5-point Likert response from 1 (almost never) to 5 (almost always). | Excellent reliability, excellent validity. |
| Physical Self-Confidence Scale (PSCS) | (McGrane, Belton, Powell, Woods, & Issartel, 2016) | Adolescents | All scales | 1 | The PSCS has 15 questions based on 15 specific skills from TGMD-2 and Victorian skills tests, i.e., run, leap, gallop, slide, horizontal jump, hop, catch, throw, roll, kick, strike, stationary dribble, skip, balance, and vertical jump. Each item is rated on a Likert scale of 1-10, “1” being not confident at all and “10” being very confident. | Excellent reliability, good validity. |
| Physical Self-efficacy Scale | (Bortoli & Robazza, 1997; Ryckman, Robbins, Thornton, & Cantrell, 1982) | 10-20 years | Perceived physical ability | 2 | The scale has 22 items, with subscales in two domains: Perceived Physical Ability (10 items, e.g., strength, speed, and agility) and Physical Self-presentation Confidence (12 items). Each item is rated by 5 points anchored by “1” (“Yes, very much”) and “5” (“No, not at all”). | Good reliability, good validity. |

## The assessments of physical fitness

Supplementary Table 2.3 details the protocols employed to measure specific physical fitness dimensions reported in the systematic reviews and meta-analyses, that is cardiorespiratory fitness, muscular fitness (strength and endurance), explosive power, balance, and flexibility.

Cardiorespiratory fitness refers to the efficiency with which the cardiovascular and respiratory systems supply oxygen to the muscles during sustained physical activity. It is typically measured through indicators such as VO_2_peak (also labeled VO_2_max), which stands for the maximum rate of oxygen consumption during intense exercise. High levels of cardiorespiratory fitness are associated with enhanced overall endurance. Cardiorespiratory fitness is commonly assessed using tests like 20-meter multistage fitness test (also known as the beep test) and either maximal (‘all-out’) or submaximal tests on either bicycle or treadmill ergometer to estimate the oxygen consumption level (VO_2_max), as well as 1200-meter shuttle test. These tests provide valuable insights into aerobic capacity and endurance.

Muscular fitness encompasses various aspects of muscle function, including muscular strength and muscular endurance. Muscular strength is the ability of a muscle to exert force during an activity, while muscular endurance is the ability of a muscle to continue to perform without fatigue. Muscular strength depends on neuromuscular adaptations in: (1) the ability to effectively recruit motor units, (2) the ability to increase motor-unit firing rates, and (3) the efficient coordination of the recruitment of muscle agonists and antagonists. These aspects of neuromuscular adaptations can be improved by strength and resistance training and are critical for the development of any goal-directed movements including fundamental motor skills. They also protect against sports-related injuries. Muscular strength assessments focus on selected sets of muscles. The hand grip test and the mid-thigh pull test measure upper and lower body strength, respectively. Endurance tests, including pull-ups, bent-arm hands, push-ups, sit-ups, and the plank test, evaluate the ability of muscles to sustain repeated contractions over time.

Explosive power is the ability to exert a maximal amount of force in the shortest possible time interval. It is crucial for performance in activities that require sudden bursts of energy, such as sprinting, jumping, and throwing. It is measured through tests like the vertical jump, stand long/ broad jump, the 30-second Wingate test, sprint test, and the 10 × 5 meters shuttle run. These tests assess the power output of muscles in short, high-intensity efforts. Balance refers to the ability to support the body’s position, whether moving or stationary. It is an essential component of overall fitness and is particularly important for preventing falls and injuries.

Balance is assessed through the flamingo balance test, one-leg stand, and beam walk, which measure both static and dynamic balance capabilities. Flexibility is the range of motion available at a joint. It is important for overall functional ability and can prevent injuries by allowing muscles and joints to move through their full range of motion without restriction. Flexibility is evaluated using the sit-and-reach and stand-and-reach tests. These tests measure the flexibility of the lower back and hamstrings, providing an indication of overall flexibility.

**Supplementary Table 2.3** The most used evaluation tools of physical fitness among children and adolescents

|  | **Tool** | **Test protocol** |
| --- | --- | --- |
| **Cardiorespiratory fitness** |  |  |
|  | 20-m multistage fitness test (MSFT) | MSFT is also known as beep test, or 20-m shuttle run. It is a maximal test involving continuous running between two lines 20m apart in time to recorded beeps. The participant stands behind one of the lines facing the second line, begins running when instructed by the recording, and runs between the two lines, turning when signaled by the recorded beeps. The speed at the start is quite slow, the speed increases in each minute. If the line is reached before the beep sounds, the subject must wait until the beep sounds before continuing. If the line is not reached before the beep sounds, the subject is given a warning and must continue to run to the line and try to catch up with the pace within two more “beeps.” The participant will be given a warning the first time failing to reach the line (within two meters) and eliminated after the second warning.  The score is the last level completed, and number of shuttles (20m) reached before the participant is unable to keep up with the recording. This level score can be converted to a VO_2max_ equivalent score. |
|  | Maximal oxygen consumption test (VO_2max_) | The test is performed on an ergometer (treadmill or cycle). The workloads are selected to gradually progress in small increments from moderate to maximal intensity, e.g., as in the famous Bruce Protocol. Oxygen uptake is calculated from measures of total ventilation and the oxygen and carbon dioxide in the expired air, and the maximal level is determined at or near test completion when the RQ is over 1.0 indicating anaerobic contribution.  Results are present as either liters per minute (l/min, the absolute VO_2max_) or milliliters of oxygen per kilogram of body weight (ml/kg/min, the relative VO_2max_). |
|  | 1200m shuttle test | The participant starts at the baseline, runs 20 meters forward, and returns to the baseline, then runs 40 meters forward and returns to the baseline again, and finally run 60 meters forward and return to the baseline. The participant repeats this sequence five times as fast as possible, covering 1200 meters in total.  The score is the total time in seconds to complete the test. |
| **Muscular strength** |  |  |
|  | Hand grip | The participant holds the dynamometer in the hand to be tested, with the arm at the right angles and the elbow by the side of the body. The handle of the dynamometer is adjusted to make the base rested on the first metacarpal (heel of palm) and the handle rested on middle of the four fingers. When ready, the participant squeezes the dynamometer with maximum isometric effort for about 3-5 seconds. No body movement or arm movement is allowed.  The best result from several trials for each hand is recorded, with at least 15 seconds recovery between each effort. Higher value indicates better upper limb strength. |
|  | Mid-thigh pull test (MTP) | MTP is an isometric leg strength test, testing maximal strength of the back and legs. The participant stands upright on the base of a strength dynamometer (with a cable tensiometer) with feet shoulder width apart, lets arms hang straight down to hold the center of the bar with both hands (palms facing toward the body), bents the knees at approximately 100 degrees, bents back slightly forward at the hips, holds head upright, and looks straight ahead. Then, without bending back, the participant pulls as hard as possible on the chain and tries to straighten legs, keeping arms straight and pulling against the weight steadily (no jerky movements). Maximum performance will result when legs are almost straight at the end of the lift. If the dynamometer includes a force plate, the power parameters can also be measured, such as rate of force development and time to peak force. |
| **Muscular endurance** |  |  |
|  | Pull-up | Pull up test measures upper limb strength and endurance. The participant grasps the overhead bar using either overhand grip (palms facing away from body) or underhand grip (palms facing toward body), pulls up the body so the chin raises above the bar, then returns to the position with the arms fully extended. This is repeated for as many pull-ups as they can muster. The modified pull up test is designed to make the test easier for the population with weaker upper limb strength, e.g., younger children. In the modified pull up test, the participant lies straight horizontally, grasps a bar placed just out of reach, and pulls up towards the bar. The score is the total number of correctly completed pull-ups in a period of fixed duration (usually one minute). |
|  | Bent-arm hang | Bent-arm hang is also called the flexed-arm hang, which is one of tests in the Eurofit test battery to test upper limb strength and endurance. The participant is assisted into position, the body lifted to a height so that the chin is level with the horizontal bar. The bar is grasped using an overhand grip (palms are facing away from body), with the hands shoulder width apart. The timing starts when the participant is released and stops when the participant’s chin falls below the level of bar, or the head is tilted backward to enable the chin to stay level with the bar.  The total time held in the flexed-arm position in seconds is recorded. |
|  | Push up | The push-up test is also called press-up test, measuring upper limb strength and endurance. There are several variations of the push-up test with difference in the placement of hands or knees. Standard push up: the participant begins with the hands and toes touching the floor, the body and legs in a straight line, feet slightly apart, the arms at shoulder-width apart, extended and at a right angle to the body. Keeping the back and knees straight, the participant lowers the body to a predetermined point, to touch the ground or some other object, or until there is a 90-degree angle at the elbows, then returns to the starting position with the arms extended. This action is repeated without rest until exhaustion, or until the participant can do no more in rhythm.  The variations of push-up test are designed to make it easier for the population with weak upper limb strength. The modified tests are with the knees resting on the ground or raising the upper body on the chair or to the wall. The score is the total number of correctly completed push-ups in a period of fixed duration (usually two minutes). |
|  | Sit-ups | Sit-up test measures the dynamic strength and endurance of the abdominal and hip flexor muscles. There are several variations with difference in the placement of the hands and feet: 1. Perform with the knees at right angles and the feet are held by the tester; 2. Perform with the knees at right angles and the feet not held; 3. Perform a straight leg sit up with the feet held; 4. Perform a straight leg sit up with the feet not held; 5. Try to reach midpatella with the fingertips of both hands while keeping the arms straight and palms resting on thighs; 6. The arms are folded over chest, with the aim to reach the thighs with both elbows; 7. Touch the back of their earlobes with fingertips and attempts to touch their thighs with their elbows.  The score is the total number of correctly completed sit-ups in a period of fixed duration (usually two minutes). |
|  | Plank | The plank position is to keep the body in prone position, with only hands, forearms and toes touching floor, back straight, stomach and thigh tighten. The participant needs to keep plank position as long as possible until he/she could no longer hold the proper pose (e.g., arching back, dipping hips). The number of seconds to hold the plank pose will be used to assess the performance. |
| **Explosive power** |  |  |
|  | Vertical jump | The participant stands side onto a wall and reaches up with the hand closest to the wall. Keeping the feet flat on the ground, the point of the fingertips is marked or recorded, this is called the standing reach height. Then, the participant stands away from the wall, and leaps vertically as high as possible using both arms and legs to assist in projecting the body upwards. Attempt to touch the wall at the highest point of the jump.  The score is the difference in distance between the standing reach height and the jump height. The best of three attempts is recorded. |
|  | Stand long/ broad jump | The participant stands behind a line marked on the ground with feet slightly apart. A two-foot take-off and landing is used, with swinging of the arms and bending of the knees to provide forward drive. The participant attempts to jump as far as possible, landing on both feet without falling backward.  The measurement is taken from take-offline to the nearest point of contact on the landing (back of heels). The score is the longest distance jumped from three attempts. |
|  | 30-second Wingate test | The Wingate test is a cycle test of anaerobic leg power, conducted over 30 seconds. The participant should first perform a cycling warm up of several minutes, then is instructed to pedal as fast as possible for 30 seconds. The resistance load is adjusted to the pre-determined level, which is usually about 45g/kg body weight (Fleisch) or 75 g/kg body weight (Monark) for adults.  The score is the mean and peak power gained from the test. |
|  | Sprint test | Sprint tests can be performed over varying distances (e.g., 40-meter sprint, 50-yard dash), depending on the factors being tested. The score is the shortest time to finish the distance in three attempts. |
|  | 10 × 5m shuttle run | The test is part of the Eurofit Testing Battery. Participant run back-and-forth over 5 meters, for a total of 50m. The score is the total time taken to complete the 50m course. |
| **Balance** |  |  |
|  | Flamingo balance | It is a part of the Eurofit Testing Battery, testing the strength of leg, pelvic and trunk muscles as well as dynamic balance. The participant stands on one leg on a beam, with the other leg flexed at the knee and the foot of this leg held close to the buttocks. The score is the total number of falls or loss of balance in 60 seconds, the less indicating better balance ability. |
|  | One-leg stand | Assesses static balance. The participant stands on one leg for as long as possible. The score is the total length of time staying in the balance position. |
|  | Beam walk | Assesses dynamic balance of the whole body. The participant is required to walk the length of the beam. The score is the average of the crossing time used in three attempts. An attempt is restarted if the participant steps of the beam. |
| **Flexibility** |  |  |
|  | Sit and reach | The participant sits on the floor with legs stretched out straight ahead, with shoes removed. The soles of feet are placed against the measurement box. Both knees should be locked and pressed flat to the floor, with palms facing down. The participant reaches slowly forward along the measuring line as far as possible and holds the position for at least one-two seconds to make the distance recorded. The modified sit and reach test controls the variable lengths of people’s arms and legs, correcting the limitation of traditional version.  The score is recorded to nearest centimeter or half inch as the distance reached by the tip of the fingers. |
|  | Stand and reach | The participant stands on a raised surface and bend forwards with straight legs. The score is the distance the fingers reach past the toes (negative if above). |

## The assessment of physical activity

Most studies in the systematic reviews and meta-analyses used a mix of studies using parental proxy reports, adolescent’s self-reports, and device-based assessment methods for physical activity. The device-based methods consisted almost exclusively of pedometer or accelerometer devices, worn for periods of 3 to 7 days, with the most frequently used cut-point for a moderate intensity level being 1680 counts/min. Research has repeatedly shown that these objective measures are more precise compared to subjective measures (Chinapaw, Mokkink, van Poppel, van Mechelen, & Terwee, 2010; Welk, 2002) and have the advantage of providing a continuous evaluation of free-living activity (Yang & Hsu, 2010). However, the use of devices is expensive, time consuming and often demanding on the children, making them hard to use in the large population-based investigations. Here, subjective reporting methods is the more versatile and practical choice (Ainsworth et al., 2015). The most common measurement types are subjective measures, which include tools such as physical activity recall questionnaires, structured interviews, diaries or logs, and proxy reporting by teachers or parents (typically used to assess young children).

Physical activity level derived from both subjective or device-based methods is often converted into a continuous time-based estimate in terms of total weekly minutes engaged in LPA, MPA, MVPA, or VPA. The activity level can also be recast into 2 to 3 ordinal categories of e.g., whether children meet the age-specific physical activity guidelines (e.g., WHO), or whether they classify as low, medium, or highly active. In general, subjective reporting is more common in cross-sectional and longitudinal studies, while almost all intervention studies reviewed used device-based physical activity detection.

# Search strategy

The search was conducted twice (one for the original submission stopping at December 31, 2023, one for the revision stopping at January 15, 2025). Articles were considered that appeared one year after the publication of the Stodden model in January, 2008. We searched PubMed, Web of Science (“All Databases” Editions: “All”), and EMBASE.

**Pubmed**

| **Subterm** | **Descriptors** | **Number of studies found** |
| --- | --- | --- |
| **Children** | ("infant*"[Title/Abstract] OR "toddler*"[Title/Abstract] OR "child*"[Title/Abstract] OR "adolescen*"[Title/Abstract] OR "student*"[Title/Abstract] OR "teen*"[Title/Abstract] OR "youth"[Title/Abstract] OR "pediatric*"[Title/Abstract] OR "paediatric*"[Title/Abstract] OR "pube*"[Title/Abstract] OR "juvenil*"[Title/Abstract] OR "school*"[Title/Abstract] OR "youngster*"[Title/Abstract] OR "preschool*"[Title/Abstract] OR "kindergart*"[Title/Abstract] OR "kid"[Title/Abstract] OR "kids"[Title/Abstract] OR "playgroup*"[Title/Abstract] OR "play group*"[Title/Abstract] OR "playschool*"[Title/Abstract] OR "prepube*"[Title/Abstract] OR "preadolescen*"[Title/Abstract] OR "junior high"[Title/Abstract] OR "high school*"[Title/Abstract] OR "middle school*"[Title/Abstract] OR "senior high"[Title/Abstract] OR "young people*"[Title/Abstract] OR "young person*"[Title/Abstract] OR "minor*"[Title/Abstract] OR "elementary school*"[Title/Abstract] OR "primary school*"[Title/Abstract] OR "teen*"[Title/Abstract]) | **2023**: 1,909,250  **2025**: 2,081,346 |
| **Review** | ("systematic review*"[Title/Abstract] OR "meta analys*"[Title/Abstract]) | **2023**: 416264  **2025**: 473,119 |
| Motor Competence  (**MC**) | ("motor skill*"[Title/Abstract] OR "movement skill*"[Title/Abstract] OR "motor development"[Title/Abstract] OR "gross motor"[Title/Abstract] OR "motor performan*"[Title/Abstract] OR "motor proficien*"[Title/Abstract] OR "motor abilit*"[Title/Abstract] OR "object manipulation"[Title/Abstract] OR "motor coordination"[Title/Abstract] OR "actual competen*"[Title/Abstract] OR "object control"[Title/Abstract] OR "locomotor skill*"[Title/Abstract] OR "motor proficiency"[Title/Abstract] OR "motor competen*"[Title/Abstract] OR "movement competenc*"[Title/Abstract] OR "motor fitness"[Title/Abstract] OR "fundamental movement"[Title/Abstract] OR "fundamental motor"[Title/Abstract] OR "basic movement"[Title/Abstract] OR "manipulative skill*"[Title/Abstract] OR "motor function*"[Title/Abstract] OR "athletic skill*"[Title/Abstract] OR "athletic competen*"[Title/Abstract] OR "skill proficiency"[Title/Abstract] OR "movement pattern"[Title/Abstract] OR "motor fitness"[Title/Abstract]) | **2023**: 53,406  **2025**: 59,164 |
| Physical activity  (**PA**) | ("physical activ*"[Title/Abstract] OR "physical* active"[Title/Abstract] OR "physical inactiv*"[Title/Abstract] OR "sedentary"[Title/Abstract] OR "motor activit*"[Title/Abstract] OR "physical education"[Title/Abstract] OR "physical exercise"[Title/Abstract] OR "exercise training"[Title/Abstract] OR "sport"[Title/Abstract] OR "active play"[Title/Abstract] OR "walking"[Title/Abstract] OR "active commut*"[Title/Abstract] OR "energy expenditure"[Title/Abstract]) | **2023**: 285,954  **2025**: 313,996 |
| Health-related fitness  (**HRF**) | ("fitness"[Title/Abstract] OR "physical fitness"[Title/Abstract] OR "musc* fitness"[Title/Abstract] OR "musc* strength"[Title/Abstract] OR "strength"[Title/Abstract] OR "endurance"[Title/Abstract] OR "cardiorespiratory fitness"[Title/Abstract] OR "speed"[Title/Abstract] OR "flexibility"[Title/Abstract] OR "musc* power"[Title/Abstract] OR "muscular endurance"[Title/Abstract]) | **2023**: 590,762  **2025**: 655,550 |
| Perceived motor competence  (**PMC**) | ("perceived motor competence"[Title/Abstract] OR "perceived sport* competence"[Title/Abstract] OR "perceived competence"[Title/Abstract] OR "perceived physical competence"[Title/Abstract] OR "perception* of competence"[Title/Abstract] OR "perceived skill competence"[Title/Abstract] OR "perceived movement skill competence"[Title/Abstract] OR "skill perception*"[Title/Abstract] OR "perceived athletic competence"[Title/Abstract] OR "perceived movement competence"[Title/Abstract] OR "perceived movement skill*"[Title/Abstract] OR "perceived object"[Title/Abstract] OR "perceived locomotor"[Title/Abstract] OR "self-belief"[Title/Abstract] OR "self-concept"[Title/Abstract] OR "self-esteem"[Title/Abstract] OR "self-efficacy"[Title/Abstract] OR "global self-worth"[Title/Abstract] OR "self perception*"[Title/Abstract] OR "self-awareness"[Title/Abstract] OR "self rating*"[Title/Abstract] OR "self-confidence"[Title/Abstract]) | **2023**: 75,690  **2025**: 84,106 |
| Overall search term structure | **Children** AND **Review** AND ((**MC** AND **PA**) OR (**MC** AND **PMC**) OR (**MC** AND **HRF**) OR (**PMC** AND **PA**) OR (**HRF** AND **PA**)) | **2023**: 996  **2025**: 1164 |

**Web of Science**

| **Subterm** | **Descriptors** | **Number of studies found** |
| --- | --- | --- |
| **Children** | TS=(“infant*” OR “toddler*” OR “child*” OR “adolescen*” OR “student*” OR “teen*” OR “youth” OR “pediatric*” OR “paediatric*” OR “pube*” OR “juvenil*” OR “school*” OR “youngster*” OR “preschool*” OR “kindergart*” OR “kid” OR “kids” OR “playgroup*” OR “play-group*” OR “playschool*” OR “prepube*” OR “preadolescen*” OR “junior high” OR “high school*” OR “middle school*” OR “senior high” OR “young people*” OR “young person*” OR “minor*” OR “elementary school*” OR “primary school*” OR "teen*") | **2023**: 6,229,187  **2025**: 6,638,733 |
| **Review** | TS=(“systematic review*” OR meta-analys*) | **2023**: 595,238  **2025**: 674,498 |
| Motor Competence  (**MC**) | TS=(“motor skill*” OR “movement skill*” OR “motor development” OR “gross motor” OR “motor performan*” OR “Motor proficien*” OR “motor abilit*” OR “object manipulation” OR “motor coordination” OR “actual competen*” OR “object control” OR “locomotor skill*” OR “motor proficiency” OR “motor competen*” OR “movement competenc*” OR “motor fitness” OR “fundamental movement” OR “fundamental motor” OR “basic movement” OR “manipulative skill*” OR “motor function*” OR “athletic skill*” OR “athletic competen*” OR “skill proficiency” OR “movement pattern” OR “motor fitness”) | **2023**: 105,222  **2025**: 114,072 |
| Physical activity  (**PA**) | TS=(“physical* activ*” OR “physical inactiv*” OR “sedentary” OR “motor activit*” OR “physical education” OR “physical exercise” OR “exercise training” OR “sport” OR “active play” OR “walking” OR “active commut*” OR “energy expenditure”) | **2023**: 776,774  **2025**: 834,569 |
| Health-related fitness  (**HRF**) | TS=(“fitness” OR “physical fitness” OR “musc* fitness” OR “musc* strength” OR “strength” OR “endurance” OR “cardiorespiratory fitness” OR “speed” OR “flexibility” OR “musc* power" OR “muscular endurance”) | **2023**: 2,548,973  **2025**: 2,795,358 |
| Perceived motor competence  (**PMC**) | TS=(“perceived motor competence” OR “perceived sport* competence” OR “perceived competence” OR “perceived physical competence” OR “perception* of competence” OR “perceived skill competence” OR “perceived skill proficiency” OR “perceived movement skill competence” OR “skill perception*” OR “perceived athletic competence” OR “perceived movement competence” OR “perceived athletic skill*” OR “perceived movement skill*” OR “perception of physical competence” OR “perceptions of physical competence” OR “perceived object“ OR “perceived locomotor” OR “self-belief” OR “self-concept” OR “self-esteem” OR “self-efficacy” OR “global self-worth” OR “self-perception*” OR “self-awareness” OR “self-rating*” OR “self-confidence”) | **2023**: 290,683  **2025**: 313,517 |
| Overall search term structure | **Children** AND **Review** AND ((**MC** AND **PA**) OR (**MC** AND **PMC**) OR (**MC** AND **HRF**) OR (**PMC** AND **PA**) OR (**HRF** AND **PA**)) | **2023**: 2,988  **2025**: 3,316 |

**EMBASE**

| Subterm | **Descriptors** | **Number of studies found** |
| --- | --- | --- |
| **Children** | 'infant*':ab,ti OR 'toddler*':ab,ti OR 'child*':ab,ti OR 'adolescen*':ab,ti OR 'student*':ab,ti OR 'teen*':ab,ti OR 'youth':ab,ti OR 'pediatric*':ab,ti OR 'paediatric*':ab,ti OR 'pube*':ab,ti OR 'juvenil*':ab,ti OR 'school*':ab,ti OR 'youngster*':ab,ti OR 'preschool*':ab,ti OR 'kindergart*':ab,ti OR 'kid':ab,ti OR 'kids':ab,ti OR 'playgroup*':ab,ti OR 'play-group*':ab,ti OR 'playschool*':ab,ti OR 'prepube*':ab,ti OR 'preadolescen*':ab,ti OR 'junior high':ab,ti OR 'high school*':ab,ti OR 'middle school*':ab,ti OR 'senior high':ab,ti OR 'young people*':ab,ti OR 'young person*':ab,ti OR 'minor*':ab,ti OR 'elementary school*':ab,ti OR 'primary school*':ab,ti | **2023**: 2,768,945  **2025**:  2,988,685 |
| **Review** | 'systematic review*':ab,ti OR 'meta analys*':ab,ti | **2023**: 510,692  **2025**: 577,993 |
| Motor Competence  (**MC**) | 'motor skill*':ab,ti OR 'movement skill*':ab,ti OR 'motor development':ab,ti OR 'gross motor':ab,ti OR 'motor performan*':ab,ti OR 'motor proficien*':ab,ti OR 'motor abilit*':ab,ti OR 'object manipulation':ab,ti OR 'motor coordination':ab,ti OR 'actual competen*':ab,ti OR 'object control':ab,ti OR 'locomotor skill*':ab,ti OR 'motor proficiency':ab,ti OR 'motor competen*':ab,ti OR 'movement competenc*':ab,ti OR 'fundamental movement':ab,ti OR 'fundamental motor':ab,ti OR 'basic movement':ab,ti OR 'manipulative skill*':ab,ti OR 'motor function*':ab,ti OR 'athletic skill*':ab,ti OR 'athletic competen*':ab,ti OR 'skill proficiency':ab,ti OR 'movement pattern':ab,ti OR 'motor fitness':ab,ti | **2023**: 77,157  **2025**: 83,887 |
| Physical activity  (**PA**) | 'physical activ*':ab,ti OR 'physical* active':ab,ti OR 'physical inactiv*':ab,ti OR 'sedentary':ab,ti OR 'motor activit*':ab,ti OR 'physical education':ab,ti OR 'physical exercise':ab,ti OR 'exercise training':ab,ti OR 'sport':ab,ti OR 'active play':ab,ti OR 'walking':ab,ti OR 'active commut*':ab,ti OR 'energy expenditure':ab,ti | **2023**: 387,805  **2025**: 419,748 |
| Health-related fitness  (**HRF**) | 'fitness':ab,ti OR 'physical fitness':ab,ti OR 'musc* fitness':ab,ti OR 'musc* strength':ab,ti OR 'strength':ab,ti OR 'endurance':ab,ti OR 'cardiorespiratory fitness':ab,ti OR 'speed':ab,ti OR 'flexibility':ab,ti OR 'musc* power':ab,ti OR 'muscular endurance':ab,ti | **2023**: 689,673  **2025**: 757,454 |
| Perceived motor competence  (**PMC**) | 'perceived motor competence':ab,ti OR 'perceived sport* competence':ab,ti OR 'perceived competence':ab,ti OR 'perceived physical competence':ab,ti OR 'perception* of competence':ab,ti OR 'perceived skill competence':ab,ti OR 'perceived skill proficiency':ab,ti OR 'perceived movement skill competence':ab,ti OR 'skill perception*':ab,ti OR 'perceived athletic competence':ab,ti OR 'perceived movement competence':ab,ti OR 'perceived athletic skill*':ab,ti OR 'perceived movement skill*':ab,ti OR 'perception of physical competence':ab,ti OR 'perceptions of physical competence':ab,ti OR 'perceived object':ab,ti OR 'perceived locomotor':ab,ti OR 'self-belief':ab,ti OR 'self-concept':ab,ti OR 'self-esteem':ab,ti OR 'self-efficacy':ab,ti OR 'global self-worth':ab,ti OR 'self-perception*':ab,ti OR 'self-awareness':ab,ti OR 'self-rating*':ab,ti OR 'self-confidence':ab,ti | **2023**: 92,869  **2025**: 102,099 |
| Overall search term structure | **Children** AND **Review** AND ((**MC** AND **PA**) OR (**MC** AND **PMC**) OR (**MC** AND **HRF**) OR (**PMC** AND **PA**) OR (**HRF** AND **PA**)) | **2023**: 1,079  **2025**: 1,228 |

# The assessment of primary study quality reported by the systematic reviews

Almost all of the systematic reviews and meta-analysis included in this synthesis of reviews made an attempt to judge the quality of the primary studies using a variety of frameworks for the assessment of study quality and risk of bias including the Preferred Reporting Items for Systematic Reviews and Meta-Analysis (PRISMA), STrengthening the Reporting of OBservational studies in Epidemiology (STROBE), Grading of Recommendations, Assessment, Development, and Evaluations (GRADE), Consolidated Standards of Reporting Trials (CONSORT), and Physiotherapy Evidence Database (PEDro) scales as well as a variety of other rating scales (Balshem et al., 2011; Cashin & McAuley, 2020; Cuschieri, 2019a, 2019b; Page et al., 2021). Although there is a large overlap in the conceptual issues rated by scales and checklists of these frameworks (e.g., eligibility criteria and search strategy, inclusion and exclusion criteria, exposure-outcome definition, treatment allocation and blinding, heterogeneity, publication bias) they use very different metrics and direct comparisons across scales is hard. In Supplementary table 4.1 we therefore list the tools used for primary study quality assessment and a narrative summary of the main findings. To harmonize the different verbiage used in the many assessment tools, we provide our overall summary of the review’s judgement on the quality of the primary studies using the categories: poor, fair, moderate, and good.

**Supplementary Table 4.1** Quality of evidence in primary studies as judged by the authors of systematic reviews and meta-analyses.

| **Review** | **Tools for quality assessment** | **Quality of evidence** |
| --- | --- | --- |
| Anico, Wilson, Eyre, and Smith (2022) | PEDro scale | The Pedro scores of the included 7 studies ranged from Poor to Good (one study with Poor, five studies with Fair, one study with Good). Overall: poor |
| Babic et al. (2014) | PRISMA | 20% of studies (13 out of 65) provided an adequate description of the random sampling, process, 92% of studies (59 out of 65) provided an adequate description of the study sample, 98% provided (63 out of 65) a valid measure of physical activity, 73% of studies (47 out of 65) provided a valid measure of physical self-concept but only 27% of studies (17 out of 65) adjusted for covariates. Overall: moderate |
| Barnett et al. (2016) | STROBE | 32% of studies had samples that could be classified as representative of the study population, 58% of studies had minimal missing data, 86% used valid measures of gross motor competence, and 73% used reliable measures of motor competence. Overall: moderate |
| Barnett et al. (2022) | The National Institutes of Health | With regard to longitudinal studies (n=32), the most frequently biased items were item 12 (i.e., blinding of the outcome assessors to the exposure status of participants, 81%), item 3 (i.e., the participation rate of eligible people being at least 50%, 59%), item 13 (i.e., the loss to follow-up after baseline being ≤20%, 59%) and item 5 (i.e., provision of a sample size justification, power description, or variance and effect estimates, 50%).  For intervention,75% of studies had no clear statement regarding the adherence of the treatment groups to the intervention protocols, 50% of the intervention studies did not include concealed treatment allocation (item 3) or had differences between the intervention and control group at baseline (item 6), or had sufficient sample size (item 12).  For mediation studies (n=10), most studies were considered at ‘high’ **risk for bias**. Overall: moderate |
| Bauer, Sperlich, Holmberg, and Engel (2022) | PEDro scale | The PEDro scores of the included 11 studies ranged from 4 to 7 out of 10 (two studies with score 4, one with score 5, seven with score 6 scores, and one score 7). Overall: moderate |
| Beets, Beighle, Erwin, and Huberty (2009) | NR | NR |
| Behm et al. (2017) | NR | NR |
| Behringer, Vom Heede, Matthews, and Mester (2011) | PEDro scale | The PEDro scores of the included 34 studies ranged from 1 (poor quality) to 7 (good quality) and was on average 4.56 ± 0.99. This score is commonly considered to describe a fair methodologic quality of studies. Overall: fair |
| Behringer, vom Heede, Yue, and Mester (2010) | PEDro scale | The PEDro score for the included 42 articles averaged 4.9 of 10 ± 1, with a range from 2 of 10 to 7 of 10. This score is commonly considered to describe a fair methodologic quality of studies. Overall: fair |
| Bingham et al. (2016) | STROBE & CONSORT | The assessment included six criteria, scored as “low”, “moderate”, or “high” quality. None of included ten studies met all six criteria for high quality (low risk of bias), two (20%) met five criteria (rated as “high” quality), seven (70%) met three to four criteria (rated as “moderate” quality), and one (10%) met two criteria (rated as “low” quality). Overall: moderate |
| Braaksma et al. (2018) | PEDro scale | The PEDro scores ranged from 4 (fair quality) to 8 (high quality) out of 9 and was on average 5.3 ± 1.4. Overall: moderate |
| Breslin et al. (2023) | Modified Downs and Black quality checklist | The assessment included 28 criteria, none of included 9 studies met all criteria, five studies (55.6%) met 20-25 criteria (rated as “Good” quality), and the rest four (44.4%) met fifteen to nineteen criteria (rated as “Fair” quality). Overall: moderate |
| Burns, Brusseau, and Fu (2018) | NR | NR |
| Burton et al. (2023) | STROBE & CONSORT | The assessment included six criteria, scored as ‘low risk of bias’, ‘high risk of bias’, or ‘inadequate or unclear description’. No studies met all six criteria for low risk of bias, nine studies (14.8%) met five criteria, eight studies (13.1%) met four criteria, twelve studies (19.7%) met three criteria, thirteen (21.3%) met two criteria, 19 studies (31.1%) met one, 6 studies (9.8%) met none of the criteria for low risk of bias. Overall: fair |
| Carson et al. (2017) | GRADE | The quality of evidence was downgraded from high to very low for 22 randomised studies, and low to very low for 6 longitudinal and cross-sectional studies, due to serious risk of bias, serious inconsistency, and serious indirectness. Quality of the evidence was judged moderate only 2 times, and high only once. Overall: fair |
| Cattuzzo et al. (2016) | STROBE & CONSORT | Sixteen out of 44 studies (36%) demonstrated a score of 5, classified as low risk of bias; the remaining 64% (28 out of 44) were classified as medium risk of bias. Overall: good to moderate |
| Chen et al. (2024) | Modified Downs and Black quality checklist | The assessment included 27 criteria, the majority of questions were rated as either “yes” (1) or “unable to determine/no” (0), except for item five, which was rated as “yes” (2), partial (1), or “no” (0). The maximum score was 32, the quality of the studies was classified as excellent (≥26), good (18-25), fair (13-17), or poor (≤12). Thirteen studies (56.5%) were rated as “good” quality, and the rest ten (43.5%) were rated as “Fair” quality. Overall: moderate |
| Cibinello, Caroliny de Jesus Neves, Janeiro Valenciano, Shizuko Fujisawa, and Augusto Marcal Camillo (2023) | ROB-2 | 46.7% of studies (7 out of 15) were rated as ‘some concerns’, 33.3% of studies (5 out of 15) were rated as ‘high’ risk of bias, only 20% of studies were rated as ‘low’ risk of bias. Overall: moderate |
| Clemente et al. (2022) | PEDro scale | The qualitative classification thresholds are “poor” (<4 points), “fair” (4-5 points), “good” (6-8 points), and “excellent” (9-10 points). Only one study (7.6%) was rated as “excellent” quality, ten (76.9%) were rated as “good” quality, and two (15.2%) were rated as “fair” quality. Overall: good to moderate |
| Collins, Booth, Duncan, and Fawkner (2019) | Quality Assessment Tool for Quantitative Studies | On criteria including **confounding**, 33.3% of the studies were classified as ‘strong’, 33.3% were classified as ‘moderate’ and 33.3% were classified as ‘weak’. Overall: moderate |
| Collins, Booth, Duncan, Fawkner, and Niven (2019) | Quality Assessment Tool for Quantitative Studies | On criteria including **confounding**, 28.6% (two out of seven, 2/7) of the studies were classified as ‘strong’ and 71.4% (five out of seven, 5/7) were classified as ‘moderate’. Overall: good to moderate |
| Comeras-Chueca et al. (2021) | RoB 2 & ROBINS-I | All included sixteen studies (100%) were rated as low risk of bias. Overall: good |
| Costigan, Eather, Plotnikoff, Taaffe, and Lubans (2015) | 8-item PRISMA | All of the studies were found to have moderate to high risk of bias. Criteria to evaluate the overall risk of bias score: low risk of bias studies (8-7), moderate risk of bias (6-4), and high risk of bias (3-0). Overall: moderate to fair |
| Cox, Fairclough, Kosteli, and Noonan (2020) | Modified tools for PA interventions | The assessment tool includes randomisation, blinding, complete outcome data, and outcome measure, scoring 1-4 (i.e., 1=weak, and 4 = very strong). 9.1% (1/11) was rated as very strong, 27.3% (3/11) were rated as strong, 63.7% (7/11) were rated as moderate. Overall: good to moderate |
| Craggs, Corder, van Sluijs, and Griffin (2011) | Modified tools from Kuijpers, van der Windt, van der Heijden, and Bouter (2004) | Items were marked ‘positive’, ‘negative’, or ‘not sufficiently described’. A total score (maximum score: 5) was calculated by adding all positive scores for each assessed study. 44.4% (4/9) were rated as 4-5, 44.4% (4/9) were rated as 2-3, and the other one (11.1%) wasn’t rated. Overall: good to moderate |
| da Silva Bento, Carrasco Páez, and de Mendonça Raimundo (2022) | PEDro Score | The methodological quality of the studies ranged from 4 to 7, with an average of 6. The lost points were mainly due to the blinding of participants and personnel and blinding of the outcome assessment. Overall: good to moderate |
| de Andrade Gonçalves, Augusto Santos Silva, and Gimenes Nunes (2015) | NR | NR |
| De Meester et al. (2020) | STROBE & CONSORT | None of the included studies met all six criteria for low risk of bias, 14.9% met five criteria, 52.8% met four or three criteria, and 32.2% met only two or less of the criteria. Overall: moderate to fair |
| Duncombe et al. (2022) | ROB-2 & ROBINS-I & GRADE | Using ROB-2 for randomisation and ROBINS-I for non-randomisation, 85.7 of studies (36 out of 42) had a ‘high’ risk of bias, mostly related to deviation from the intended intervention and missing data.  Using the GRADE approach, the certainty of the outcomes ranged between ‘very low’ and ‘moderate’. Overall: moderate to fair |
| Eather, Babic, Riley, Costigan, and Lubans (2022) | ROB-2 | 27.3 % (3/11) were rated as low risk of bias, 36.4% (4/11) were rated as some concerns, 27.3% were rated as high risk of bias, and the other one (9.1%) wasn’t rated. Overall: moderate |
| Engel, Broderick, van Doorn, Hardy, and Parmenter (2018) | PEDro scale | Only 42% of the studies scored a good quality score (7/10 or higher), 58% of studies concealed allocation of participants to intervention or control group, 16% of studies blinded the assessors to which group the participants were in, 74% of studies analysed participants as intention to treat. Overall: moderate |
| Errisuriz, Golaszewski, Born, and Bartholomew (2018) | The Downs and Black Methodological Checklist | Only 22.2% (2/9) were rated as high quality, 44.4% (4/9) were rated as medium quality, and 33.3% (3/9) were rated as low quality. Overall: moderate |
| Ferreira, Santos, Palmeira, Fernandes, and Costa (2024) | The RoBANS 2 tool | None of included five studies were rated as low risk of bias, 40% (2/5) were rated as unclear in overall bias, and 60% (3/5) were rated as high in overall bias. Overall: moderate to fair |
| Figueroa and An (2017) | 7 dichotomous criteria | The 7 dichotomous criteria included: 1) was the study a randomized control trial? 2) did the study employ a sample of at least 100 preschoolers 3-5 years of age? 3) was physical activity measured by accelerometer? 4) was motor skill competence measured by a product-based instrument? 5) was the relationship between motor skill competence and physical activity adequately explained in the study? 6) did the study explore potential heterogeneity in the relationship between motor skill competence and physical activity by gender? 7) did the study explore potential heterogeneity in the relationship between motor skill competence and physical activity by age group?  Studies included in the review on average met 3.6 out of 7 quality criteria. Overall: moderate |
| Gäbler, Prieske, Hortobágyi, and Granacher (2018) | PEDro | The quality assessment scores of included fifteen studies ranged 3 to 7, with median of 4. Overall: moderate to fair |
| Garcia-Banos, Angel Rubio-Arias, Manuel Martinez-Aranda, and Jesus Ramos-Campo (2020) | NR | The assessment tool includes eight domains: generation of the sequence, hiding the assignment, blinding of participants and staff, blinding of the result evaluators, incomplete result data, selective reporting of results, and other sources of bias. Six of the included eight studies (75%) had high risk of bias in the domain related to allocation concealment, most studies didn’t provide information on whether participants and/or assessors were blinded or not. Overall: moderate to fair |
| Garcia-Hermoso, Alonso-Martinez, Ramirez-Velez, and Izquierdo (2020) | PEDro | The average total score was 5.1 out of 11, with a range from 4 to 8. Overall: moderate |
| García-Hermoso et al. (2020) | The Physiotherapy Evi- dence Database criteria | The mean total Physiotherapy Evidence Database score was 4.5 (range, 3-8). Overall: moderate to fair |
| Garcia-Hermoso, Ezzatvar, Ramirez-Velez, Olloquequi, and Izquierdo (2021) | The Quality Assessment Tool for Observational Cohort and Cross-sectional Studies | All studies (23) met at least 8 criteria (between 8 and 12 criteria) and were considered to have low-moderate methodological quality. Overall: moderate to fair |
| Grady et al. (2025) | ROB-2 | Only 6.3% (1/16) was rated as low risk of bias, 62.5% (10/16) were rated as some concerns about risk of bias, and 31.2% (5/16) were rated as high risk of bias. Overall: moderate to fair |
| Graham, Azevedo, Wright, and Innerd (2022) | ROB-2 & ROBINS-I | Four studies (21%) were scored as either ‘some concerns/moderate’ or ‘high/serious’ risk of bias due to the handling of missing data and not blinding outcome assessors, 53% (10/19) were rated an overall score of ‘low risk’. Overall: moderate |
| Gralla, McDonald, Breneman, Beets, and Moore (2019) | NR | NR |
| Gutierrez-Garcia, Astrain, Izquierdo, Teresa Gomez-Alonso, and Maria Yague (2018) | The Newcastle-Ottawa Scale (NOS) tool | This tool is composed of eight items for cohort studies and includes three dimensions (selection, comparability and out- come). Several response options are provided for each item. All the included studies were not rated as high risk of bias. Overall: good |
| Hanna, Burns, O'Neill, and Coughlan (2023) | NR | NR |
| Hassan, Liu, McDonough, Su, and Gao (2022) | The Cochrane Risk of Bias Assessment Tool for RCTs | All included studies were rated as low risk of bias on the domains of blinding of outcome assessment (only one was unclear, 94.4%), incomplete outcome data addressed (100%), and selective reporting (100%). 33.3% (6/18), 50% (9/18), and 50% (9/18) were rated as low risk of bias on the domains of blinding of participants and personnel, allocation concealment, and random sequence generation, respectively. Overall: moderate to fair |
| Henriques-Neto et al. (2020) | The Quality Assessment Tool for Quantitative Studies | On criteria including **confounding**, 81.8% of studies (9 out of 11) was rated as moderate quality, and the remaining 11.2% were rated as strong quality. Overall: moderate |
| Hesketh et al. (2017) | Adapted from Wijndaele, Lakshman, Landsbaugh, Ong, and Ogilvie (2009) | Scores out of 6 (or 7 for RCTs) were allocated and categorised accordingly (high quality: C5; medium: 3–4; low: 1–2). Five of included 10 studies (50%) were rated as 6, two (20%) rated as 5, one (10%) as 4, one (10%) as 3, and one wasn’t reported. Overall: moderate to fair |
| Holfelder and Schott (2014) | CONSORT, TREND & STROBE | The included studies included met between 44% to 89% out of nine dichotomous low/high risk of bias criteria. Overall: moderate to good |
| Hui, Wei, Luping, and Nannan (2024) | The Cochrane risk of bias tool | All the 23 included studies were rated as low risk of bias on reporting bias (100%), 65.2% (15/23), 26.1% (6/23), 8.7% (2/23), 34.8% (8/23), 87.0% (20/23) and 95.7% (22/23) as low risk of bias on random sequence generation, allocation concealment, blinding of participants and personnel, blinding of outcome data, incomplete outcome data, and other bias, respectively. Overall: good |
| Jiang et al. (2024) | AHRQ Cross-Sectional Study Quality Assessment Criteria | The included 2 studies, one is low risk of bias, and the other is unclear. Overall: unclear |
| Johnstone, Hughes, Martin, and Reilly (2018) | EPHPP | The included 2 studies, one is moderate risk of bias, and the other is low risk of bias. Overall: moderate to good |
| Jones, Innerd, Giles, and Azevedo (2020) | The observational studies tool from the Evidence for Policy and Practice Information Centre | Study quality ranged from intermediate to high (intermediate: 3 – 5, high > 5; total score is 6). Overall: moderate to good |
| Lang et al. (2018) | Modified from the Cochrane Handbook & GRADE | The overall rating is serious risk of bias for the included four studies. 3 studies (75%) reported using convenience sampling, and 1 study (25%) did not control for sex differences in 20mSRT performance. Overall: poor |
| Larouche, Saunders, Faulkner, Colley, and Tremblay (2014) | The Effective Public Health Practice Project quality assessment tool & GRADE | One study (10%, 10 studies included in total) was rated as strong quality, 70% (7/10) were rated as moderate quality, the remaining 20% (2/10) were rated as weak quality. Overall: moderate to poor |
| Lei and Jun (2022) | Modified from the Cochrane Handbook | All the included fifteen studies were rated as low risk of bias on blinding of outcome assessment (100%), incomplete outcome data (100%), selective reporting (100%), and other bias (100%). 80% (12/15) were rated as low risk of bias on random sequence generation and allocation concealment, but all studies were rated unclear on blinding of participants and personnel. Overall: good |
| Li, Liu, and Ying (2022) | The Cochrane bias risk assessment tool | There were 7 (30.4%) level A (low risk of bias, met ≥ 4 items) studies, 11 (47.8%) level B (moderate bias, met 2-3 items) studies, and 5 (21.7%) level C (highly biased, met only 1 item) studies. Overall: moderate |
| Li et al. (2024) | PEDro | All the thirty-five included studies received scores of ≥6 (100%). Overall: moderate to good |
| Lin, Zhang, Shen, and Zhou (2022) | PEDro | Only one (16.7%, 1/6) was rated as score 8, 16.7% (1/6) and 16.7% (1/6) were rated as score 6 and 5, and the remaining 50% (3/6) were rated as score 4. Overall: moderate to good |
| Liu, Cao, Zhang, Gao, and Qu (2023) | STROBE & CONSORT | 57.1% of studies (28 out of 49) were rated as high quality, the remaining 42.9% (21 out of 49) were rated as moderate quality. Overall: moderate to good |
| Liu, Zeng, McDonough, and Gao (2020) | Adapted from the Cochrane Collaboration’s tool | Eight (80%) of the included nine studies scored equal to or greater than the median score of six and were considered high quality, while one (10%) scored lower than six and was rated low quality. Overall: good |
| Liu, Li, Yuan, and Zhou (2023) | NOS for pooled studies, AHRQ for cross-sectional studies | Study quality ranged from intermediate to high. According to the NOS, all three cohort datasets scored 7 points out of 9. Among the six cross-sectional datasets evaluated using the AHRQ, three scored 7 points and five scored 8 points out of 9. Overall: moderate to good |
| Logan, Kipling Webster, Getchell, Pfeizer, and Robinson (2015) | NR | NR |
| Lorås (2020) | NR | NR |
| Lubans, Boreham, Kelly, and Foster (2011) | STROBE | 40.7% of studies (11 out of 27) scored ≥ 4 (good) on the study quality assessment (total score is 6). Overall: moderate to good |
| Lubans, Morgan, Cliff, Barnett, and Okely (2010) | STROBE & CONSORT | 33.3% of studies (6/18) scored ≥ 5 (high quality), 61.1% scored 3-4 (moderate quality), the remaining one (5.6%) scored 1 (low quality). Overall: moderate to good |
| Martinez-Merino and Rico-González (2024) | 12-point scale for non-RCT studies & the Physiotherapy Evidence Database scale | NA |
| Martin-Smith et al. (2020) | The Cochran tool for assessing risk of bias in randomised trials | 84.2% of studies (16 out of 19) reported low-moderate risk of bias, 10.5% of studies reported moderate risk of bias and 0 studies reported high risk of bias. Overall: moderate to good |
| McDonough, Liu, and Gao (2020) | 8-item quality assessment tool | The 8-item quality assessment tool included eight dichotomous items: randomization, control, pre-post, retention, missing data, power analysis, validity measure, follow-up, score, and effectiveness.  Study quality ranged from 6 to 8 with a median score of 7, with >7 rated as ‘high quality/ low risk of bias’, =7 rated as ‘moderate quality/ medium risk of bias’, and <7 rated as ‘low quality/ high risk of bias’.  Overall: moderate |
| Minatto, Barbosa Filho, Berria, and Petroski (2016) | The Downs and Black Methodological Checklist | The total methodological quality of the included studies was moderate to high, with seven of the 30 studies (23.3%) achieving high methodological quality. Overall: moderate to good |
| Moon et al. (2024) | ROB-2 & ROBINS-I | The quality assessment for C-RCTs or RCTs revealed five studies (45.5, 5/11) as low risk in quality, four studies (36.4%, 4/11) as having some concerns in quality, and one study (9.1%, 1/11) as high risk. For non-RCTs, nine studies (56.3%, 9/16) were evaluated as low risk of bias, six studies (37.5%, 6/16) as moderate quality, and two studies (18.8%, 3/16) as serious risk of bias. Overall: moderate to good |
| Moran et al. (2018) | NR | NR |
| Neil-Sztramko, Caldwell, and Dobbins (2021) | Risk of Bias 1 & GRADE | All included studies were RCTs, more than 70% of studies provided an adequate description of allocation, including randomisation (75.3%, 67 out of 89 studies) and allocation concealment (70.8%, 63 out of 89 studies), but only 16.9% of studies adequately reported the blinding allocation.  For the risk of bias due to incomplete outcome data, 50% of studies (20 out of 40) that assessed physical activity participation or duration were deemed low, 54.8% of studies measuring physical fitness were at low risk of bias. Overall: moderate to good |
| Norris, Hamer, and Stamatakis (2016) | The Effective Public Health Practice Project tool | Of the eight included studies, three (37.5%) were assessed to be moderate, and the rest five (62.5%) were assessed to be weak. Overall: moderate to poor |
| Øglund, Hildebrand, and Ekelund (2015) | Standard quality assessment criteria for evaluating primary research papers from a variety of fields | Study quality scores ranged from 0.73 to 0.95 (range 0-1), so generally good to very good. Overall: good |
| Oppici et al. (2022) | ROB-2 & ROBINS-I | One study (11.1%, 1/9) had a low risk of bias, six studies (66.7%, 6/9) had some concerns, and two studies (22.2%, 2/9) had a high risk of bias. Overall: moderate |
| Peralta, Henriques-Neto, Gouveia É, Sardinha, and Marques (2020) | The Quality Assessment Tool for Observational Cohort and Cross-Sectional Studies | Most studies (19 out of 24, 79.2%) were classified as ‘fair’, one study received a ‘poor’ classification, and the other four studies were considered ‘good’. Overall: fair |
| Pinho, Bagatini, Lisboa, Mello, and Cunha (2024) | TESTEX | The studies included in this review were of medium quality (between 5 and 10 points), no study was of low quality. Overall: moderate |
| Poitras et al. (2016) | GRADE | Study quality scores ranged from very low to moderate. Overall: moderate to poor |
| Pozuelo-Carrascosa, García-Hermoso, Álvarez-Bueno, Sánchez-López, and Martinez-Vizcaino (2018) | The Jadad scale | Fourteen studies (70%, 14/20) fulfilled the criteria regarding randomisation. Six studies (30%, 6/20) fulfilled the criteria regarding randomisation, adherence and dropouts. Six studies (30%, 6/20) used blinded outcome assessors but no study was double blinded (participants and outcome assessors). Overall: moderate to poor. |
| Ramirez-Campillo et al. (2023) | PEDro | The eleven included studies had a median (i.e., nonparametric) PEDro score of 5 points (10 points in total). Overall: fair |
| Reyes-Amigo, Gomez, Gallardo, and Palmeira (2017) | EPHPP | The overall quality of the studies analyzed was weak. Seven studies (70%, 7/10) were considered of weak quality and three were rated as moderate quality. Overall: fair |
| Rico-González (2023) | The Physiotherapy Evidence Database scale | The included nine studies scored from 7 to 10 out of 10 points. Overall: moderate |
| Santos, Burnay, Button, and Cordovil (2023) | ROBINS-I & the JBI Critical Appraisal Checklist for Analytical Cross-Sectional Studies tool | The major problems were related with the risk of bias due to **confounding** (83.3%, 5/6) and exposure measurement (16.7%, 1/6). Overall: poor |
| Sinclair and Roscoe (2023) | MMAT | Three studies (30%, 3/10) scored 7 (maximum 7 points for 7 questions), five (50%, 5/10) scored 6, and two (20%, 2/10) scored 5. Overall: moderate to good |
| Singh et al. (2022) | PEDro | Four studies (30.8%, 4/13) attained fair quality (5 points), and 9 studies (69.2%, 9/13) were of good quality (6–7 points). Overall: moderate |
| Smith et al. (2019) | STROBE & CONSORT | Based on the global rating, 51/77 (66.2%) studies were rated as having a high **risk of bias**, 23/77 (29.9%) were rated having a moderate risk of bias, and 3/77 (3.9%) were rated as having a low risk of bias. Overall: moderate to poor |
| Stojanović, Andrieieva, and Trajković (2024) | the Appraisal RoB tool for Cross-Sectional Studies | AXIS quality assessment indicated that of the 3 studies, 1 was of high quality (33.3%), 1 was of moderate quality (33.3%), and 1 was of low quality (33.3%). Overall: moderate |
| Sun et al. (2013) | The Cochrane Collaboration recommended tool | All trials (100%) performed randomization sequence generation and reported similar baseline characteristics between the intervention and control groups. Overall, details regarding allocation concealment were not adequately described in most trials. Only two trials (18.2%, 2/11) performed allocation concealment and an additional study (9.1%, 1/11) which reported concealment did not implement it. Although blinding of participants and study personnel delivering the interventions was not feasible due to the nature of the trials, two studies (18.2%, 2/11) did report blinding of outcome assessors.  Overall: moderate |
| Sun and Chen (2024) | ROB-2 | There was 1 article with a score of more than six, 6 articles with a score of more than five, 1 article with a score of less than five, and 12 articles all scored a low **risk of bias** (100%). Overall: moderate to good |
| Szeszulski et al. (2019) | QIS | Overall, the study quality was moderate, with an average of 17.9 ± 4.3 (63.9%) of twenty-eight items on the checklist being met. Overall: moderate |
| Timmons et al. (2012) | GRADE | All included studies had no serious **risk of bias**. Overall: good |
| Utesch, Bardid, Busch, and Strauss (2019) | NR | NR |
| Van Capelle, Broderick, van Doorn, R, and Parmenter (2017) | PEDro scale | The average PEDro quality score f or the included studies was moderate at a score of 5.4 ± 1.3 out of 10 (range 3-9). Overall: moderate |
| Veldman, Chin, and Altenburg (2021) | EPHPP | 23.1% of studies (9/39) were rated of high methodological quality, 20.5% of studies (8/39) were rated of moderate methodological quality, and 56.4% (22/39) were rated of weak methodological quality. Overall: moderate to poor |
| Villa-González, Barranco-Ruiz, García-Hermoso, and Faigenbaum (2023) | Quality assessment tool from (Heart & Lung, 2016) | Two different tools: (1) quality assessment of controlled intervention studies and (2) quality assessment tool for before– after (pre–post) studies with no control group.  Six studies (35%, 6/17) were rated as “good”, two (12%, 2/17) as “fair”, and nine (53%, 9/17) as “poor” in their quality assessment. Overall: moderate to poor |
| Wang and Zhou (2023) | Adapted from the statement of Strengthening the Reporting of Observational Studies in Epidemiology and the Evaluation of the Quality of Prognosis Studies in Systematic Reviews | Only one study (33.3%, 1/3) met all five quality criteria, one study (33.3%, 1/3) lacked information on handling **confounders**, the other one (33.3%, 1/3) wasn’t assessed. Overall: poor |
| Wang and Zhou (2024) | ROB-2 | Approximately 75% of the included studies were classified as having a low **risk of bias**. Overall: moderate |
| Woodforde, Alsop, Salmon, Gomersall, and Stylianou (2022) | ROB-2 & ROBINS-I | Three out of four (75%) were rated as high **risk of bias**, and the other one had no information. Overall: poor |
| Wu et al. (2021) | The Jadad Scale | In general, the methodological quality of the included studies was classified as weak. Of all the included studies, only one article (4.8%, 1/21) mentioned double-blinding, and one article (4.8%, 1/21) mentioned randomization and described specific methods, while the remaining articles (90.4%, 19/21) did not implement blinding or did not describe specific randomization methods. Overall: poor |
| Wu et al. (2023) | ROB | Ten studies (15.2%, 10/66) had high a ROB, 38 (57.6%, 38/66) had a moderate ROB, and the remaining 18 (27.3%, 27/66) had a low ROB. Overall: moderate to good |
| Xin et al. (2020) | AHRQ | The assessment of methodological quality was high at an average of 88.7%. In cross-sectional studies, 14 studies were categorized to be of high quality, and 10 studies were classified to be of moderate quality, no studies were classified as low quality. Overall: moderate to good |
| Xu, Shen, and Wen (2024) | AHRQ | The included studies were of moderate to high quality, with a mean score of 7.8 ± 0.7 and an interval of 7–9. The overall quality of the studies was good. Overall: moderate to good |
| Zamorano-Garcia, Infantes-Paniagua, Cuevas-Campos, and Fernandez-Bustos (2023) | Study quality assessment based on Viswanathan and Berkman (2012) | 55% of studies were rated as medium quality, and the remaining 45% were rated as high quality. Overall: moderate to good |
| Zeng et al. (2017) | 8-item quality assessment tool | The 8-item quality assessment tool included eight dichotomous items: randomization, control, pre-post, retention, missing data, power analysis, validity measure, follow-up, score, and effectiveness.  Scores for study quality and **risk of bias** ranged between 5 to 8 (out of 8), so moderate to high quality. Overall: moderate to good |
| Zhang et al. (2024) | PEDro | Thirteen articles (50%, 13/26) achieved a score of five points, signifying acceptable quality. Meanwhile, one piece (3.8%, 1/26) scored six points, eight articles (30.8%, 8/26) scored seven points, and three (11.5%, 3/26) reached eight points, all indicating good quality. Solely one study (3.8%, 1/26) showcased excellent quality and attained a perfect score of 10. The mean score was 6.19, and no low-quality studies were included, indicating that the overall quality of the research was good. Overall: good |
| Zhao, Wang, Niu, and Liu (2023) | The Cochrane risk of bias assessment tool | Eight studies (53.3%, 8/15) had a low **risk of bias** and a high quality, while the remaining 7 studies (46.7%, 7/15) had a moderate risk of bias. Overall: moderate to good |
| Zhou, Li, and Jiang (2024) | PEDro | The studies ranged in their scores from 3 to 10. Only two studies (6.7%, 2/30) achieved high-quality scores (≥ 8). One study (3.3%, 1/30) scored below 4. Blinding techniques ranged from 0 to 3 in this study, with only one study (3.3%, 1/30) scoring 3 and four (13.3%, 4/30) scoring 1; Fifteen studies (50%, 4/30) scored 0. Overall: moderate to poor |
| AHRQ = Agency for Healthcare Research and Quality guidance. If quality assessment scores were from 8 to 11, the study was considered as high methodological quality; 4-7, moderate quality; <3, low quality.  The Cochran tool for assessing risk of bias in randomised trials, has 8 items classifies as ‘yes’ (include the item), ‘no’ (not include the item), or ‘not available’ (unknown whether the study includes the item).  CONSORT = Consolidated Standards of Reporting Trials  EPHPP = the Quality Assessment Tool for Quantitative Studies  GRADE = the Grading of Recommendations Assessment, Development, and Evaluation. GRADE has four levels of certainty for the evidence, ‘high’, ‘moderate’, ‘low’, and ‘very low’.  MMAT = the Mixed Methods Appraisal Tool  The National Institutes of Health are quality assessment tools both for controlled intervention studies and for observational cohort and cross-sectional studies. Both tools include 14 criteria, which were scored with a “yes” (low risk of bias), a “no” (high risk of bias), or a “could not be determined (CD)”, to assess a study’s risk of bias.  NOS = the Newcastle-Ottawa Scale, scores ranged from 0 to 9, and studies with NOS scores > 6 were considered positive.  NR = Not Reported  PEDro = the modified Physiotherapy Evidence Database Scale, an 11-item scale designed for measuring the methodological quality of RCTs.  PRISMA = The Preferred Reporting Items for Systematic reviews and Meta-Analyses, including 27 items.  The Physiotherapy Evidence Database scale. Eleven items are measured, the scale scores range from 0 to 10, with the criterion 1 not included in the final score. The score that each section receives can be from 0 (“no”) to 1 (“yes”).  QIS = Quality Index Score, scores range from 0 to 28 (higher scores equal stronger quality). The QIS reports on several aspect of bias including quality of reporting, external validity, measurement/intervention bias, subject selection bias, and whether the study was appropriately powered.  The Quality Assessment Tool for Observational Cohort and Cross-sectional Studies, has 14 items classified as ‘yes’, ‘no’, or ‘not reported’.  The Quality Assessment Tool for Quantitative Studies, a 19-item checklist to assess: selection bias, study design, confounders, blinding, data collection methods, withdrawals and dropouts, intervention integrity, and analyses. Each item is classified as ‘strong’, ‘moderate’, or ‘weak’.  Risk of Bias 1, has 6 domains: sequence generation, allocation concealment, blinding of participants and personnel, blinding of outcome assessors, incomplete outcome data, and selective reporting. Each domain is assigned assessment of low, high, or unclear risk of bias.  RoB-2 = Risk of Bias 2, designed for randomized studies. The following five items were evaluated: bias arising from the randomization process, bias due to deviations from the intended interventions, bias due to missing outcome data, bias in the measurement of the outcome, and bias in the selection of the reported result. Each category received a bias score of ‘low’, ‘some concerns’, or ‘high’.  ROBINS-I = the Risk of Bias in Non-Randomised Studies, designed for non-randomised studies.  STROBE = the Strengthening the Reporting of Observation studies in Epidemiology, has 6 items classified as ‘0’ (absent or inadequately described) or 1 (explicitly described and present).  Study quality assessment based on Viswanathan and Berkman (2012), has 11 items classified as ‘yes’ or ‘no’ based on whether the item exists in the primary study.  TESTEX = The Tool for the assEssment of Study qualiTy and reporting in EXercise  TREND = the Transparent Reporting of Evaluation with Nonrandomized Design | | |

References

Ainsworth, B., Cahalin, L., Buman, M., & Ross, R. (2015). The current state of physical activity assessment tools. *Progress in Cardiovascular Diseases, 57*(4), 387-395.

Ainsworth, B. E., Caspersen, C. J., Matthews, C. E., Masse, L. C., Baranowski, T., & Zhu, W. (2012). Recommendations to improve the accuracy of estimates of physical activity derived from self report. *Journal of Physical Activity & Health, 9 Suppl 1*(0 1), S76-84. doi:10.1123/jpah.9.s1.s76

Anico, S., Wilson, L., Eyre, E., & Smith, E. (2022). The effectiveness of school-based run/walk programmes to develop physical literacy and physical activity components in primary school children: A systematic review. *Journal of Sports Sciences, 40*(22), 2552-2569. doi:10.1080/02640414.2023.2174720

Babic, M. J., Morgan, P. J., Plotnikoff, R. C., Lonsdale, C., White, R. L., & Lubans, D. R. (2014). Physical activity and physical self-concept in youth: systematic review and meta-analysis. *Sports Medicine, 44*(11), 1589-1601. doi:10.1007/s40279-014-0229-z

Balshem, H., Helfand, M., Schunemann, H. J., Oxman, A. D., Kunz, R., Brozek, J., . . . Guyatt, G. H. (2011). GRADE guidelines: 3. Rating the quality of evidence. *Journal of Clinical Epidemiology, 64*(4), 401-406. doi:10.1016/j.jclinepi.2010.07.015

Barnett, L. M., Lai, S. K., Veldman, S. L. C., Hardy, L. L., Cliff, D. P., Morgan, P. J., . . . Okely, A. D. (2016). Correlates of Gross Motor Competence in Children and Adolescents: A Systematic Review and Meta-Analysis. *Sports Medicine, 46*(11), 1663-1688. doi:10.1007/s40279-016-0495-z

Barnett, L. M., Ridgers, N. D., Zask, A., & Salmon, J. (2015). Face validity and reliability of a pictorial instrument for assessing fundamental movement skill perceived competence in young children. *Journal of Science and Medicine in Sport, 18*(1), 98-102. doi:10.1016/j.jsams.2013.12.004

Barnett, L. M., Webster, E. K., Hulteen, R. M., De Meester, A., Valentini, N. C., Lenoir, M., . . . Rodrigues, L. P. (2022). Through the Looking Glass: A Systematic Review of Longitudinal Evidence, Providing New Insight for Motor Competence and Health. *Sports Medicine, 52*(4), 875-920. doi:10.1007/s40279-021-01516-8

Bauer, N., Sperlich, B., Holmberg, H. C., & Engel, F. A. (2022). Effects of High-Intensity Interval Training in School on the Physical Performance and Health of Children and Adolescents: A Systematic Review with Meta-Analysis. *Sports Med Open, 8*(1), 50. doi:10.1186/s40798-022-00437-8

Bayley, N., & Aylward, G. (2019). Bayley scales of infant and toddler development fourth edition (Bayley-4). *Bloomington, MN: NCS Pearson*.

Beets, M. W., Beighle, A., Erwin, H. E., & Huberty, J. L. (2009). After-School Program Impact on Physical Activity and Fitness. A Meta-Analysis. *American Journal of Preventive Medicine, 36*(6), 527-537. doi:10.1016/j.amepre.2009.01.033

Behm, D. G., Young, J. D., Whitten, J. H. D., Reid, J. C., Quigley, P. J., Low, J., . . . Granacher, U. (2017). Effectiveness of Traditional Strength vs. Power Training on Muscle Strength, Power and Speed with Youth: A Systematic Review and Meta-Analysis. *Frontiers in Physiology, 8*. doi:ARTN 423 10.3389/fphys.2017.00423

Behringer, M., Vom Heede, A., Matthews, M., & Mester, J. (2011). Effects of strength training on motor performance skills in children and adolescents: a meta-analysis. *Pediatric Exercise Science, 23*(2), 186-206. doi:10.1123/pes.23.2.186

Behringer, M., vom Heede, A., Yue, Z. Y., & Mester, J. (2010). Effects of Resistance Training in Children and Adolescents: A Meta-analysis. *Pediatrics, 126*(5), E1199-E1210. doi:10.1542/peds.2010-0445

Bingham, D. D., Costa, S., Hinkley, T., Shire, K. A., Clemes, S. A., & Barber, S. E. (2016). Physical Activity During the Early Years: A Systematic Review of Correlates and Determinants. *American Journal of Preventive Medicine, 51*(3), 384-402. doi:10.1016/j.amepre.2016.04.022

Bolger, L. E., Bolger, L. A., O'Neill, C., Coughlan, E., O'Brien, W., Lacey, S., . . . Bardid, F. (2021). Global levels of fundamental motor skills in children: A systematic review. *Journal of Sports Sciences, 39*(7), 717-753. doi:10.1080/02640414.2020.1841405

Bortoli, L., & Robazza, C. (1997). Italian version of the Perceived Physical Ability Scale. *Perceptual and Motor Skills, 85*(1), 187-192. doi:10.2466/pms.1997.85.1.187

Braaksma, P., Stuive, I., Garst, R. M. E., Wesselink, C. F., van der Sluis, C. K., Dekker, R., & Schoemaker, M. M. (2018). Characteristics of physical activity interventions and effects on cardiorespiratory fitness in children aged 6-12 years-A systematic review. *Journal of Science and Medicine in Sport, 21*(3), 296-306. doi:10.1016/j.jsams.2017.07.015

Breslin, G., Hillyard, M., Brick, N., Shannon, S., McKay-Redmond, B., & McConnell, B. (2023). A systematic review of the effect of The Daily Mile™ on children's physical activity, physical health, mental health, wellbeing, academic performance and cognitive function. *PloS One, 18*(1), e0277375. doi:10.1371/journal.pone.0277375

Bruininks, R. H., & Bruininks, B. D. (1978). Bruininks-Oseretsky test of motor proficiency.

Bruininks, R. H., & Bruininks, B. D. (2005). *Bruininks-Oseretsky test of motor proficiency - second edition (BOT-2)* Minneapolis: Pearson.

Burns, R. D., Brusseau, T. A., & Fu, Y. (2018). Moderators of School-Based Physical Activity Interventions on Cardiorespiratory Endurance in Primary School-Aged Children: A Meta-Regression. *International Journal of Environmental Research and Public Health, 15*(8). doi:10.3390/ijerph15081764

Burton, A. M., Cowburn, I., Thompson, F., Eisenmann, J. C., Nicholson, B., & Till, K. (2023). Associations Between Motor Competence and Physical Activity, Physical Fitness and Psychosocial Characteristics in Adolescents: A Systematic Review and Meta-analysis. *Sports Medicine, 53*(11), 2191-2256. doi:10.1007/s40279-023-01886-1

Carson, V., Lee, E. Y., Hewitt, L., Jennings, C., Hunter, S., Kuzik, N., . . . Tremblay, M. S. (2017). Systematic review of the relationships between physical activity and health indicators in the early years (0-4 years). *BMC Public Health, 17*(Suppl 5), 854. doi:10.1186/s12889-017-4860-0

Cashin, A. G., & McAuley, J. H. (2020). Clinimetrics: Physiotherapy Evidence Database (PEDro) Scale. *Journal of Physiotherapy, 66*(1), 59. doi:10.1016/j.jphys.2019.08.005

Caspersen, C. J., Powell, K. E., & Christenson, G. M. (1985). Physical activity, exercise, and physical fitness: definitions and distinctions for health-related research. *Public Health Reports, 100*(2), 126.

Cattuzzo, M. T., Dos Santos Henrique, R., Re, A. H., de Oliveira, I. S., Melo, B. M., de Sousa Moura, M., . . . Stodden, D. (2016). Motor competence and health related physical fitness in youth: A systematic review. *Journal of Science and Medicine in Sport, 19*(2), 123-129. doi:10.1016/j.jsams.2014.12.004

Chen, D., Zhao, G., Fu, J., Shun, S., Su, L., He, Z., . . . Shen, F. (2024). Effects of structured and unstructured interventions on fundamental motor skills in preschool children: a meta-analysis. *Frontiers in Public Health, 12*. doi:10.3389/fpubh.2024.1345566

Chinapaw, M. J., Mokkink, L. B., van Poppel, M. N., van Mechelen, W., & Terwee, C. B. (2010). Physical activity questionnaires for youth: A systematic review of measurement properties. *Sports Medicine, 40*(7), 539–563. doi:10.2165/11530770-000000000-00000

Cibinello, F. U., Caroliny de Jesus Neves, J., Janeiro Valenciano, P., Shizuko Fujisawa, D., & Augusto Marcal Camillo, C. (2023). Effects of Pilates in children and adolescents - A systematic review and meta-analysis. *Journal of Bodywork and Movement Therapies, 35*, 400-412. doi:10.1016/j.jbmt.2023.04.028

Clark, J. E., & Metcalfe, J. S. (2002). The mountain of motor development: a metaphor. In J. E. Clark & J. H. Humphrey (Eds.), *Motor Development: Research and Reviews* (Vol. 2, pp. 163–190). Reston, VA: National Association of Sport and Physical Education.

Clemente, F. M., Moran, J., Ramirez-Campillo, R., Oliveira, R., Brito, J., Silva, A. F., . . . Sarmento, H. (2022). Recreational Soccer Training Effects on Pediatric Populations Physical Fitness and Health: A Systematic Review. *Children (Basel), 9*(11). doi:10.3390/children9111776

Cliff, D. P., Okely, A. D., Smith, L. M., & McKeen, K. (2009). Relationships Between Fundamental Movement Skills and Objectively Measured Physical Activity in Preschool Children. *Pediatric Exercise Science, 21*(4), 436-449. doi:DOI 10.1123/pes.21.4.436

Collins, H., Booth, J. N., Duncan, A., & Fawkner, S. (2019). The effect of resistance training interventions on fundamental movement skills in youth: a meta-analysis. *Sports Med Open, 5*(1), 17. doi:10.1186/s40798-019-0188-x

Collins, H., Booth, J. N., Duncan, A., Fawkner, S., & Niven, A. (2019). The Effect of Resistance Training Interventions on 'The Self' in Youth: a Systematic Review and Meta-analysis. *Sports Med Open, 5*(1), 29. doi:10.1186/s40798-019-0205-0

Comeras-Chueca, C., Marin-Puyalto, J., Matute-Llorente, A., Vicente-Rodriguez, G., Casajus, J. A., & Gonzalez-Aguero, A. (2021). The Effects of Active Video Games on Health-Related Physical Fitness and Motor Competence in Children and Adolescents with Healthy Weight: A Systematic Review and Meta-Analysis. *International Journal of Environmental Research and Public Health, 18*(13). doi:10.3390/ijerph18136965

Costigan, S. A., Eather, N., Plotnikoff, R. C., Taaffe, D. R., & Lubans, D. R. (2015). High-intensity interval training for improving health-related fitness in adolescents: a systematic review and meta-analysis. *British Journal of Sports Medicine, 49*(19), 1253-1261. doi:10.1136/bjsports-2014-094490

Cox, A., Fairclough, S. J., Kosteli, M. C., & Noonan, R. J. (2020). Efficacy of School-Based Interventions for Improving Muscular Fitness Outcomes in Adolescent Boys: A Systematic Review and Meta-analysis. *Sports Medicine, 50*(3), 543-560. doi:10.1007/s40279-019-01215-5

Craggs, C., Corder, K., van Sluijs, E. M., & Griffin, S. J. (2011). Determinants of change in physical activity in children and adolescents: a systematic review. *American Journal of Preventive Medicine, 40*(6), 645-658. doi:10.1016/j.amepre.2011.02.025

Cuschieri, S. (2019a). The CONSORT statement. *Saudi Journal of Anaesthesia, 13*(Suppl 1), S27-S30. doi:10.4103/sja.SJA_559_18

Cuschieri, S. (2019b). The STROBE guidelines. *Saudi Journal of Anaesthesia, 13*(Suppl 1), S31-S34. doi:10.4103/sja.SJA_543_18

da Silva Bento, A. F. P., Carrasco Páez, L., & de Mendonça Raimundo, A. M. (2022). School-Based High-Intensity Interval Training Programs for Promoting Physical Activity and Fitness in Adolescents: A Systematic Review. *Journal of Teaching in Physical Education, 41*(2), 288-300. doi:10.1123/jtpe.2020-0187

de Andrade Gonçalves, E. C., Augusto Santos Silva, D., & Gimenes Nunes, H. E. (2015). Prevalence and Factors Associated With Low Aerobic Performance Levels in Adolescents: A Systematic Review. *Current Pediatric Reviews, 11*(1), 56-70. doi:10.2174/1573396311666150501003435

De Meester, A., Barnett, L. M., Brian, A., Bowe, S. J., Jimenez-Diaz, J., Van Duyse, F., . . . Haerens, L. (2020). The Relationship Between Actual and Perceived Motor Competence in Children, Adolescents and Young Adults: A Systematic Review and Meta-analysis. *Sports Medicine, 50*(11), 2001-2049. doi:10.1007/s40279-020-01336-2

Duncombe, S. L., Barker, A. R., Bond, B., Earle, R., Varley-Campbell, J., Vlachopoulos, D., . . . Stylianou, M. (2022). School-based high-intensity interval training programs in children and adolescents: A systematic review and meta-analysis. *PloS One, 17*(5), e0266427. doi:10.1371/journal.pone.0266427

Eather, N., Babic, M., Riley, N., Costigan, S. A., & Lubans, D. R. (2022). Impact of Embedding High-Intensity Interval Training in Schools and Sports Training on Children and Adolescent's Cardiometabolic Health and Health-Related Fitness: Systematic Review and Meta-Analysis. *Journal of Teaching in Physical Education, 42*(2), 243-255. doi:10.1123/jtpe.2021-0165

Engel, A. C., Broderick, C. R., van Doorn, N., Hardy, L. L., & Parmenter, B. J. (2018). Exploring the Relationship Between Fundamental Motor Skill Interventions and Physical Activity Levels in Children: A Systematic Review and Meta-analysis. *Sports Medicine, 48*(8), 1845-1857. doi:10.1007/s40279-018-0923-3

Errisuriz, V. L., Golaszewski, N. M., Born, K., & Bartholomew, J. B. (2018). Systematic Review of Physical Education-Based Physical Activity Interventions Among Elementary School Children. *Journal of Primary Prevention, 39*(3), 303-327. doi:10.1007/s10935-018-0507-x

Estevan, I., & Barnett, L. M. (2018). Considerations Related to the Definition, Measurement and Analysis of Perceived Motor Competence. *Sports Medicine, 48*(12), 2685-2694. doi:10.1007/s40279-018-0940-2

Ferreira, F. A., Santos, C. C., Palmeira, A. L., Fernandes, R. J., & Costa, M. J. (2024). Effects of Swimming Exercise on Early Adolescents' Physical Conditioning and Physical Health: A Systematic Review. *J Funct Morphol Kinesiol, 9*(3). doi:10.3390/jfmk9030158

Figueroa, R., & An, R. (2017). Motor Skill Competence and Physical Activity in Preschoolers: A Review. *Matern Child Health J, 21*(1), 136-146. doi:10.1007/s10995-016-2102-1

Fitts, W. H., & Warren, W. L. (1996). *Tennessee self-concept scale: TSCS-2*: Western Psychological Services Los Angeles.

Folio, M. R., & Fewell, R. R. (2000). *PDMS-2: Peabody Development Motor Scales*: Pro-Ed.

Folio, M. R., & Fewell, R. R. (2023). *PDMS-3: Peabody Developmental Motor Scales*: Pro-ed.

Forsman, H., Grasten, A., Blomqvist, M., Davids, K., Liukkonen, J., & Konttinen, N. (2016). Development and validation of the Perceived Game-Specific Soccer Competence Scale. *Journal of Sports Sciences, 34*(14), 1319-1327. doi:10.1080/02640414.2015.1125518

Fox, K. R., & Corbin, C. B. (1989). The Physical Self-Perception Profile - Development and Preliminary Validation. *Journal of Sport & Exercise Psychology, 11*(4), 408-430. doi:DOI 10.1123/jsep.11.4.408

Frankenburg, W. K., Dodds, J., Archer, P., Shapiro, H., & Bresnick, B. (1992). The Denver II: a major revision and restandardization of the Denver Developmental Screening Test. *Pediatrics, 89*(1), 91-97.

Frankenburg, W. K., & Dodds, J. B. (1967). The Denver developmental screening test. *Journal of Pediatrics, 71*(2), 181-191. doi:10.1016/s0022-3476(67)80070-2

Gäbler, M., Prieske, O., Hortobágyi, T., & Granacher, U. (2018). The effects of concurrent strength and endurance training on physical fitness and athletic performance in youth: A systematic review and meta-analysis. *Frontiers in Physiology, 9*(AUG). doi:10.3389/fphys.2018.01057

Gallahue, D. L., Ozmun, J. C., & Goodway, J. (2012). *Understanding Motor Development: Infants, Children, Adolescents, Adults*. Boston, MA: McGraw-Hill.

Garcia-Banos, C., Angel Rubio-Arias, J., Manuel Martinez-Aranda, L., & Jesus Ramos-Campo, D. (2020). Secondary-School-Based Interventions to Improve Muscular Strength in Adolescents: A Systematic Review. *Sustainability, 12*(17). doi:10.3390/su12176814

Garcia-Hermoso, A., Alonso-Martinez, A. M., Ramirez-Velez, R., & Izquierdo, M. (2020). Effects of Exercise Intervention on Health-Related Physical Fitness and Blood Pressure in Preschool Children: A Systematic Review and Meta-Analysis of Randomized Controlled Trials. *Sports Medicine, 50*(1), 187-203. doi:10.1007/s40279-019-01191-w

García-Hermoso, A., Alonso-Martínez, A. M., Ramírez-Vélez, R., Pérez-Sousa, M., Ramírez-Campillo, R., & Izquierdo, M. (2020). Association of Physical Education With Improvement of Health-Related Physical Fitness Outcomes and Fundamental Motor Skills Among Youths: A Systematic Review and Meta-analysis. *Jama Pediatrics, 174*(6), e200223. doi:10.1001/jamapediatrics.2020.0223

Garcia-Hermoso, A., Ezzatvar, Y., Ramirez-Velez, R., Olloquequi, J., & Izquierdo, M. (2021). Is device-measured vigorous physical activity associated with health-related outcomes in children and adolescents? A systematic review and meta-analysis. *Journal of Sport and Health Science, 10*(3), 296-307. doi:10.1016/j.jshs.2020.12.001

Grady, A., Lorch, R., Giles, L., Lamont, H., Anderson, A., Pearson, N., . . . Yoong, S. L. (2025). The impact of early childhood education and care-based interventions on child physical activity, anthropometrics, fundamental movement skills, cognitive functioning, and social–emotional wellbeing: A systematic review and meta-analysis. *Obesity Reviews, 26*(2). doi:10.1111/obr.13852

Graham, M., Azevedo, L., Wright, M., & Innerd, A. L. (2022). The Effectiveness of Fundamental Movement Skill Interventions on Moderate to Vigorous Physical Activity Levels in 5-to 11-Year-Old Children: A Systematic Review and Meta-Analysis. *Sports Medicine, 52*(5), 1067-1090. doi:10.1007/s40279-021-01599-3

Gralla, M. H., McDonald, S. M., Breneman, C., Beets, M. W., & Moore, J. B. (2019). Associations of Objectively Measured Vigorous Physical Activity With Body Composition, Cardiorespiratory Fitness, and Cardiometabolic Health in Youth: A Review. *American Journal of Lifestyle Medicine, 13*(1), 61-97. doi:10.1177/1559827615624417

Griffiths, A., Toovey, R., Morgan, P. E., & Spittle, A. J. (2018). Psychometric properties of gross motor assessment tools for children: a systematic review. *Bmj Open, 8*(10), e021734. doi:10.1136/bmjopen-2018-021734

Gutierrez-Garcia, C., Astrain, I., Izquierdo, E., Teresa Gomez-Alonso, M., & Maria Yague, J. (2018). Effects of judo participation in children: a systematic review. *Ido Movement for Culture-Journal of Martial Arts Anthropology, 18*(4), 63-73. doi:10.14589/ido.18.4.8

Hanna, L., Burns, C., O'Neill, C., & Coughlan, E. (2023). A Systematic Review of the Implementation and Effectiveness of 'The Daily Mile' on Markers of Children's Health. *International Journal of Environmental Research and Public Health, 20*(13). doi:10.3390/ijerph20136203

Harter, S. (1985). Self-perception profile for children. *Hispanic Journal of Behavioral Sciences*.

Harter, S. (1988). Self-perception profile for adolescents. *Gifted Child Quarterly*.

Harter, S. (2012a). Self-perception profile for adolescents: Manual and questionnaires. *Denver, CO: University of Denver, Department of Psychology*, 31-45.

Harter, S. (2012b). Self-perception profile for children: Manual and questionnaires. *Denver, CO: University of Denver, Department of Psychology*, 31-45.

Harter, S., & Pike, R. (1984). The Pictorial Scale of Perceived Competence and Social Acceptance for Young-Children. *Child Development, 55*(6), 1969-1982. doi:Doi 10.2307/1129772

Hassan, M. A., Liu, W., McDonough, D. J., Su, X., & Gao, Z. (2022). Comparative Effectiveness of Physical Activity Intervention Programs on Motor Skills in Children and Adolescents: A Systematic Review and Network Meta-Analysis. *International Journal of Environmental Research and Public Health, 19*(19). doi:10.3390/ijerph191911914

Haywood, K. M., & Getchell, N. (2014). *Lifespan motor development* (6 ed.). Champaign,IL: Human Kinetics.

Henderson, S., & Barnett, A. (2023). Movement Assessment Battery for Children, Third Edition (MABC-3).

Henderson, S., & Sugden, D. (1992). *The Movement Assessment Battery for Children*. London: The Psychological Corporation. .

Henderson, S. E. (2007). *MOvement Assessment Battery for Children—Second edition*. Retrieved from

Henriques-Neto, D., Peralta, M., Garradas, S., Pelegrini, A., Pinto, A. A., Sanchez-Miguel, P. A., & Marques, A. (2020). Active Commuting and Physical Fitness: A Systematic Review. *International Journal of Environmental Research and Public Health, 17*(8). doi:10.3390/ijerph17082721

Herrmann, C. (2018). *MOBAK 1-4. Test zur Erfassung Motorischer Basiskompetenzen für die Klassen 1-4*. Göttingen, Germany Hogrefe Publishing Group.

Hesketh, K. R., O'Malley, C., Paes, V. M., Moore, H., Summerbell, C., Ong, K. K., . . . van Sluijs, E. M. F. (2017). Determinants of Change in Physical Activity in Children 0-6 years of Age: A Systematic Review of Quantitative Literature. *Sports Medicine, 47*(7), 1349-1374. doi:10.1007/s40279-016-0656-0

Holfelder, B., & Schott, N. (2014). Relationship of fundamental movement skills and physical activity in children and adolescents: A systematic review. *Psychology of Sport and Exercise, 15*(4), 382-391. doi:10.1016/j.psychsport.2014.03.005

Hui, L., Wei, S., Luping, Q., & Nannan, G. (2024). Developing the optimal gross movement interventions to improve the physical fitness of 3-10 year-old children: a systematic review and meta-analysis. *Frontiers in Psychology, 15*, 1355821. doi:10.3389/fpsyg.2024.1355821

Jaakkola, T., & Washington, T. (2013). The relationship between fundamental movement skills and self-reported physical activity during Finnish junior high school. *Physical Education and Sport Pedagogy, 18*(5), 492-505. doi:10.1080/17408989.2012.690386

Jetté, M., Sidney, K., & Blümchen, G. (1990). Metabolic equivalents (METS) in exercise testing, exercise prescription, and evaluation of functional capacity. *Clinical Cardiology, 13*(8), 555-565. doi:10.1002/clc.4960130809

Jiang, T., Zhao, G., Fu, J., Sun, S., Chen, R., Chen, D., . . . Hu, H. (2024). Relationship Between Physical Literacy and Cardiorespiratory Fitness in Children and Adolescents: A Systematic Review and Meta-analysis. *Sports medicine (Auckland, N.Z.)*. doi:10.1007/s40279-024-02129-7

Johnstone, A., Hughes, A. R., Martin, A., & Reilly, J. J. (2018). Utilising active play interventions to promote physical activity and improve fundamental movement skills in children: a systematic review and meta-analysis. *BMC Public Health, 18*(1), 789. doi:10.1186/s12889-018-5687-z

Jones, D., Innerd, A., Giles, E. L., & Azevedo, L. B. (2020). Association between fundamental motor skills and physical activity in the early years: A systematic review and meta-analysis. *Journal of Sport and Health Science, 9*(6), 542-552. doi:10.1016/j.jshs.2020.03.001

Kakebeeke, T. H., Knaier, E., Chaouch, A., Caflisch, J., Rousson, V., Largo, R. H., & Jenni, O. G. (2018). Neuromotor development in children. Part 4: new norms from 3 to 18 years. *Developmental Medicine and Child Neurology, 60*(8), 810-819. doi:10.1111/dmcn.13793

Kiphard, E. J., & Schilling, F. (1974). *Körperkoordinationstest für kinder: KTK*. Retrieved from Gottingen:

Kiphard, E. J., & Schilling, F. (2007). *Körperkoordinationstest für Kinder KTK. Manual. 2. überarbeitete und ergänzte Auflage*. Retrieved from Göttingen:

Kiphard, E. J., & Schilling, F. (2017). *KTK - Körperkoordinationstest für Kinder*. Retrieved from Bern, Schwitzerland:

Kuijpers, T., van der Windt, D., van der Heijden, G., & Bouter, L. M. (2004). Systematic review of prognostic cohort studies on shoulder disorders. *Pain, 109*(3), 420-431. doi:10.1016/j.pain.2004.02.017

Lang, J. J., Belanger, K., Poitras, V., Janssen, I., Tomkinson, G. R., & Tremblay, M. S. (2018). Systematic review of the relationship between 20m shuttle run performance and health indicators among children and youth. *Journal of Science and Medicine in Sport, 21*(4), 383-397. doi:10.1016/j.jsams.2017.08.002

Largo, R. H., Caflisch, J. A., Hug, F., Muggli, K., Molnar, A. A., Molinari, L., . . . Gasser, S. T. (2001). Neuromotor development from 5 to 18 years. Part 1: timed performance. *Developmental Medicine and Child Neurology, 43*(7), 436-443. doi:10.1017/s0012162201000810

Larouche, R., Saunders, T. J., Faulkner, G. E. J., Colley, R., & Tremblay, M. (2014). Associations between active school transport and physical activity, body composition, and cardiovascular fitness: A systematic review of 68 studies. *Journal of Physical Activity and Health, 11*(1), 206-227. doi:10.1123/jpah.2011-0345

Lei, Y., & Jun, H.-p. (2022). Does Taekwondo Poomsae Training Impact on Body Composition, Physical Fitness, and Blood Composition in Children and Adolescents? A Systematic Review. *Exercise Science, 31*(1), 11-25.

Li, B., Liu, J., & Ying, B. (2022). Physical education interventions improve the fundamental movement skills in kindergarten: a systematic review and meta-analysis. *Food Science and Technology, 42*. doi:10.1590/fst.46721

Li, Z., Qi, Y., Chen, X., Li, J., Zhang, J., Li, P., & Zhou, Z. (2024). Synergistic Effects of Concurrent Aerobic and Strength Training on Fitness in Children and Adolescents: A Multivariate and Network Meta-Analysis. *Scandinavian Journal of Medicine and Science in Sports, 34*(11), e14764. doi:10.1111/sms.14764

Lin, J., Zhang, R., Shen, J., & Zhou, A. (2022). Effects of school-based neuromuscular training on fundamental movement skills and physical fitness in children: a systematic review. *PeerJ, 10*, e13726. doi:10.7717/peerj.13726

Liu, C., Cao, Y., Zhang, Z., Gao, R., & Qu, G. (2023). Correlation of fundamental movement skills with health-related fitness elements in children and adolescents: A systematic review. *Frontiers in Public Health, 11*, 1129258. doi:10.3389/fpubh.2023.1129258

Liu, W. X., Zeng, N., McDonough, D. J., & Gao, Z. (2020). Effect of Active Video Games on Healthy Children's Fundamental Motor Skills and Physical Fitness: A Systematic Review. *International Journal of Environmental Research and Public Health, 17*(21). doi:ARTN 8264 10.3390/ijerph17218264

Liu, Y., Li, Z., Yuan, L., & Zhou, Z. (2023). The Bidirectional Correlation between Fundamental Motor Skill and Moderate-to-Vigorous Physical Activities: A Systematic Review and Meta-Analysis. *Children (Basel), 10*(9). doi:10.3390/children10091504

Logan, S. W., Barnett, L. M., Goodway, J. D., & Stodden, D. F. (2017). Comparison of performance on process- and product-oriented assessments of fundamental motor skills across childhood. *Journal of Sports Sciences, 35*(7), 634-641. doi:10.1080/02640414.2016.1183803

Logan, S. W., Kipling Webster, E., Getchell, N., Pfeizer, K. A., & Robinson, L. E. (2015). Relationship between fundamental motor skill competence and physical activity during childhood and adolescence: A systematic review. *Kinesiology Reviews, 4*, 416-426.

Logan, S. W., Ross, S. M., Chee, K., Stodden, D. F., & Robinson, L. E. (2018). Fundamental motor skills: A systematic review of terminology. *Journal of Sports Sciences, 36*(7), 781-796. doi:10.1080/02640414.2017.1340660

Lorås, H. (2020). The Effects of Physical Education on Motor Competence in Children and Adolescents: A Systematic Review and Meta-Analysis. *Sports (Basel), 8*(6). doi:10.3390/sports8060088

Lubans, D. R., Boreham, C. A., Kelly, P., & Foster, C. E. (2011). The relationship between active travel to school and health-related fitness in children and adolescents: a systematic review. *The International Journal of Behavioral Nutrition and Physical Activity, 8*, 5. doi:10.1186/1479-5868-8-5

Lubans, D. R., Morgan, P. J., Cliff, D. P., Barnett, L. M., & Okely, A. D. (2010). Fundamental Movement Skills in Children and Adolescents Review of Associated Health Benefits. *Sports Medicine, 40*(12), 1019-1035. doi:Doi 10.2165/11536850-000000000-00000

Marsh, H. W. (1990). *Self description questionnaire-I (SDQI)*. Retrieved from psycnet.apa.org:

Marsh, H. W. (1996). Construct validity of physical self-description questionnaire responses: Relations to external criteria. *Journal of Sport & Exercise Psychology, 18*(2), 111-131. doi:DOI 10.1123/jsep.18.2.111

Martin-Smith, R., Cox, A., Buchan, D. S., Baker, J. S., Grace, F., & Sculthorpe, N. (2020). High Intensity Interval Training (HIIT) Improves Cardiorespiratory Fitness (CRF) in Healthy, Overweight and Obese Adolescents: A Systematic Review and Meta-Analysis of Controlled Studies. *International Journal of Environmental Research and Public Health, 17*(8). doi:10.3390/ijerph17082955

Martinez-Merino, N., & Rico-González, M. (2024). Effects of Physical Education on Preschool Children's Physical Activity Levels and Motor, Cognitive, and Social Competences: A Systematic Review. *Journal of Teaching in Physical Education, 43*(4), 696-706. doi:10.1123/jtpe.2023-0183

McCarron, L. T. (1997). *MAND : McCarron assessment of neuromuscular development, fine and gross motor abilities* (Rev ed.). Dallas, Tex.: McCarron-Dial Systems, Inc.

McDonough, D. J., Liu, W., & Gao, Z. (2020). Effects of Physical Activity on Children's Motor Skill Development: A Systematic Review of Randomized Controlled Trials. *Biomed Res Int, 2020*, 8160756. doi:10.1155/2020/8160756

McGrane, B., Belton, S., Powell, D., Woods, C. B., & Issartel, J. (2016). Physical self-confidence levels of adolescents: Scale reliability and validity. *Journal of Science and Medicine in Sport, 19*(7), 563-567. doi:10.1016/j.jsams.2015.07.004

Minatto, G., Barbosa Filho, V. C., Berria, J., & Petroski, E. L. (2016). School-Based Interventions to Improve Cardiorespiratory Fitness in Adolescents: Systematic Review with Meta-analysis. *Sports Medicine, 46*(9), 1273-1292. doi:10.1007/s40279-016-0480-6

Moon, J., Webster, C. A., Stodden, D. F., Brian, A., Mulvey, K. L., Beets, M., . . . Russ, L. (2024). Systematic review and meta-analysis of physical activity interventions to increase elementary children's motor competence: a comprehensive school physical activity program perspective. *BMC Public Health, 24*(1), 826. doi:10.1186/s12889-024-18145-1

Moran, J., Sandercock, G., Ramirez-Campillo, R., Clark, C. C. T., Fernandes, J. F. T., & Drury, B. (2018). A Meta-Analysis of Resistance Training in Female Youth: Its Effect on Muscular Strength, and Shortcomings in the Literature. *Sports Medicine, 48*(7), 1661-1671. doi:10.1007/s40279-018-0914-4

Neil-Sztramko, S. E., Caldwell, H., & Dobbins, M. (2021). School-based physical activity programs for promoting physical activity and fitness in children and adolescents aged 6 to 18. *Cochrane Database of Systematic Reviews, 9*(9), CD007651. doi:10.1002/14651858.CD007651.pub3

Norris, E., Hamer, M., & Stamatakis, E. (2016). Active video games in schools and effects on physical activity and health: A systematic review. *Journal of Pediatrics, 172*, 40-46e45. doi:10.1016/j.jpeds.2016.02.001

Øglund, G. P., Hildebrand, M., & Ekelund, U. (2015). Are Birth Weight, Early Growth, and Motor Development Determinants of Physical Activity in Children and Youth? A Systematic Review and Meta-Analysis. *Pediatric Exercise Science, 27*(4), 441-453. doi:10.1123/pes.2015-0041

Oppici, L., Stell, F. M., Utesch, T., Woods, C. T., Foweather, L., & Rudd, J. R. (2022). A Skill Acquisition Perspective on the Impact of Exergaming Technology on Foundational Movement Skill Development in Children 3-12 Years: A Systematic Review and Meta-analysis. *Sports Medicine-Open, 8*(1). doi:ARTN 148 10.1186/s40798-022-00534-8

Page, M. J., Moher, D., Bossuyt, P. M., Boutron, I., Hoffmann, T. C., Mulrow, C. D., . . . McKenzie, J. E. (2021). PRISMA 2020 explanation and elaboration: updated guidance and exemplars for reporting systematic reviews. *BMJ, 372*, n160. doi:10.1136/bmj.n160

Payne, V. G., & Isaacs, L. D. (2017). *Human motor development: A lifespan approach*: Routledge.

Peralta, M., Henriques-Neto, D., Gouveia É, R., Sardinha, L. B., & Marques, A. (2020). Promoting health-related cardiorespiratory fitness in physical education: A systematic review. *PloS One, 15*(8), e0237019. doi:10.1371/journal.pone.0237019

Pinho, C. D. F., Bagatini, N. C., Lisboa, S. D. C., Mello, J. B., & Cunha, G. d. S. (2024). Effects of different supervised and structured physical exercise on the physical fitness trainability of children and adolescents: a meta-analysis and meta-regression: Physical fitness trainability in children and adolescents' health. *BMC Pediatrics, 24*(1). doi:10.1186/s12887-024-04929-2

Poitras, V. J., Gray, C. E., Borghese, M. M., Carson, V., Chaput, J. P., Janssen, I., . . . Tremblay, M. S. (2016). Systematic review of the relationships between objectively measured physical activity and health indicators in school-aged children and youth. *Applied Physiology, Nutrition, and Metabolism. Physiologie Appliquée, Nutrition et Métabolisme, 41*(6 Suppl 3), S197-239. doi:10.1139/apnm-2015-0663

Pozuelo-Carrascosa, D. P., García-Hermoso, A., Álvarez-Bueno, C., Sánchez-López, M., & Martinez-Vizcaino, V. (2018). Effectiveness of school-based physical activity programmes on cardiorespiratory fitness in children: a meta-analysis of randomised controlled trials. *British Journal of Sports Medicine, 52*(19), 1234-1240. doi:10.1136/bjsports-2017-097600

Ramirez-Campillo, R., Sortwell, A., Moran, J., Afonso, J., Clemente, F. M., Lloyd, R. S., . . . Granacher, U. (2023). Plyometric-Jump Training Effects on Physical Fitness and Sport-Specific Performance According to Maturity: A Systematic Review with Meta-analysis. *Sports Med Open, 9*(1), 23. doi:10.1186/s40798-023-00568-6

Reyes-Amigo, T., Gomez, M., Gallardo, M., & Palmeira, A. (2017). Effectiveness of high-intenisty interval training on cardiorespiratory fitness and body composition in preadolescents: a systematic review. *European Journal of Human Movement, 39*, 32-47.

Rico-González, M. (2023). The Effect of Primary School-Based Physical Education Programs: A Systematic Review of Randomized Controlled Trials. *Journal of Physical Activity & Health, 20*(4), 317-347. doi:10.1123/jpah.2022-0452

Ridley, K., Ainsworth, B. E., & Olds, T. S. (2008). Development of a compendium of energy expenditures for youth. *The International Journal of Behavioral Nutrition and Physical Activity, 5*, 45. doi:10.1186/1479-5868-5-45

Robinson, L. E., Stodden, D. F., Barnett, L. M., Lopes, V. P., Logan, S. W., Rodrigues, L. P., & D'Hondt, E. (2015). Motor Competence and its Effect on Positive Developmental Trajectories of Health. *Sports Medicine, 45*(9), 1273-1284. doi:10.1007/s40279-015-0351-6

Rudd, J., Butson, M. L., Barnett, L., Farrow, D., Berry, J., Borkoles, E., & Polman, R. (2016). A holistic measurement model of movement competency in children. *Journal of Sports Sciences, 34*(5), 477-485. doi:10.1080/02640414.2015.1061202

Ryckman, R. M., Robbins, M. A., Thornton, B., & Cantrell, P. (1982). Development and Validation of a Physical Self-Efficacy Scale. *Journal of Personality and Social Psychology, 42*(5), 891-900. doi:Doi 10.1037/0022-3514.42.5.891

Santos, C., Burnay, C., Button, C., & Cordovil, R. (2023). Effects of Exposure to Formal Aquatic Activities on Babies Younger Than 36 Months: A Systematic Review. *International Journal of Environmental Research and Public Health, 20*(8). doi:10.3390/ijerph20085610

SHAPE America. (2018). *PE Metrics, 3rd Edition: Assessing Student Performance Using the National Standards & Grade-Level Outcomes for K-12 Physical Education* (9781492526667). Retrieved from Champaign,IL: <https://books.google.nl/books?id=G8NKDwAAQBAJ>

SHAPE America, Couturier, L., Chepko, S., & Holt, S. A. (2014). *National standards & grade-level outcomes for K-12 physical education* (1450496261). Retrieved from Champaign, IL:

Shavelson, R. J., Hubner, J. J., & Stanton, G. C. (1976). Self-Concept - Validation of Construct Interpretations. *Review of Educational Research, 46*(3), 407-441. doi:Doi 10.3102/00346543046003407

Sinclair, L., & Roscoe, C. M. P. (2023). The Impact of Swimming on Fundamental Movement Skill Development in Children (3-11 Years): A Systematic Literature Review. *Children (Basel), 10*(8). doi:10.3390/children10081411

Singh, U., Ramachandran, A. K., Ramirez-Campillo, R., Perez-Castilla, A., Afonso, J., Manuel Clemente, F., & Oliver, J. (2022). Jump rope training effects on health- and sport-related physical fitness in young participants: A systematic review with meta-analysis. *Journal of Sports Sciences, 40*(16), 1801-1814. doi:10.1080/02640414.2022.2099161

Smith, J. J., Eather, N., Weaver, R. G., Riley, N., Beets, M. W., & Lubans, D. R. (2019). Behavioral Correlates of Muscular Fitness in Children and Adolescents: A Systematic Review. *Sports Medicine, 49*(6), 887-904. doi:10.1007/s40279-019-01089-7

Stojanović, S., Andrieieva, O., & Trajković, N. (2024). Associations between number of steps and health outcomes in children and adolescents: a systematic review and meta-analysis. *BMC Public Health, 24*(1), 3310. doi:10.1186/s12889-024-20835-9

Sun, C., Pezic, A., Tikellis, G., Ponsonby, A. L., Wake, M., Carlin, J. B., . . . Dwyer, T. (2013). Effects of school-based interventions for direct delivery of physical activity on fitness and cardiometabolic markers in children and adolescents: a systematic review of randomized controlled trials. *Obesity Reviews, 14*(10), 818-838. doi:10.1111/obr.12047

Sun, S., & Chen, C. (2024). The Effect of Sports Game Intervention on Children's Fundamental Motor Skills: A Systematic Review and Meta-Analysis. *Children-Basel, 11*(2). doi:10.3390/children11020254

Szeszulski, J., Lorenzo, E., Shaibi, G. Q., Buman, M. P., Vega-López, S., Hooker, S. P., & Lee, R. E. (2019). Effectiveness of early care and education center-based interventions for improving cardiovascular fitness in early childhood: A systematic review and meta-analysis. *Prev Med Rep, 15*, 100915. doi:10.1016/j.pmedr.2019.100915

Timmons, B. W., LeBlanc, A. G., Carson, V., Gorber, S. C., Dillman, C., Janssen, I., . . . Tremblay, M. S. (2012). Systematic review of physical activity and health in the early years (aged 0-4 years). *Applied Physiology Nutrition and Metabolism, 37*(4), 773-792. doi:10.1139/H2012-070

Ulrich, D. A. (1985). *Test of gross motor development*. Retrieved from Austin, TX:

Ulrich, D. A. (2000). *Test of gross motor development-2*. Retrieved from Austin,TX:

Ulrich, D. A. (2013). The test of gross motor development-3 (TGMD-3): Administration, scoring, and international norms. *Spor Bilimleri Dergisi, 24*(2), 27-33.

Utesch, T., Bardid, F., Busch, D., & Strauss, B. (2019). The Relationship Between Motor Competence and Physical Fitness from Early Childhood to Early Adulthood: A Meta-Analysis. *Sports Medicine, 49*(4), 541-551. doi:10.1007/s40279-019-01068-y

Van Capelle, A., Broderick, C. R., van Doorn, N., R, E. W., & Parmenter, B. J. (2017). Interventions to improve fundamental motor skills in pre-school aged children: A systematic review and meta-analysis. *Journal of Science and Medicine in Sport, 20*(7), 658-666. doi:10.1016/j.jsams.2016.11.008

Veldman, S. L. C., Chin, A. P. M. J. M., & Altenburg, T. M. (2021). Physical activity and prospective associations with indicators of health and development in children aged <5 years: a systematic review. *The International Journal of Behavioral Nutrition and Physical Activity, 18*(1), 6. doi:10.1186/s12966-020-01072-w

Villa-González, E., Barranco-Ruiz, Y., García-Hermoso, A., & Faigenbaum, A. D. (2023). Efficacy of school-based interventions for improving muscular fitness outcomes in children: A systematic review and meta-analysis. *Eur J Sport Sci, 23*(3), 444-459. doi:10.1080/17461391.2022.2029578

Viswanathan, M., & Berkman, N. D. (2012). Development of the RTI item bank on risk of bias and precision of observational studies. *Journal of Clinical Epidemiology, 65*(2), 163-178. doi:10.1016/j.jclinepi.2011.05.008

Wang, L., & Zhou, Y. (2023). A Systematic Review of Correlates of the Moderate-to-Vigorous Physical Activity of Students in Elementary School Physical Education. *Journal of Teaching in Physical Education, 42*(1), 44-59. doi:10.1123/jtpe.2020-0197

Wang, X., & Zhou, B. (2024). Motor development-focused exercise training enhances gross motor skills more effectively than ordinary physical activity in healthy preschool children: an updated meta-analysis. *Frontiers in Public Health, 12*, 1414152. doi:10.3389/fpubh.2024.1414152

Welk, G. (2002). *Physical activity assessments for health-related research*. Champaign,IL: Human Kinetics Publishers, Inc.

Whitehead, J. R. (1995). A Study of Children’s Physical Self-Perceptions Using an Adapted Physical Self-Perception Profile Questionnaire. *Pediatric Exercise Science, 7*(2), 132-151. doi:10.1123/pes.7.2.132

Wijndaele, K., Lakshman, R., Landsbaugh, J. R., Ong, K. K., & Ogilvie, D. (2009). Determinants of early weaning and use of unmodified cow's milk in infants: a systematic review. *Journal of the American Dietetic Association, 109*(12), 2017-2028. doi:10.1016/j.jada.2009.09.003

Williams, H. G., Pfeiffer, K. A., Dowda, M., Jeter, C., Jones, S., & Pate, R. R. (2009). A Field-Based Testing Protocol for Assessing Gross Motor Skills in Preschool Children: The CHAMPS Motor Skills Protocol (CMSP). *Meas Phys Educ Exerc Sci, 13*(3), 151-165. doi:10.1080/10913670903048036

Woodforde, J., Alsop, T., Salmon, J., Gomersall, S., & Stylianou, M. (2022). Effects of school-based before-school physical activity programmes on children's physical activity levels, health and learning-related outcomes: a systematic review. *British Journal of Sports Medicine, 56*(13), 740-754. doi:10.1136/bjsports-2021-104470

Wu, C., Xu, Y., Chen, Z., Cao, Y., Yu, K., & Huang, C. (2021). The Effect of Intensity, Frequency, Duration and Volume of Physical Activity in Children and Adolescents on Skeletal Muscle Fitness: A Systematic Review and Meta-Analysis of Randomized Controlled Trials. *International Journal of Environmental Research and Public Health, 18*(18). doi:10.3390/ijerph18189640

Wu, J., Yang, Y., Yu, H., Li, L., Chen, Y., & Sun, Y. (2023). Comparative effectiveness of school-based exercise interventions on physical fitness in children and adolescents: a systematic review and network meta-analysis. *Frontiers in Public Health, 11*, 1194779. doi:10.3389/fpubh.2023.1194779

Xin, F., Chen, S. T., Clark, C., Hong, J. T., Liu, Y., & Cai, Y. J. (2020). Relationship between Fundamental Movement Skills and Physical Activity in Preschool-Aged Children: A Systematic Review. *International Journal of Environmental Research and Public Health, 17*(10). doi:10.3390/ijerph17103566

Xu, Z., Shen, S.-J., & Wen, Y.-H. (2024). The relationship between fundamental movement skills and physical activity in preschoolers: a systematic review. *Early Child Development and Care, 194*(2), 323-334. doi:10.1080/03004430.2024.2309478

Yang, C.-C., & Hsu, Y.-L. (2010). A review of accelerometry-based wearable motion detectors for physical activity monitoring. *Sensors, 10*(8), 7772-7788.

Zamorano-Garcia, D., Infantes-Paniagua, A., Cuevas-Campos, R., & Fernandez-Bustos, J. G. (2023). Impact of Physical Activity-Based Interventions on Children and Adolescents' Physical Self-Concept: A Meta-Analysis. *Research Quarterly for Exercise and Sport, 94*(1), 1-14. doi:10.1080/02701367.2021.1927945

Zeng, N., Ayyub, M., Sun, H., Wen, X., Xiang, P., & Gao, Z. (2017). Effects of Physical Activity on Motor Skills and Cognitive Development in Early Childhood: A Systematic Review. *Biomed Res Int, 2017*, 2760716. doi:10.1155/2017/2760716

Zhang, D., Soh, K. G., Chan, Y. M., Feng, X., Bashir, M., & Xiao, W. (2024). Effect of functional training on fundamental motor skills among children: A systematic review. *Heliyon, 10*(23), e39531. doi:10.1016/j.heliyon.2024.e39531

Zhao, Q., Wang, Y., Niu, Y., & Liu, S. (2023). Jumping Rope Improves the Physical Fitness of Preadolescents Aged 10-12 Years: A Meta-Analysis. *Journal of Sports Science & Medicine, 22*(2), 367-380. doi:10.52082/jssm.2023.367

Zhou, X., Li, J., & Jiang, X. (2024). Effects of different types of exercise intensity on improving health-related physical fitness in children and adolescents: a systematic review. *Scientific Reports, 14*(1), 14301. doi:10.1038/s41598-024-64830-x

Zhu, W., Rink, J., Placek, J. H., Graber, K. C., Fox, C., Fisette, J. L., . . . Raynes, D. (2011). PE Metrics: Background, Testing Theory, and Methods. *Measurement in Physical Education and Exercise Science, 15*(2), 87-99. doi:10.1080/1091367x.2011.568363
